# A single dose of azithromycin versus placebo for malaria in infants: A cluster-randomized trial

**DOI:** 10.1371/journal.pgph.0006518

**Published:** 2026-08-20

**Authors:** Rikhard Ihamuotila, Jane Juma, Fadima Haidara, Oumar Samaké, Juho Luoma, Laura Adubra, Yin Bun Cheung, Dagmar Alber, Awa Traoré, Camilla Ducker, Ulla Ashorn, Per Ashorn, Samba Sow, Yue-Mei Fan

**Affiliations:** 1 Center for Child, Adolescent and Maternal Health Research, Faculty of Medicine and Health Technology, Tampere University and Tampere University Hospital, Tampere, Finland; 2 Center for Vaccine Development–Mali, Bamako, Mali; 3 Great Ormond Street Institute of Child Health, University College London, London, United Kingdom; 4 Tro Da Ltd, Caerphilly, United Kingdom; Malaria Research and Training Center, University of Science, Techniques and Technology of Bamako, MALI

## Abstract

Azithromycin mass drug administration (MDA) has been shown to promote child survival in high mortality settings. Azithromycin has antimalarial effects, and one possible mechanism behind mortality reduction with azithromycin may be reduction in malaria. We aimed to test the effect of a single dose of azithromycin on malaria prevalence and parasite load in infants. This malaria substudy was part of a large double-blinded cluster-randomized clinical trial in Mali studying the effect of azithromycin MDA on infant mortality (ClinicalTrials.gov NCT04424511). Villages were randomized into either placebo or azithromycin MDA groups. We collected dry blood spot samples among 4–11-month-old infants right before and two weeks after an azithromycin MDA dose (20mg/kg). Malaria positivity and concentration of *Plasmodium falciparum* DNA (parasite load) were determined by quantitative PCR. Hemoglobin concentration was measured with a rapid test. A total of 1158 infants at baseline were treated, with 324 infants in 20 villages in the control group, and 834 infants in 38 villages in the azithromycin group. At baseline, prevalence of PCR-positive malaria was 9.4% in the control group and 9.9% in the azithromycin group. On day 14 after receiving the study drug, prevalence was 8.9% among controls and 8.0% in the azithromycin group. Adjusting for baseline and clusters, malaria prevalence risk ratio was 0.96 (95% CI: 0.63 to 1.48) favoring azithromycin. The prevalence risk ratio for baseline malaria PCR-positive infants was 0.81 (0.55 to 1.19). The ratio of means for parasite load was 0.91 (0.66 to 1.25) favoring azithromycin. Hemoglobin was 0.1 g/L (-3.9 to 4.1) higher with azithromycin. The findings do not conclusively support, but are consistent with, a hypothesis that a single dose of azithromycin reduces malaria prevalence and parasite load among infants in a malaria-endemic West African setting.

## Introduction

Mass drug administration (MDA) of azithromycin to under 5-year-old children is a promising intervention to reduce child mortality in sub-Saharan Africa [1–4]. While the intervention has been shown to reduce pneumonia, diarrhea, malaria in under 5-year-old children [5–8], the effect on these conditions has been marginal and the mechanism behind mortality reduction remains unclear. To mitigate the development of antimicrobial resistance, the 2020 WHO guideline on azithromycin MDA for child survival recommended giving the intervention only to 1–11-month-old infants [9], prompting large trials to test the efficacy when given only to this subgroup [3,10–12].

Malaria remains one of the main causes of child mortality, and reduction of malaria burden is a plausible mechanism behind mortality reduction with azithromycin [13]. Although weak, the clinical antimalarial effect of azithromycin is well established [14–16]. Azithromycin has a proven effect on the malaria parasite apicoplast organelle [17], but may also inhibit red blood cell invasion, and mosquito stage development [18,19]. Some azithromycin MDA trials have seen well over 20% reduction in malaria prevalence, parasite density, malaria mortality and clinic visits [6,20–23]. Conversely, many trials have reported no effect on malaria [24–26]. Discerning the effect of azithromycin MDA on malaria in areas with a seasonal malaria chemoprevention (SMC) program has also proven difficult [24,25,27–29].

Evidence is scant on the effect of azithromycin MDA on malaria prevalence and parasite load in the WHO-recommended infant subgroup. We aimed to test the short-term effect of one dose of azithromycin on malaria prevalence and parasite load among infants in a malaria-endemic West African setting.

## Methods

### Ethics statement

Ethical approval for the LAKANA trial was received from the Mali Institutional Review Board (IRB), the *Comité d’Éthique de l’Université des Sciences, des Techniques et des Technologies de Bamako (USTBB),* (IRB approval number: N2019/181/CE/FMPOS/FAPH, initially approved 27 December 2019, with the most recent reapproval granted on 13 October 2025). Additionally, the Ethics Committee of Pirkanmaa Hospital District gave a favorable opinion for Tampere University researchers to participate in conducting the LAKANA trial. The trial followed the Declaration of Helsinki and Good Clinical Practice guidelines.

Informed consent was received on three levels: village level, household level, and caregiver level. Verbal consent by household heads or proxies was documented with a digital signature. The caregiver gave final consent for treatment. Substudy-specific information was given to those participating in this substudy and written or fingerprint signed guardian consent was sought.

### Trial design

This cluster-randomized placebo-controlled trial was conducted as a prespecified substudy in a subset of villages within a large-scale trial in Western Mali testing the effect of azithromycin MDA on infant mortality: the LAKANA trial (ClinicalTrials.gov NCT04424511, registered 11 June 2020) [30]. The study protocol, statistical analysis plan, and all statistical operating procedures for the main trial and this substudy have been published prospectively [31–33].

The LAKANA trial randomized villages (clusters) into three trial arms: placebo, azithromycin MDA two times yearly, or azithromycin MDA four times yearly (quarterly). The villages were randomized into these three arms in a 3:4:2 ratio respectively. The data collection for this study (February 17 to May 30, 2022, dry season) was conducted during a single MDA round. The villages were visited every three months in the LAKANA trial, and the data collection for this substudy happened on the fourth MDA round, nine months after village enrollment. We consolidated the twice yearly and quarterly azithromycin arms into one treatment group, because villages in both azithromycin arms were receiving azithromycin at the time of this substudy MDA round.

### Randomization and blinding

The LAKANA study villages were randomized into the three intervention arms in a public allocation event. Each village representative pulled a lottery ticket from a container with a letter code. The letter code defined the trial arm for the village. The participants, their guardians, village leaders, study site and laboratory personnel, and investigators were all masked. Only external partners facilitating the randomization were unmasked. The placebo and azithromycin substances and their packaging were indistinguishable. Further details of the randomization and masking have been published in the main trial report [30].

### Eligibility

The 59 LAKANA substudy villages were in Kita region in Western Mali and were chosen due to their proximity to the Kita study headquarters, facilitating the timely transportation and storage of biological samples. All infants aged 4–11 months (120–364 days of age) in these villages were eligible for this substudy. Infants with severe illness, known macrolide allergy, or weighing less than 3.0 kg were excluded. Severely ill infants were excluded and referred to the nearest health facility.

### Intervention and sample collection

Data collectors visited all households included in the parent trial and identified eligible infants. The infants and caregivers were accompanied to a temporary village facility, where the study drug was administered and baseline blood samples were obtained. Infants were weighed with an electronic scale (*ADE Model M111600-01, Germany*) and given reconstituted suspension of azithromycin 20mg/kg or placebo orally. Caregivers were asked to inform about any adverse events within 14 days of receiving the drug.

The study nurse performed a heel prick with a small lancet to obtain blood drops. At least two blood spots were applied to a dry blood spot (DBS) collection card (*PerkinElmer PKI RUO spot Saver Card, USA*). Blood hemoglobin (Hb) concentration was determined from a heel prick blood drop using a bedside kit (*Orion QuickRead go, Finland*), which gave immediate results. The DBS cards were air-dried for 3 hours on a drying rack and then placed in individually sealed plastic bags with a desiccant bag (*QIAGEN WB100003, Germany*) to prevent humidification. The sample bags were transported to Bamako laboratory without cold storage within 1–2 days from sample collection. If Hb was lower than 80 g/L, the child was referred to the nearest health facility.

The data collectors returned to the village after 14 days, identified the infants and accompanied them to the temporary village facility, where blood samples were obtained again. Caregivers were interviewed on day 14 about adverse events. LAKANA adverse events were reported in the main trial report [30].

### Outcomes

The outcomes were malaria prevalence (PCR-detected parasitemia), mean parasite load (DNA concentration in ng/μL as a proxy for disease severity and parasites per blood volume), and mean hemoglobin concentration (g/L) [34,35]. Variables tested as possible effect modifiers were infant age, sex, weight-for-age z-score (WAZ), household asset index, and water, sanitation, and hygiene (WASH) index. The asset and WASH indices are described in more detail in the trial statistical analysis plan [31].

### Laboratory analyses

The DNA extraction of *Plasmodium falciparum* was carried out using the Extracta DBS reagent (*Quantabio BioSciences*), a ready-to-use protocol optimized for PCR-ready DNA recovery from DBS. We performed quantitative real-time polymerase chain reaction (PCR) to amplify the lactate dehydrogenase (LDH) gene, a marker of *Plasmodium falciparum,* in the isolated DNA. Reaction volumes were 25 µl each, consisting of 12.5 µl Master Mix, 5 µl of gDNA, forward and reverse primers, probe, and molecular-grade water. Genomic DNA (10 µg/mL) from *Plasmodium falciparum* strain 3D7 (*MRA-102G, BEI Resource, USA*) was used to generate serial dilutions for the qPCR standard curve. All reactions were run in duplicate. Samples were considered positive if both had a cycle threshold (Ct) value of < 38 [36,37]. If the two results had discrepancies, we repeated the PCR. The blood DNA concentration of *Plasmodium falciparum* as a proxy for parasite density and disease severity was derived from real-time PCR standard curves, as validated by others [34,35,38].

### Sample size

We prospectively estimated the 59 substudy villages to have 333–450 infants in each study arm and 1000–1350 infants aged 1–11 months in total. The twice-yearly and quarterly yearly azithromycin arms were pooled. This offered an expected statistical power of 80% to detect an effect size of 0.2-0.3 standard deviations comparing azithromycin and placebo at 5% two-sided type 1 error rate for each outcome, given the inter-cluster correlation coefficient was not larger than 0.03 [31]. One substudy village served as a pilot village for testing substudy data and sample collection and was therefore excluded from analyses.

### Statistical analysis

We used mixed-effects regression models with random intercepts for villages to estimate the effect of the intervention on malaria prevalence, Hb concentration, and malaria parasite load (logistic model for prevalence and linear models for continuous variables). Marginal risk ratios were predicted from the obtained model coefficients. The independent variables were baseline outcome measures (PCR-positivity, parasite load, or Hb) and intervention group. The day 14 outcome measures were the dependent variables. For the effect modifier analyses we accounted for interactions between the compared subgroups and tested the statistical significance of interactions (predefined p-for-interactions <0.1). All statistical analyses were done with R programming language version 4.5.2.

Because parasite load data distributions were highly skewed, we reported geometric means for parasite load and used log-transformed parasite load values in the regression model. Because log-transformation cannot accommodate zero values, malaria negative samples were assigned a value equal to half of the lowest serial dilution (0.000005 ng/µl of *Plasmodium falciparum* DNA). This approach, commonly used in antibody titer studies, was applied to include participants who were malaria negative on day 0 or 14 [39].

## Results

**Fig 1:**
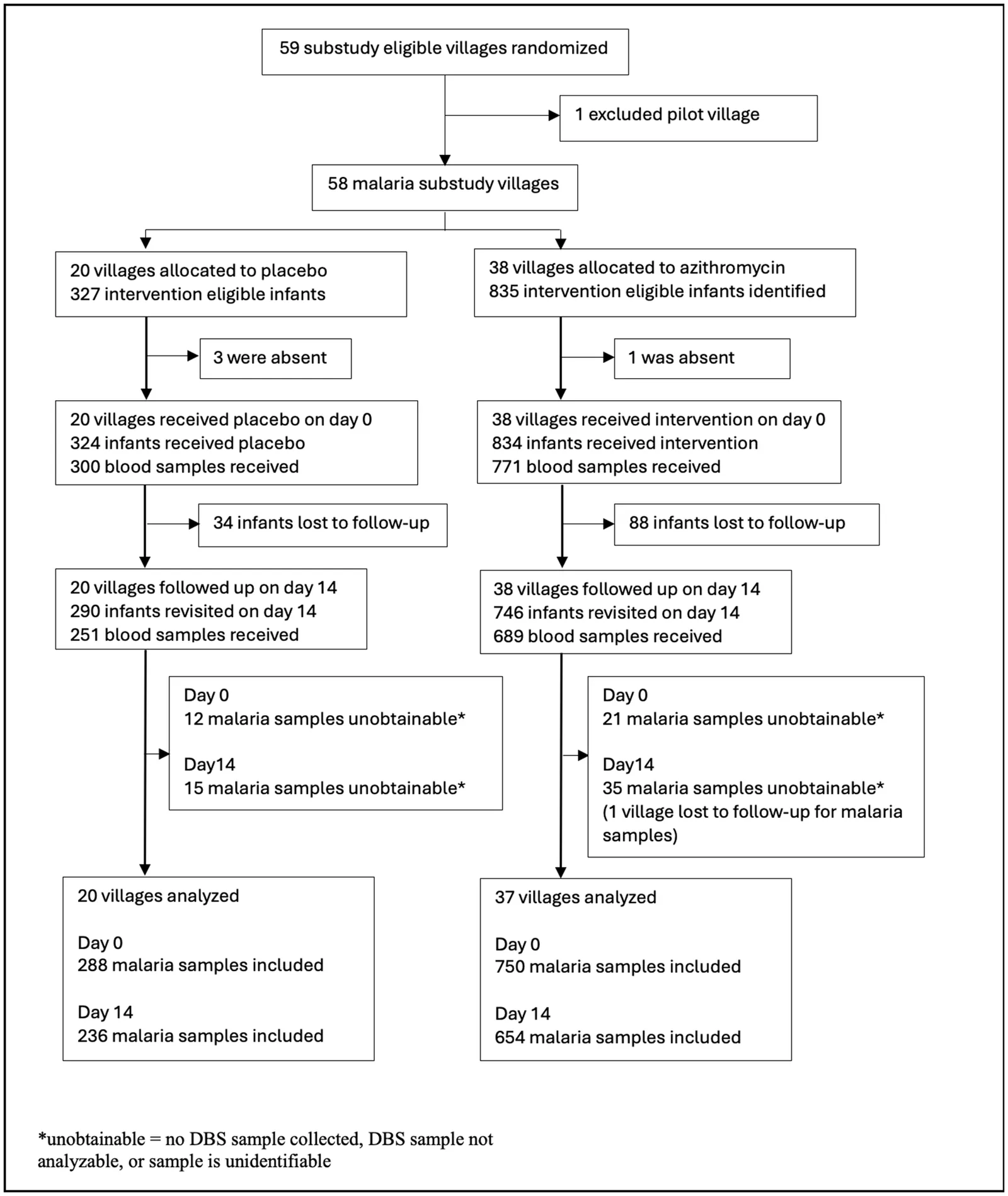
Participant flow chart.

A total of 1158 infants from 58 LAKANA villages were eligible for this malaria substudy (Fig 1). Malaria status at baseline (day 0) was available for 1038 infants, of whom 101 were malaria PCR-positive. At baseline, malaria prevalence was 9.4% in the control group and 9.9% in the azithromycin group (Table 1). Mean parasite load at baseline was 0.0056 ng/μL (geometric mean 0.0000089 ng/μL) in the control group and 0.0036 ng/μL (geometric mean 0.0000101 ng/μL) in the azithromycin group, with values ranging from 0.000054 to 0.5881 ng/μL among malaria PCR-positives. Mean Hb concentrations at baseline were 103 g/L in the control group and 99 g/L in the azithromycin group, with values ranging from 50 to 224 g/L. Severe anemia (Hb < 70 g/L) was observed in 8.6% of infants at baseline.

**Table 1:** Baseline characteristics (day 0) of infants by treatment group.

Variable	Control	Azithromycin
**Number of infants**	324	834
**Number of villages**	20	38
**Average number of infants per village (SD)**	16.2 (8.9)	21.9 (22.5)
**Proportion of girls, %**	51.5	48.3
**Mean (SD) age, months**	7.8 (2.4)	7.7 (2.3)
**Prevalence of PCR-positive malaria, %**	9.4	9.9
**Mean (SD) Hb, g/L**	103 (26)	99 (24)
**Mean (SD) parasite load, 10**^**–3**^ **ng/μL**	5.6 (44.7)	3.6 (26.8)
**Proportion with self-reported fever in preceding 2 weeks, %**	0.0	1.1
**Proportion of infants who received SMC in the past 6 months, %**	0.6	1.4

SD = standard deviation, SMC = seasonal malaria chemoprevention, Hb= hemoglobin.

A total of 1036 infants were revisited on day 14, with 890 malaria results available. Infants were revisited on average 16.0 (3.1SD) days after receiving the study drug (range: 8–30 days). Loss to follow-up rates for malaria results were 21.9% in the control group and 15.3% in the azithromycin group. One azithromycin group village with only one eligible infant was lost to follow-up for malaria outcomes.

### Primary outcomes

On day 14, malaria prevalence was 8.9% in the control group and 8.0% in the azithromycin group, with mean parasite loads of 0.0052 ng/μL (geometric mean 0.0000094 ng/μL) and 0.0038 ng/μL (geometric mean 0.0000087 ng/μL) respectively (Table 2). There was no statistically significant difference in malaria prevalence with azithromycin compared to placebo (Table 2). Adjusted for clusters and baseline values, malaria parasite load was 9% lower with azithromycin (ratio of geometric means 0.91, 95% CI: 0.66 to 1.25), but the result was not statistically significant (p = 0.56). There was no statistically significant difference in Hb concentration with azithromycin.

**Table 2:** Malaria prevalence, parasite load, and Hb concentration at day 14 by treatment group.

Variable	Control	Azithromycin	Risk ratio^a^ estimate (95% CI)	p-value
**Prevalence of PCR-positive malaria, %**	8.9	8.0	0.96 (0.63 to 1.48)	0.86
**Prevalence of PCR-positive malaria among baseline malaria positives, %**	66.7	55.7	0.81 (0.55 to 1.19)	0.28
			**Ratio of geometric** **means**^**b**^ **estimate (95% CI)**	
**Mean (SD) parasite load, 10**^**–3**^ **ng/μL**	5.2 (32.2)	3.8 (43.5)	0.91 (0.66 to 1.25)	0.56
			**Difference in means estimate (95% CI)**	
**Mean (SD) Hb concentration, g/L**	99 (24)	99 (21)	0.1 g/L (-3.9 to 4.1)	0.97

SD = standard deviation, Hb= hemoglobin

^a^Ratio estimates account for baseline values and are regression coefficients calculated with a mixed-effects regression model with random intercepts for clusters.

^b^Ratio of means estimate uses geometric means due to log-transformation.

### Effect modifiers and post-hoc analyses

**Fig 2:**
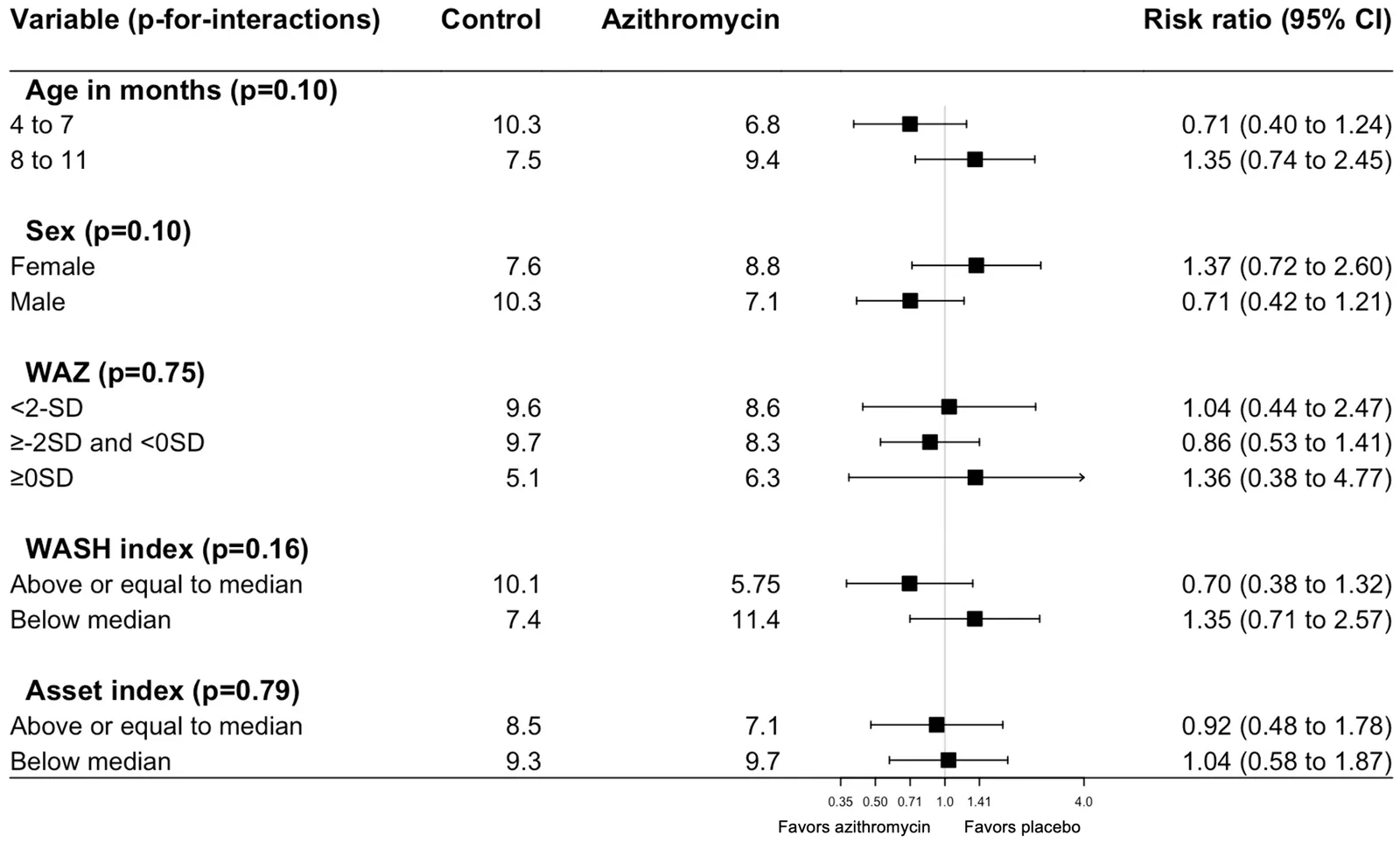
Prevalence of PCR-positive malaria (%) at day 14 for each effect modifier by treatment group. WAZ = weight-for-age Z-score. Predefined statistical significance at p-for-interactions = 0.1. Risk ratio estimates account for baseline values and are regression coefficients calculated with a mixed-effects regression model with random intercepts for clusters and accounting for interactions.

**Fig 3:**
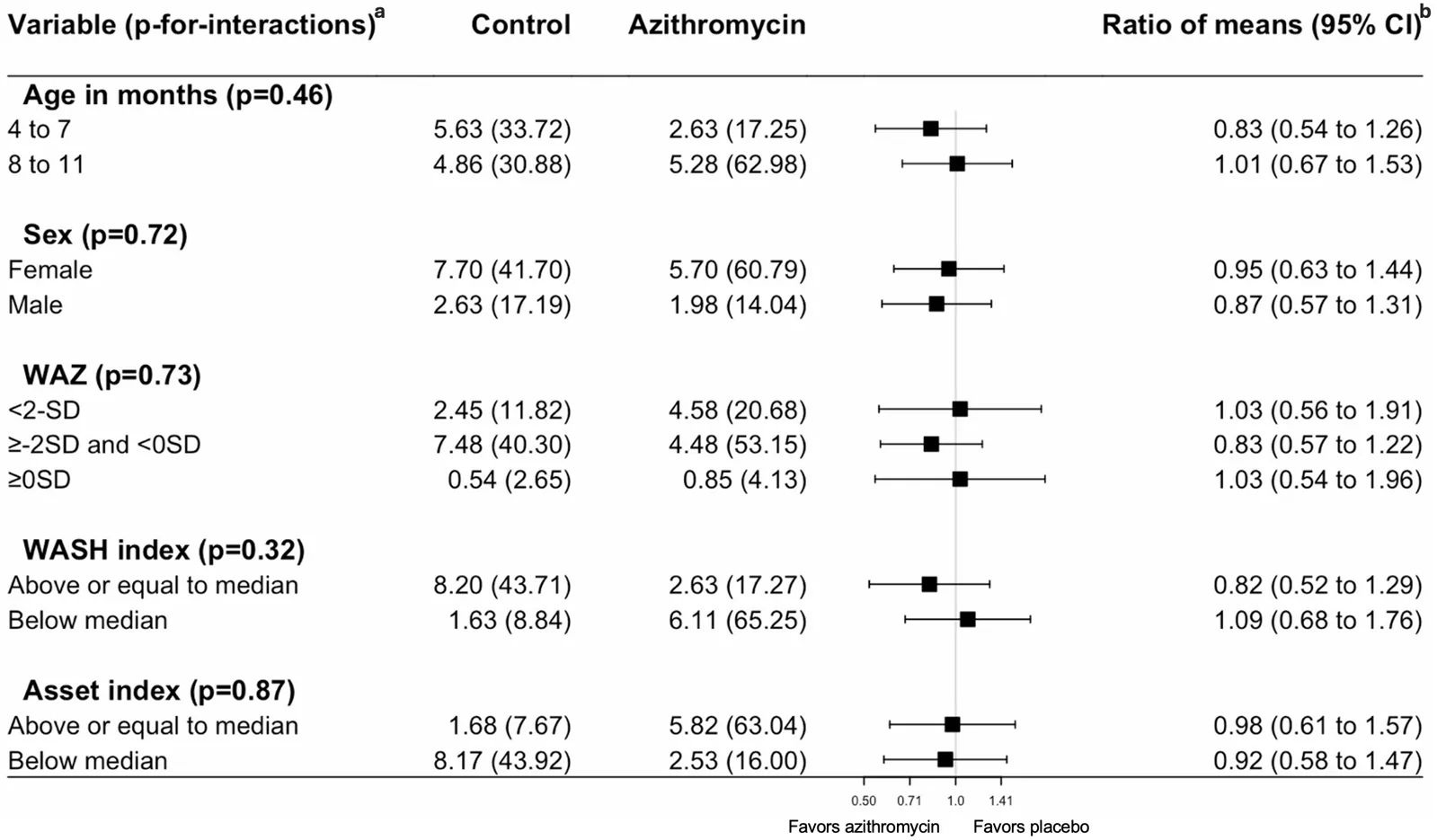
Parasite load (10^-3^ ng/μL) at day 14 for each effect modifier by treatment group. WAZ = weight-for-age Z-score. ^a^Predefined statistical significance at p-for-interactions = 0.1. ^b^Ratios of means are estimated ratios of geometric means due to the log-transformation of parasite load values. They account for baseline valuesand are regression coefficients calculated with a mixed-effects regression model with random intercepts for clusters and accounting for interactions.

The test for interactions was not significant for any of the tested potential effect modifier variables for malaria prevalence. No effect modifier subgroup was statistically significantly favoring either azithromycin or placebo for malaria prevalence or parasite load (Fig 2 and 3). Subgroup sample sizes were small, resulting in wide confidence intervals.

In a post-hoc analysis including only infants who were PCR-positive at baseline, mean parasite load at baseline was 0.0642 ng/μL (geometric mean 0.0035 ng/μL) in the control group and 0.0366 ng/μL (geometric mean 0.0066 ng/μL) in the azithromycin group. On day 14, mean parasite loads were 0.0510 ng/μL (geometric mean 0.0014 ng/μL) in the placebo group and 0.0363 ng/μL (geometric mean 0.0003 ng/μL) in the azithromycin group. Log-transformed, and accounting for baseline values and clusters, the ratio of means was 0.15 (95% CI: 0.06 to 0.37, p < 0.001) favoring azithromycin, indicating an 85% reduction in parasite load on average among malaria positive participants.

## Discussion

We aimed to test the effect of a single dose of azithromycin on malaria prevalence and parasite load among Malian 4–11-month-old infants. In a sample of 58 villages with 1158 infants, we did not find a statistically significant differences between azithromycin and control groups regarding prevalence of PCR-positive malaria, mean parasite load or hemoglobin levels.

These null results are likely because the study was underpowered, due to a lower-than-expected malaria prevalence and subsequently a smaller than expected number of malaria cases. At the time of the design of this LAKANA substudy, the 2018 Demographic Health Survey of Mali reported a 13% prevalence of malaria in Kayes region and 22% in neighboring Koulikoro region among 6–59 months old children [40]. In our sample, the baseline prevalence was clearly lower, at around 9%. Notably, all the point estimates in our study consistently favored azithromycin, including a 4% overall risk reduction, a 19% risk reduction among baseline malaria positive infants, a 9% reduction in overall parasite load, and an 85% reduction in parasite load among baseline PCR-positive infants. However, we were unable to reject the null hypotheses due to the low number of malaria cases. Common to many other studies not seeing a convincing effect on malaria is a smaller sample size (below 300 in follow-up) in both treatment groups [41–43].

Results from trials looking at the effects of azithromycin MDA on malaria vary widely. Many azithromycin trials have observed effects on malaria prevalence, parasite density, antibody response (force of infection), malaria clinic visits, and malaria mortality [6,20–23,44]. Some of the starkest reductions in malaria have been in a trachoma azithromycin MDA trial in Tanzania (73% reduction in malaria cases), the MORDOR trial in Niger (malaria parasitemia OR 0.54), and the CHAT trial in Burkina Faso (malaria mortality OR 0.67) [6,21,23]. Conversely, there are large-scale trials reporting no effect on malaria. A trial conducted in Mali and Burkina Faso testing SMC given together with placebo or azithromycin, observed more than 20 000 malaria cases, but reported no significant reduction in parasitologically confirmed malaria with azithromycin [24]. The MORDOR Tanzania and Malawi trials observed no significant reduction in malaria outcomes [26,43]. A recent large-scale trial in Burkina Faso (MIRAMA trial), with a similar design as LAKANA, treating only 1–11-month-olds only, reported no statistically significant reduction in malaria mortality in verbal autopsy analyses, but did not report parasitemia prevalence or parasite density [11]. Two trials in Burkina Faso, with similar timing design as our study (two weeks), tested malaria right before and two weeks after azithromycin distribution and reported no reduction in malaria [41,42].

Growing evidence points to a herd protection effect as a possible mechanism for azithromycin MDA mortality reduction [3,45,46]. Reduction in mortality has only been observed when all under 5-year-olds in the community have been treated [1–3,11,12]. One azithromycin trial in Niger demonstrated that treating only 1–4 year-olds resulted in a mortality reduction in untreated infants, suggesting a spillover protective effect [46]. Another azithromycin MDA study also reported possible herd protective spillover effects in untreated individuals [45]. Malaria is transmitted mostly from the immediate community reservoir, and if azithromycin has only a weak antimalarial effect, then treating only a small subset of the community should not result in notable herd protection against malaria. Because we treated only 4–11-month-old infants, any possible herd effects from azithromycin may have been missed. Similarly, the lack of a herd protection effect in the MIRAMA trial (also treating only infants), may explain the non-significant reduction in malaria deaths in their study [11].

Our study had several limitations. We only tested for *Plasmodium falciparum*, but this is unlikely to cause any major bias since *Plasmodium malariae, vivax* and *ovale* are very rare in Kayes region [47,48]. In the analysis of parasite load, malaria negative samples were assigned a value equal to half of the lowest serial dilution to avoid exclusion during log-transformation. While this approach is generally accepted in antibody titer studies, it may introduce a minor bias [49]. Although prespecified, the study was not powered for subgroup analyses; these were conducted to provide preliminary evidence on possible effect modifiers, requiring confirmation in larger studies. Our results cannot be generalized to settings with an ongoing rainy season malaria outbreak, extensive SMC coverage, or when older children are also treated.

In conclusion, this trial introduced new evidence on the effect of azithromycin on malaria in the WHO 2020 guidelines recommended infant subgroup. We observed a slightly lower malaria prevalence and parasite load with a single dose of azithromycin, but the results were not statistically significant, possibly due to a low number of malaria cases. The findings do not confirm, but are consistent with, the hypothesis that a single dose of azithromycin reduces malaria prevalence or parasite load among infants in this malaria-endemic West African setting.

## Supporting information

S1 CONSORT Checklist CONSORT (Consolidated Standards of Reporting Trials) checklist. (PDF)

S1 LAKANA Original Protocol LAKANA trial original protocol, 2019-11-27. (PDF)

S1 LAKANA Latest Protocol LAKANA trial protocol with latest amendments, 2024-11-13. (PDF)
