# Supplementary material for: A single dose of azithromycin versus placebo for malaria in infants: A cluster-randomized trial
Source: PLOS Glob Public Health. 2026 Aug 20;6(8):e0006518. doi: 10.1371/journal.pgph.0006518 (PMC13492813; doi:10.1371/journal.pgph.0006518)
Supplement: Supplementary file 3 [file pgph.0006518.s003.pdf]

LAKANA trial

A research plan for a cluster-randomized, double-blinded, parallel group, controlled trial, testing the effects of mass-drug administration of azithromycin on mortality and other outcomes among 1–11-month-old infants in rural Mali

Principal and co-principal investigators (in alphabetical order)

Per Ashorn, MD, PhD [1]

Ulla Ashorn, PhD [1]

Yin Bun Cheung, PhD [1,2]

Camilla Ducker, MBBS, MSc [3]

Nigel Klein, MBBS, PhD [4]

Samba O Sow, MD, MSc, FASTMH [5]

Participating Academic Institutions

[1]Center for Child Health Research, Tampere University, Faculty of Medicine and Health Technology, Tampere, Finland

[2]Centre for Quantitative Medicine, Duke-NUS Medical School, Singapore

[3]Tro Da Ltd, UK

[4]University College London, UK

[5]CVD-Mali, Bamako, Mali

Contact information: Per Ashorn, MD, PhD, Center for Child Health Research, Tampere University, Faculty of Medicine and Health Technology, Tampere Finland
Tel. +358 40 7280 345, E-mail: per.ashorn@tuni.fi

Protocol version 7.0: 13. November. 2024

Table of contents

1. PROTOCOL VERSION HISTORY **4**

2. EXECUTIVE SUMMARY **9**

2.01. ADMINISTRATIVE INFORMATION 9
2.02. KEY DETAILS OF THE TRIAL 10

3. BACKGROUND **12**

3.01. HEALTH PROBLEM TO BE ADDRESSED 12
3.02. STUDY OBJECTIVE 12
3.03. BACKGROUND TO AND JUSTIFICATION FOR THE STUDY 12
3.04. POTENTIAL PUBLIC HEALTH EFFECTS 13
3.05. DISTRIBUTION OF THE RESULTS 14

4. THE STUDY **14**

4.01. OBJECTIVES OF THE PROPOSED RESEARCH 14
4.02. DETAILED STUDY QUESTIONS 15
4.03. STUDY SITE AND TARGET POPULATION 17
4.04. STUDY DESIGN 17
4.05. ENROLLMENT CRITERIA FOR PARTICIPATING CLUSTERS AND PARTICIPANTS 18
4.06. TRIAL INTERVENTIONS AND CONTROL 19
4.07. OUTCOME VARIABLES 19
4.08. DATA COLLECTION SITES FOR VARIOUS OUTCOMES 22
4.09. SCHEDULE AND PROCEDURES FOR PARTICIPANT ENROLLMENT AND FOLLOW-UP 23
4.10. EXPECTED RECRUITMENT AND LOSS-TO-FOLLOW-UP RATES 25
4.11. PURCHASE, FORMULATION, STORAGE, AND DISTRIBUTION OF STUDY DRUGS 26
4.12. DATA COLLECTION ON INFANT AND CHILD MORTALITY 27
4.13. ADAPTIVE DESIGN: POSSIBLE CHANGES AFTER AN INTERIM ANALYSIS 27
4.14. DATA COLLECTION ON INFANT MORBIDITY 29
4.15. DATA COLLECTION ON INFANT GROWTH AND NUTRITIONAL STATUS 29
4.16. DATA COLLECTION ON ANTIMICROBIAL RESISTANCE 30
4.17. DATA COLLECTION ON MECHANISMS OF ACTION FOR AZITHROMYCIN 36
4.18. STAKEHOLDER PERSPECTIVES ON FEASIBILITY, INCLUDING ECONOMIC ANALYSES 41
4.19. STRATEGIE AVANCÉE AND FIELD WORKERS' ASSESSMENT OF HARD-TO-REACH AREAS 45
4.20. DATA COLLECTION ON TRIAL SAFETY 46
4.21. GROUP ALLOCATION AND TRIAL CODE MANAGAMENT 46
4.22. METHODS FOR PROTECTING AGAINST OTHER SOURCES OF BIAS 47
4.23. WITHDRAWAL FROM PARTICIPATION AFTER ENROLLMENT 48
4.24. OTHER TREATMENTS TO TRIAL PARTICIPANTS 48
4.25. CO-ENROLLMENT GUIDELINES 48
4.26. STORAGE OF BIOLOGICAL SAMPLES 48
4.27. DATA RECORDING AND MANAGEMENT 49
4.28. PILOT STUDY 50
4.29. QUALITY ASSURANCE AND CONTROL 50

4.30. ORIGINALLY PLANNED TIME SCHEDULE 50

5. STATISTICAL CONSIDERATIONS AND DATA ANALYSIS 52

5.01. SPECIFIC HYPOTHESES 52
5.02. ANALYTICAL APPROACH 53
5.03. FREQUENCY OF ANALYSES 55
5.04. SAMPLE SIZE AND ITS JUSTIFICATION 56
5.05. SUBGROUP ANALYSES 57

6. ETHICAL CONSIDERATIONS 58

6.01. GENERAL PRINCIPLES 58
6.02. INFORMED CONSENT 58
6.03. POSSIBLE RISKS AND BENEFITS TO STUDY PARTICIPANTS 59
6.04. AMOUNT OF COLLECTED BLOOD AND OTHER BIOSPECIMENS 60
6.05. INSURANCE COVERAGE 61
6.06. COMPENSATION TO STUDY PARTICIPANTS 61
6.07. PAYMENTS TO THE TRIAL ORGANISERS 61
6.08. DATA SAFETY AND MONITORING BOARD (DSMB) 62
6.09. STOPPING RULES FOR THE STUDY 62

7. PERSONNEL, RESOURCES, AND STUDY MANAGEMENT 63

7.01. RESEARCH INSTITUTIONS 63
7.02. PRINCIPAL INVESTIGATORS 63
7.03. STUDY STATISTICIANS 65
7.04. OTHER MAIN SCIENTISTS AND THEIR RESPONSIBILITIES 65
7.05. OTHER EMPLOYED STAFF 65
7.06. ARRANGEMENTS FOR DAY-TO-DAY MANAGEMENT OF THE STUDY 66
7.07. COMMUNICATION PLAN 67
7.08. PROJECT STEERING GROUP 67
7.09. NATIONAL AND INTERNATIONAL ADVISORY GROUPS 68

8. TRAINING PROVIDED 68

9. POSSIBLE CONSTRAINTS 68

10. FUNDING 69

11. REFERENCES 70

12. APPENDICES 77

APPENDIX 1A: INFORMATION ABOUT THE TRIAL TO VILLAGE LEADERSHIP 77
APPENDIX 1B: VERBAL CONSENT FORM FOR VILLAGE PARTICIPATION 83
APPENDIX 2A: INFORMATION ABOUT THE TRIAL TO A POTENTIAL PARTICIPANT 84
APPENDIX 2B: VERBAL HOUSEHOLD CONSENT FORM FOR TRIAL PARTICIPATION 90
APPENDIX 3: CONSENT TO PROVIDE STUDY MEDICINE TO AN INDIVIDUAL INFANT 91
APPENDIX 4A: INFORMATION ON SECONDARY OUTCOME DATA COLLECTION 93
APPENDIX 4B: CONSENT FORM FOR PARTICIPATION IN LAKANA SUB-STUDIES 98
APPENDIX 5, REFERRAL LETTER FOR ASYMPTOMATIC CHILDREN WITH ABNORMAL LABORATORY VALUES OBSERVED DURING A VILLAGE VISIT 102

1. Protocol version history

AMENDMENT NO.	PROTOCOL VERSION #	DATE ISSUED	AUTHOR/S OF CHANGES	DETAILS OF CHANGES MADE	SUBMITTED TO IRB, **MALI** (DATE)	APPROVED AT IRB , **MALI** (DATE/NO)	SUBMITTED TO IRB , **TAU** (DATE)	APPROVED AT IRB, **TAU** (DATE/NO)
	1.0	27.11.2019	Ashorn	First complete protocol draft. Submitted for IRB review but withdrawn by the research team for further edits	29.11.2019	Withdrawn	N / A	N / A
1	2.0	17.12.2019	Ashorn	Expanded the trial area: Earlier only Kayes region, now parts of Kayes, Kita, and Koulikoro regions.<br>Removed the bridging trial aftern interim analysis<br>Cut out the last 6 calendar months of follow-up in the trial. Half of the clusters now followed up for 8 MDA rounds, 30% for 7 MDA rounds and 20% for 6 MDA rounds<br>Increasing sample size from 650 to 750 mortality analysis clusters<br>Changed the ratio of control vs azi-biannual vs azi quarterly clusters from 1 : 1 : 1 to 3 : 4 : 2<br>Reduced the number of interim analyses from 2 to 1 – to be done at approximately 60% of the data accrual<br>Changed the timing of AMR and other biological sample collection slightly<br>Added a second field laboratory to Koulikoro region<br>Expanded the area of a possible MDA roll-out area to 3-5 regions, including the entire Kayes, Kita and Koulikoro, and possibly parts of Bougouni and Segou.	17.12.2019	27.12.2019	13.01.2020	04.02.2020
2	3.0	08.03.2022	Luoma, Adubra, Ashorn	Increased sample size from 830 to 1150 clusters (or 35,650 infants treated on MDA1, whichever comes first), because of lower-than-expected numbers of infants / cluster and baseline mortality. Edited assumptions behind the sample size selection.<br>Changed the plan of primary analysis, to include mortality data also from the secondary outcome sample. Clarified the process of code breaking before primary analysis	15.3.2022	28.3.2022		

AMENDMENT NO.	PROTOCOL VERSION #	DATE ISSUED	AUTHOR/S OF CHANGES	DETAILS OF CHANGES MADE	SUBMITTED TO IRB, **MALI** (DATE)	APPROVED AT IRB , **MALI** (DATE/NO)	SUBMITTED TO IRB , **TAU** (DATE)	APPROVED AT IRB, **TAU** (DATE/NO)
				Extended the duration of cluster enrolment from 52 to 78 weeks<br>Removed the inclusion of a tertiary AMR sample<br>Changed the timing for AMR analyses to include samples at baseline, and at 12, 24, ad 36 months after baseline<br>Clarified the AMR analyses to include resistance to WHO "Access" group antibiotics among azithromycin-resistant bacterial isolates<br>Edited laboratory tests to be done in the mechanistic study: replaced fecal calprotectin measurement with three other fecal biomarkers: neopterin, myeloperoxidase, and alpha 1 antitrypsin<br>Provided more detail on the age of infants eligible for the intervention (aged expressed in days, not only months)<br>Provided further details on possible outcome scenarios for the planned interim analysis<br>Change establishment of a second field site for AMR testing in Koulikoro as optional.<br>Edited participant information document with the updated information on sample size and age of eligible infants<br>Provided detailed instructions regarding study drug administration and referral of children participating in mechanistic substudy for whom blood samples are collected and Hb and CRP levels are measured on site. Added a document that will be used to advice CSCOM personnel on participant management (Appendix 5).				
3	4.0	27.06.2022	Luoma, Adubra, Ashorn	Added a tertiary sample for collection of AMR and mechanistic study samples in a subset of villages closer to Bamako, i.e., in the study regions of Koulikoro and Kita.<br>Replaced Norgen Stool Nucleic Acid kit by DESS media (DMSO/EDTA/saturated sodium chloride) for the preservation of second rectal swab collected at AMR study visits.<br>Specified that AMR and mechanistic study samples may be directly transported to CVD-Mali Bamako laboratory for processing.				

AMENDMENT NO.	PROTOCOL VERSION #	DATE ISSUED	AUTHOR/S OF CHANGES	DETAILS OF CHANGES MADE	SUBMITTED TO IRB, **MALI** (DATE)	APPROVED AT IRB , **MALI** (DATE/NO)	SUBMITTED TO IRB , **TAU** (DATE)	APPROVED AT IRB, **TAU** (DATE/NO)
				Changed the timing for mechanistic study data and sample collection to MDA 4 for 4–11-month-old infants, MDA 6-8 and visit 9 for 6-8- and 12-14-months old infants and children.<br>Edited one hypothesis for the mechanistic study extending the assessment of community-level prevalence of malaria parasitaemia at the end of the intervention (9th visit) to 12-14 months old children.<br>Edited instructions regarding referral of children who have abnormal Hb or CRP values in the mechanistic sub-study. Edited the referral document (Appendix 5).<br>Specified that anthropometric measurements for growth study, and samples collection for mechanistic study will be conducted at the time of the study visits, instead of two weeks later, and possibly in a central location in village.<br>Removed head circumference from growth study outcomes.<br>Changed the target age for morbidity data collection to 4-14 months.<br>Provided further details on how the feasibility study will be done. Added an economic analysis (chapter 4.18).<br>Clarified, based on trial adaptive design, the interim analysis outcomes that will lead trial conduct to be altered.<br>Specified that a 1:1 allocation ratio will be used to re-randomize previous placebo-clusters in the two AZI groups if after interim analysis the placebo arm is dropped.<br>Clarified that random allocation of letter codes to treatment was conducted by the external partner, RTI International, who then provided the code to Pfizer.<br>Clarified that closed-testing procedure will be used to adjust statistical analyses for multiple comparisons.<br>Clarified that analytical method for mechanism study will account for clustering.				

AMENDMENT NO.	PROTOCOL VERSION #	DATE ISSUED	AUTHOR/S OF CHANGES	DETAILS OF CHANGES MADE	SUBMITTED TO IRB, **MALI** (DATE)	APPROVED AT IRB , **MALI** (DATE/NO)	SUBMITTED TO IRB , **TAU** (DATE)	APPROVED AT IRB, **TAU** (DATE/NO)
4	5.0	23.03.2023	Luoma, Adubra, Ashorn	Specified that mechanistic study in tertiary sample will be conducted during rainy season (Peak malaria transmission).<br>Specified that Growth study may be conducted in tertiary sample if planned sample size for the study is not achieved with secondary sample.<br>Specified “Parasite density” as an outcome of mechanistic study to clarify the quantitative aspect of parasitaemia measures besides malaria prevalence.<br>Provided clarification on exploratory analysis and effect modifiers to be tested.<br>Edited one hypothesis for the mechanistic study extending the assessment of community-level prevalence of malaria parasitaemia at the end of the intervention (9th visit) to 1-59 months old children. And, provided further details on sample size and sampling methods, as well as compensation.<br>Edited the Information about Participation in the AMR and other sub-studies to include Kati as one of the CSComs included in the sub studies (tertiary sample).				
5	6.0	15.12.2023	Alber, Fan, Klein	Expanded the age group of children being sampled for AMR analysis at visit 11 to also include 15-26 months old children. Expanding the age group at visit 11 will allow to assess whether carriage of antibiotic resistant bacteria persists in individual children who received 1-4 doses of azithromycin MDA one year prior to sampling and who live in a village that have stopped MDA intervention.<br><br>Additionally, we will sample 24-35 months old children at visit 9 in the Kati region (tertiary AMR sample). This will determine whether carriage of antibiotic resistant bacteria persist in children who received 1-4 doses of azithromycin MDA one year prior to sampling but live in a village where MDA intervention has continued.				

AMENDMENT NO.	PROTOCOL VERSION #	DATE ISSUED	AUTHOR/S OF CHANGES	DETAILS OF CHANGES MADE	SUBMITTED TO IRB, **MALI** (DATE)	APPROVED AT IRB , **MALI** (DATE/NO)	SUBMITTED TO IRB , **TAU** (DATE)	APPROVED AT IRB, **TAU** (DATE/NO)
6	7	13.11.2024	Luoma, Ashorn	Added “hard-to-reach” village status and Strategie Avancée as one of the potential effect modifiers in sections 4 and 5.<br><br>Included a survey among the field workers as a part of the feasibility aspect.<br><br>Amended a study question regarding hardness-to-reach and the inclusion of village to Strategie Avancée being a predictor of high mortality (section 4.02.)<br><br>Amended the aspect of field worker assessment to section 4.18.				

1

2. Executive summary

2.01. Administrative information

Trial name:	LAKANA. A cluster-randomized, double-blinded, parallel group, controlled trial, testing the effects of mass-drug administration of azithromycin on mortality and other outcomes among 1–11-month-old infants in rural Mali
Sponsor:	Tampere University, FIN-33014 Tampere, Finland
Funding:	The Bill & Melinda Gates Foundation
Monitor:	Likak Research Ltd, Senegal
Data Safety and Monitoring Board:	Dr. Robert Black, JHU, chair (Global child health)<br>Dr. Julia Bielicki, UKBB, Basel, Switzerland (AMR)<br>Dr. Alassane Dicko, USTTB, Bamako, Mali (Epidemiology)<br>Dr. Queen Dube, COM, Malawi (Child health policy)<br>Dr. Paul Milligan, LSHTM, London, UK (Biostatistics)
Principal Investigator	Dr. Per Ashorn, MD, PhD
Co- Principal Investigators:	Dr. Samba Sow, MD, MSc, FASTMH<br>Dr. Ulla Ashorn, PhD<br>Dr. Nigel Klein, MBBS, PhD<br>Dr. Camilla Ducker, MBBS, MSc<br>Dr Yin Bun Cheung, PhD
Participating institutions:	Tampere University, Finland<br>CVD-Mali, Bamako, Mali<br>University College London, UK<br>Tro Da Ltd, UK<br>Centre for Quantitative Medicine, Duke-NUS Medical School, Singapore
Trial site:	Kayes, Kita, and Koulikoro regions, Mali
Laboratory analyses:	All participating institutions
Contact details:	Dr. Per Ashorn, MD, PhD<br>Professor of Paediatrics, Center for Child Health Research, Faculty of Medicine and Health Technology<br>Tampere University, FIN-33014 Tampere, Finland<br>Tel: +358 40 7280 354, Email: per.ashorn@tuni.fi

2.02. Key details of the trial

**Background:**	There is recent evidence that mass-drug administration (MDA) of azithromycin to apparently healthy young children, reduces their mortality in some, but not in all low-income African settings.
**Main objective:**	To determine the impact of quarterly (given every 3 months) or bi-annual (given twice / year, between January and June) azithromycin MDA to 1–11-month (29-364 days) old infants on their mortality and other health outcomes, when provided in a context of a seasonal malaria chemoprevention (SMC) program.
**Trial approach:**	A cluster-randomized, placebo-controlled, double-blinded, parallel-group, three-arm clinical trial with adaptive design, in rural and peri-urban villages in the Kayes, Kita, and Koulikoro regions of Mali, Sub-Saharan Africa.
**Main methods:**	Selected and consenting villages will be randomly allocated into three groups: Control, Azi-quarterly, and Azi-biannual. Households in the participating villages will be visited at quarterly intervals by study staff. At each visit, there will be a census of all household members as well as recording of births and child deaths since the last visit. All 1-11 months old infants (age 29-364 days), for whom there is a consent from a guardian for study drug provision, will be weighed and given a single dose of study drug. Infants in control group will receive placebo at each visit, infants in Azi-bianual group will receive placebo at visits between July and December and 20 mg / kg azithromycin at visits between January and June, and infants in the Azi-quarterly group will receive 20 mg / kg azithromycin at each visit. The village visit will be repeated every 3 months for 8 times for study drug administration and one more time for census and vital statistics recording. SMC with amodiaquine and sulfadoxine-pyrimethamine will be offered through the national health service to all 3–59-month-old children on a monthly basis between July and October in each year.
**Primary outcome**	Mortality (deaths / 1000 years at risk) among children who were 1-11 month (29-364 days) old at mass azithromycin administration.
**Other outcomes**	Episodes of ARI, malaria, and diarrhea<br>Infant and young child growth<br>Prevalence of antimicrobial resistance towards azithromycin<br>Malaria parasitemia, inflammation, and immune development<br>Feasibility of azithromycin MDA implementation including economic analysis<br>Incidence of serious adverse events (SAE) and adverse events (AE)<br>Mortality among children, who were 12–59-month-old at the MDA

Adaptive design: One interim analysis will be completed when approximately 60% of the 3-month follow-up intervals have been completed. If there is a statistically significant mortality difference between the quarterly azithromycin group and the placebo group as well as between the biannual azithromycin group and the placebo group but not between the two azithromycin groups, the placebo arm will be discontinued, and trial completed with two arms only. If there is also a statistically significant difference also between the two azithromycin groups, the trial will be stopped, and the team will offer to work with Malian Ministry of Health to provide azithromycin MDA in the trial site and elsewhere in the regions of Mali.

Sample size: Approximately 1150 clusters (villages). At each MDA round, each cluster is expected to contain on average 31 eligible infants who will provide data to the study.

Stored samples: Some vials of frozen plasma, white blood cells, urine and stools and dried blood spots from a sub-group will be stored for later analyses.

3. Background

3.01. Health problem to be addressed

Infant and child mortality in Mali and elsewhere in sub-Saharan Africa.

According to the most recent estimates, 3.0 million infants and children between 1-59 months die each year, mostly from infectious diseases (WHO, 2018). Approximately 1.2 million of these deaths are estimated to take place in Western and Central Africa, 0.7 million in Eastern and Southern Africa, 0.7 million in in South Asia, 0.3 million in East Asia and Pacific, and 0.1 million elsewhere (UNICEF, 2017). By 2030, global leaders have committed to ending preventable deaths of children under 5 years of age, with all countries aiming to reduce under-5 mortality to at least as low as 25 per 1,000 live births (United Nations Development Program, 2014).

3.02. Study objective

To determine the impact of quarterly and bi-annual mass azithromycin administration to 1-11 month (29-364 days) old infants in rural and periurban Mali on their mortality and other health outcomes, on mortality of older children, and to assess the feasibility and possible mechanisms of action of the intervention.

3.03. Background to and justification for the study

Azithromycin mass drug administration (MDA) has been used for approximately 20 years to control trachoma, a blinding eye disease. Over 600 million doses of oral azithromycin have been prescribed to children and adults living in an areas of active trachoma program (Emerson et al., 2017). In some settings, trachoma control programs with repeated treatment of all community members with azithromycin has been associated with prevention of other infections, such as malaria, diarrhea and pneumonia (Whitty et al., 1999; Fry et al., 2002; Coles et al., 2011; Coles et al., 2012; Gaynor et al., 2014; Schachterle et al., 2014). Two studies in the same community in Ethiopia, one with a case-control design and the other one being a cluster-randomized trial suggested that azithromycin MDA might also be associated with reduced under-five mortality (Porco et al., 2009; Keenan et al., 2011)

Encouraged by the previous results, two randomized controlled trials were implemented recently to test the hypothesis that azithromycin MDA would be associated with reduced mortality among 1–59-month-old children in Sub-Saharan Africa. The first study (MORDOR) was a placebo-controlled trial in Malawi, Tanzania, and Niger. Approximately 1500 clusters were randomly allocated into being either an intervention or a control cluster. In the intervention clusters, all 1–59-month-old children were treated with a single dose of azithromycin every 6 months for two years; in control clusters

children received a respective placebo dose. Neonates, older children and adults were not treated.

In the MORDOR sample, azithromycin treatment was associated with a statistically significant 13.5% overall reduction in mortality among 1–59-month-old children. The difference between intervention and control was approximately 18% in Niger, 6% in Malawi and 3% in Tanzania. The differences were largest among 1–5-month-old children and after the fourth treatment cycle – but there was not enough power to formally test hypotheses about effect modification. Verbal autopsy data suggest that 41% of the deaths were due to malaria, 18% to diarrhea or dysentery and 12% pneumonia (Keenan et al., 2018).

In another trial in Burkina Faso and Mali, researchers tested the impact of combining azithromycin MDA to seasonal malaria chemoprevention (SMC, with sulfadoxine-pyrimethamine and amodiaquine). The intervention was offered monthly to 3–59-month-old children over three consecutive months. In this sample, there was no difference between the trial arms in a combined outcome of hospitalization or death (Chandramohan et al., 2019).

Based on earlier results, it thus seems that azithromycin MDA reduces infant or child mortality in some, but not in all low-income African settings. An expert committee for the World Health Organization has reviewed the data and called for further research, to strengthen the evidence-based on azithromycin MDA. Because of the observed heterogeneity and possible effect modification by SMC or other co-interventions, further trials in new settings are needed in order to make evidence-based public health recommendations about the use of this treatment. Because of a fear for antimicrobial resistance (AMR), there is also a need to investigate if a more limited target group (1-11 months) would alleviate the AMR risk but retain the intervention's impact on mortality. Because the MORDOR data suggested that the mortality benefit is concentrated to the first three months after the MDA dose (Porco et al., 2019), there is also a need for testing the impact of more frequent MDA dosing, on mortality, AMR, and feasibility of the intervention, as compared to the previously tested bi-annual treatment of 1–59-month-old children. Because of the earlier results suggesting limited additional benefit from azithromycin when provided concomitantly with SMC, there is also a need to test the impact of bi-annual dosing that is provided quarterly, but only during seasons when SMC is not offered.

LAKANA trial has been designed to address the health impacts of quarterly and biannual azithromycin MDA when delivered to 1-11-month-old infants in a high-mortality setting where malaria is holoendemic but there is also a functioning SMC program in place. The trial name (LAKANA) is an abbreviation from "Large-scale Assessment of the Key health-promoting Activities of two New mass drug administration regimens with Azithromycin. In Bambara, which is the local language in the study area, LAKANA means "*to protect*" or "*to be safe*".

3.04. Potential public health effects

The present study may offer a partial solution to a major public health problem in Mali and elsewhere in Sub-Saharan Africa. Should the intervention work, it will significantly contribute to the options for successfully reducing child mortality which is one of the United Nations sustainable development goals. Thus, if the trial provides supportive evidence for a positive mortality benefit by azithromycin MDA, it will form the first phase of a national public health program in Mali. In phase 2, that would still form a part of the currently funded project, an azithromycin MDA program would be rolled out in the regions where the trial is implemented, if the government of Mali considers that desirable. In phase 3, there could be a national roll-out, the details of and funding for which would need to be separately negotiated.

Large-scale use of antibiotics raises always a concern of antimicrobial resistance, which could negatively impact clinical outcomes in the communities in which resistance emerges. Multiple studies have documented relatively rapid emergence of macrolide resistance following mass drug administration with azithromycin (Rogawski et al., 2015) but resistance appears to decline quickly after discontinuation of treatment (Sánchez et al. 1994; Mack et al., 2019) and there is little evidence of AMR spread to individuals who have not received the antibiotic (Julia Bielecki, personal communication). Because of the concern of possible AMR, we will monitor its prevalence closely in this trial, before, during and after the intervention and both among those receiving and those not received MDA with the study drugs.

3.05. Distribution of the results

The results will be distributed and discussed with the local community, Malian health researchers, regional and national level representatives of the Ministry of Health in Mali and the World Health Organization. Main findings will be published in international peer-reviewed journals.

The study material will also be used for post-graduate training of Malian and Finnish students.

4. The study

4.01. Objectives of the proposed research

Primary objective:

1. To evaluate the impact of two azithromycin MDA regimens on infant mortality and other health outcomes, when provided in a context a rural West-African high-mortality context with an ongoing seasonal malaria chemoprevention (SMC) program.

Secondary objectives:

2. To evaluate the effect of alternative MDA frequencies on AMR and host microbiota composition
3. To investigate the feasibility of alternative azithromycin MDA strategies, including economic analysis
4. To test hypotheses that azithromycin MDA eliminates malaria parasitemia and reduces general and intestinal inflammation in asymptomatic children and to collect and store biological samples for assessing other possible mechanisms of azithromycin effect

4.02. Detailed study questions

Detailed questions on mortality (primary objective):

1.1. Does biannual azithromycin MDA to 1-11 month (29-364 days) old infants, when provided quarterly during non-malaria season, reduce their mortality in a Mali-like setting, in which there is a functional SMC program

1.2. Does quarterly azithromycin MDA, provided throughout the year (i.e., 4 doses / year) to 1–11-month-old infants reduce their mortality in a Mali-like setting?

1.3. Does quarterly MDA have a bigger mortality effect than biannual MDA?

1.4. Do the following factors modify the effect of azithromycin MDA on mortality? (Exploratory analysis)

a. Infant age at the time of MDA (1-5 months vs 6-11 months)
b. Infant weight-for-age, length-for-age, weight-for-length at the time of MDA
c. Infant sex
d. Season of MDA dosing (malaria versus non malaria season) and SMC implementation in village (SMC given versus SMC not given)
e. Cluster level coverage of SMC
f. Cluster level baseline mortality (established at first census)
g. Cluster and individual level coverage and number of administered azithromycin MDA doses
h. District of residence
i. Distance from the nearest health facility
j. Household asset or income index
k. Household WASH index
l. National outreach strategy category (standard/advanced)
    i. There is an existing national outreach strategy (*Strategie Avancée*) in place to target communities that have limited access to healthcare. Inclusion into that strategy will be treated as a potential effect modifier.
m. Hard-to-reach village status assessed by the field workers
    i. In addition to objective measures (geographical location distance to health facilities) and existing national assessment (Strategie Avancée), we will conduct a survey among field workers to obtain a subjective assessment on how difficult the village is to reach. The results will be summarized to a composite variable that will be used as a dichotomous variable on whether or not the village is "hard-to-reach".

1.5.Does biannual or quarterly azithromycin MDA to 1–11-month-old infants reduce mortality among 12–59-month-old children living in the same communities (exploratory analysis)
1.6.Are hard-to-reach communities more likely to experience higher mortality than those within closer proximity or easier access to urban locations and health facilities (exploratory analysis)
1.7.Are communities under Strategie Avancée more likely to experience higher mortality than those in standard national outreach category (exploratory analysis)

Detailed questions on other health outcomes (primary objective):
1.8.Does biannual or quarterly azithromycin MDA to 1–11-month-old infants reduce the prevalence of ARI, diarrhea, or malaria symptoms among them?
1.9.Does biannual or quarterly azithromycin MDA to 1–11-month-old infants improve their weight gain, linear growth (length), or nutritional status (assess by Mid upper-arm circumference MUAC, weight-for-height-Z-score WHZ, Length-for-age Z-score LAZ, Weight-for-age Z-score WAZ)?

Detailed questions on AMR (secondary objective):
2.1.Does biannual or quarterly azithromycin MDA to 1–11-month-old infants cause an increase in the prevalence of phenotypic azithromycin resistance among *S. pneumoniae,* or *E. coli* strains isolated from 4-14-month of infants one year after the intervention has stopped?
2.2.What is the impact of biannual or quarterly azithromycin MDA to 1–11-month-old infants on the prevalence of azithromycin resistance among *S. pneumoniae* or *E. coli* strains isolated from 49–59-month-old children, who live in the same Malian communities but have not received azithromycin MDA, during and after the intervention period?
2.3.Does azithromycin resistant bacteria persist in individual children who received 1-4 doses of azithromycin 1 year after azithromycin MDA has stopped?
2.4.Does azithromycin resistant bacteria persist in individual children who received 1-4 doses of azithromycin 1year after their azithromycin MDA has stopped but village MDA intervention has continued?
2.5.What is the impact of biannual or quarterly azithromycin MDA to 1–11-month-old infants on the diversity and composition of their intestinal microbiota and prevalence of azithromycin resistance genes in intestinal bacteria?

Detailed questions on feasibility including economic analysis (secondary objective):
3.1 Is scaling-up of azithromycin MDA as a national program to reduce mortality among 1–11-month-old infants feasible in Mali according to stakeholders?
3.2 What are the factors that need to be considered for various scaling-up strategies?
3.3 What is the level of feasibility, inter-rate reliability, and validity of an instrument targeted to capture a subjective assessment of hardness-to-reach amongst the field workers?
3.4 Is there an association between hard-to-reach village status and advanced national outreach strategy status (exploratory analysis)

Detailed questions on mechanisms of azithromycin action (secondary objective):

4.1 Does azithromycin MDA reduce malaria prevalence and parasite density, and increase blood hemoglobin concentration among 1–11-month-old infants?
4.2 Does azithromycin MDA reduce general inflammation (plasma C-reactive protein concentration) among 1–11-month-old infants?
4.3 Does azithromycin MDA reduce intestinal inflammation (concentration of Alpha-1-antitrypsine, neopterin, and myeloperoxidase from stool sample) among 1–11-month-old infants?

4.03. Study site and target population

The project will be carried out in rural and peri-urban villages in the Kayes, Kita, and Koulikoro regions of Mali, in Sub-Saharan Africa. The regions are situated in western Mali, bordered by other Malian regions, Mauritania, Senegal and Guinea. The region has 235 municipalities (approx. 90% rural), containing close to 3,000 villages. Main means of subsistence are farming (cattle and arable), fishing, crop-picking, cotton, peanut and oil production. The regions also have goldmining activities (in both formal and informal settings). Spoken local languages include Bambara, Malinké, Sarakolé / Soninké / Marka, Kassoké, and Peulh.

While the exact mortality rates in the study area are unknown, under-five mortality rate (U5MR) is approximately 100 / 1000, infant mortality rate (IMR) approximately 60 / 1000 and neonatal mortality rate (NMR) approximately 35 / 1000. These figures are based on regional 2012-2013 DHS data, when U5MR in Kayes was 96, IMR 60, and NMR 34. There was a newer DHS completed in Mali in 2018 and the currently available statistics indicate that child mortality has remained at the 2012-2013 levels – hence we assume that this is the case also in Kayes, Kita, and Koulikoro.

According to the DHS 2018 report, 19% of U5 children in Kayes are underweight, 26% stunted and 9% have severe acute malnutrition. The prevalence of malaria in children aged 6-59 months was 12.6%, this prevalence being much higher in rural areas (23%) than in urban areas (2%). Seasonal Malaria control is delivered monthly to children aged 3-59 months between July and October.

The LAKANA trial will be implemented in 7-10 health districts in Kayes, Kita and Koulikoro. In the defined study area, there will approximately 1.5 million inhabitants, of whom 300,000 are under-five-years old and 60,000 under-one-year old.

4.04. Study design

A cluster-randomized, placebo-controlled, double-blinded, parallel-group, three-arm clinical trial, with adaptive design.

4.05. Enrollment criteria for participating clusters and participants

The units of intervention allocation, enrollment to the trial, treatment with study drugs, and primary outcome measurement will be different, as explained in detail below:

1. The unit of intervention allocation (cluster) will be village (or part of a village). Thus, in any one village, all infants will receive the same treatment at each MDA round.

2. The unit of enrollment will be a household representative (typically the head of household or her / his deputy). An enrolled participant's household will be visited regularly over a period of two years. During the visits, selected household members will be interviewed about household composition and eligible infants are offered MDA with the study drugs. In a sub-sample of villages, some household members may be invited to enrol in sub-studies and additional data collection.

3. The unit of treatment with study drugs will be an infant. At each home visit, the research staff will assess the eligibility of each child in the household to receive study drugs. In consequtive visits, some of the children will be the same who have been treated already earlier, whereas others will be new – either because they were newly born, they have recetly migrated to the area, or they were not available for treatment at the earlier MDA rounds.

4. The unit of primary outcome measurement will be a 3-month time interval, starting from the previous home visit and ending with the current one. Any one child may contribute 1-4 time-intervals to the main outcome analysis.

Eligibility and permission for participation and treatment will be determined on three levels: cluster (village), household, and child. On the cluster level, leaders of eligible villages will be asked a permission for study activities to take place in their areas. On a household level, a household representative will be asked a consent for trial participation, including periodic household visits by the study team and MDA to eligible infants. On the child level, eligibility for receiving study drugs will be checked at each of the MDA visits, i.e. max 8 times during the trial.

On a cluster level, the inclusion criteria will be:
1. Location within Kayes, Kita, or Koulikoro region
2. Considered accessible and safe by the local health authorities and research team
3. Considered non-urban by the local health authorities and research team
4. Permission from community leadership

On a household level, the inclusion criteria (for trial enrolment) will be
1. Location within a cluster that is included in the study
2. Verbal consent from a head of household or an adult authorized by her / him

On a child level, the criteria for receiving study medication will be:
1. Residence in a household enrolled in the trial
2. Age between 29 and 364 days

3. Verbal consent from at least one caregiver

On a child level, the exclusion criteria (for not receiving study medication) will be:
1. Weight below 3.0 kg (3rd centile for healthy 1 month old infants in the WHO growth charts)
2. Known allergy to macrolides, as judged by a caregiver report of the infant experiencing an adverse reaction after oral ingestion of medication, that was deemed likely to be a macrolide by the interviewing data collector.

4.06. Trial interventions and control

Participating villages will be randomly allocated to three different intervention groups in a ratio of 3 : 2 : 4 (control : azi-quarterly : azi-biannual). Within each village, consenting households will be visited quarterly (at 3-month intervals), for nine times. At the first eight of these visits, 1–11-month-old eligible infants (age 29-364 days), for whom there is a consent for study drug provision, will be weighed and given a single dose of study drug (azithromycin mixture or respective placebo mixture).

All study drugs will be coded, so neither the study staff, nor the participants will know who gets what. In practice, children will receive the following:

1. Control villages: Placebo mixture every three months

2. Azi-quarterly Azithromycin mixture every three months

3. Azi-biannual Azithromycin mixture at quarterly visits between January and June, Placebo mixture at quarterly visits between July and December

The dose of the study drug will be 20 mg (0.5 ml) / kg child weight, or an equal volume of respective placebo mixture. The study drugs will be given as a single oral dose under direct observation, by community volunteers who are involved in other national mass drug administration campaigns in the same villages.

Figure 1 shows a schematic illustration of ingredients in quarterly study drugs, in the three study groups:

	Apr-Jun 2020	Jul-Sep 2020	Oct-Dec 2020	Jan-Mar 2021	Apr-Jun 2021	Jul-Sep 2021	Oct-Dec 2021	Jan-Mar 2022	Apr-Jun 2022	Jul-Sep 2022	Oct-Dec 2022
Control	P	P	P	P	P	P	P	P	P	P	P
Azi-quarterly	A	A	A	A	A	A	A	A	A	A	A
Azi-biannual	A	P	P	A	A	P	P	A	A	P	P

A = Azithromycin, P = placebo

4.07. Outcome variables

Primary efficacy outcome:
1. Mortality (deaths / 1000 years at risk) among children who were 1-11 month (29-364 days) old at the time of study drug administration.

2. As an exploratory analysis, we will assess the following factors as potential effect modifiers to the azithromycin impact on mortality among the 1–11-month (29-364 days) olds
    a. Infant age at the time of MDA (1-5 months vs 6-11 months)
    b. Infant weight-for-age, length-for-age, weight-for-length at the time of MDA
    c. Infant sex
    d. Season of MDA dosing (malaria versus non malaria season) and SMC implementation in village (SMC given versus SMC not given)
    e. Cluster level coverage of SMC
    f. Cluster level baseline mortality (established at first census)
    g. Cluster and individual level coverage and number of administered azithromycin MDA doses
    h. District of residence
    i. Distance from the nearest health facility
    j. Household asset or income index
    k. Household WASH index
    l. National outreach strategy category (standard/advanced)
    m. Hard-to-reach village status assessed by the field workers

Secondary efficacy outcomes:
1. Prevalence of diarrhea, ARI, malaria, or other illnesses in the 14-day period before MDA visits
2. Mean weight-for age, length for age, weight-for-length, and mid upper arm circumference, and prevalence of moderate to severe stunting (LAZ < -2 / LAZ < -3) and moderate to severe wasting (WLZ < -2 / WLZ < -3) at 6-8 and 12-14 months of age.
3. Mortality (deaths / 1000 years at risk) among children who were 12–59-month-old when the latest azithromycin MDA took place in their village of residence.
    a. This will be analyzed since azithromycin MDA could elicit herd protection that could theoretically extend beyond the actual recipient population of the treatment.

Outcomes related to antimicrobial resistance:
1. Phenotypic macrolide resistance among *E. coli* strains isolated from stool samples or *S. pneumoniae* strains isolated from nasopharyngeal swabs among 4–14-month-old children, at one year after the MDA intervention has stopped (36 months after baseline).
    a. As descriptive information, we will provide the same information from a smaller group of children at baseline and when the intervention has continued for 12 and 24 months (i.e., after 4 or 8 rounds of MDA).
    b. For all isolates of azithromycin resistant E. coli or S. pneumoniae, we will also determine of phenotypic AMR against other antibiotics categorized by the World Health Organization (WHO) into "Access" group and/or that may have overlapping mechanisms: meropenem, ceftriaxone, ciprofloxacin, ampicillin, co-trimoxazole, and gentamicin for E.Coli, and oxacillin, erythromycin, vancomycin, ciprofloxacin, ampicillin, co-trimoxazole for S. pneumoniae.

2. Phenotypic macrolide resistance among *E. coli* strains isolated from stool samples or *S. pneumoniae* strains isolated from nasopharyngeal swabs among 49–59-month-old children, at 24 months after cluster enrollment to the trial, i.e. when the intervention has continued for 24 months (after 8 rounds of MDA).
    a. As descriptive information, we will provide the same information from a smaller group of children at baseline, at 12 months after enrolment (4 rounds of MDA), and at one year after the MDA intervention has stopped
    b. In here, we are primarily interested in the older age-group, since earlier research suggests that AMR prevalence will increase among the treated individuals, but we expect AMR not to spread to non-treated individuals.
    c. In a two-year MDA and follow-up period, 49–59-month-old children will never have received any azithromycin as part of the trial.
    d. For all isolates of azithromycin resistant E. coli or S. pneumoniae, we will also determine of phenotypic AMR against other antibiotics categorized by the World Health Organization (WHO) into "Access" group and/or that may have overlapping mechanisms: meropenem, ceftriaxone, ciprofloxacin, ampicillin, co-trimoxazole, and gentamicin for E.Coli, and oxacillin, erythromycin, vancomycin, ciprofloxacin, ampicillin, co-trimoxazole for S. pneumoniae.
3. Genetic markers of azithromycin and other antibiotic resistance (resistome) of intestinal and nasopharyngeal microbiota among 4–14-month-old children, at one year after the MDA intervention has stopped.
    a. As descriptive information, we will provide the same information from a smaller group of children at baseline and when the intervention has continued for 12 and 24 months.
    b. We are primarily interested in the post-intervention situation, since earlier research suggests that AMR prevalence will increase among the treated individuals soon after azithromycin treatment, but we expect the prevalence to go down after the intervention.
4. Carriage of bacteria with azithromycin resistance in children who received 1-4 rounds of MDA and who live in villages that have stopped MDA intervention 1 year prior to sampling (children who are 15-26 month-old at visit 11)
    a. As descriptive information, we will provide the same information from a smaller group of children one year after the MDA intervention has stopped.
    b. Earlier research has shown that AMR prevalence will increase among treated individuals. How and if this prevalence persists in these treated children 1 year after their treatment has finished and the MDA intervention ended in the villages they live is currently not known. We will assess phenotypic macrolide resistance and resistance against other antibiotics categorized by the World Health Organization (WHO) into "Access" group and/or that may have overlapping mechanisms: meropenem, ceftriaxone, ciprofloxacin, ampicillin, co-trimoxazole, and gentamicin for *E.coli*, and oxacillin, erythromycin, vancomycin, ciprofloxacin, ampicillin, co-trimoxazole for *S. pneumoniae*.
    c. We will assess genotypic markers of resistance and changes in the intestinal microbiome in children previously treated with azithromycin and we expect that the microbiome in these children has recovered 1 year post

treatment and is similar to the microbiome of children in the placebo group.

5. Carriage of bacteria with azithromycin resistance in children that received 1-4 rounds of MDA and who live in villages (tertiary villages in Kati region) that are still on MDA intervention 1 year prior to sampling (children that are 24-35 month-old at visit 9)
    a. As descriptive information, we will provide the same information from a smaller group of children 1 year after their individual treatment ended but the villages they live in are still on MDA intervention.
    b. Earlier research has shown that AMR prevalence will increase among treated individuals. How and if this prevalence persists in these treated children 1 year after their treatment has finished but the villages they live in are still on MDA intervention is currently not known. We will assess phenotypic macrolide resistance and resistance against other antibiotics categorized by the World Health Organization (WHO) into "Access" group and/or that may have overlapping mechanisms: meropenem, ceftriaxone, ciprofloxacin, ampicillin, co-trimoxazole, and gentamicin for *E.coli*, and oxacillin, erythromycin, vancomycin, ciprofloxacin, ampicillin, co-trimoxazole for *S. pneumoniae*.
    c. We will assess genotypic markers of resistance and changes in the intestinal microbiome in children previously treated with azithromycin and we expect that the microbiome in these children has recovered 1 year post treatment and is similar to the microbiome of children in the placebo group.

Outcomes related to the mechanism of azithromycin activity
1. Blood C-reactive protein concentration
2. Blood malaria parasitemia, and hemoglobin concentration
3. Fecal neopterin, myeloperoxidase, and alpha-1-antitrypsin concentrations

Safety outcomes:
1. Incidence of serious adverse events (SAE) within 14 days of study drug administration.
2. Incidence of adverse events (AE) within 14 days of study drug administration.

4.08. Data collection sites for various outcomes

Mortality and serious adverse event (SAE) data will be collected, and mortality-related questions answered using data from all the included 1150 villages (clusters) .

Addressing the other study questions will require more study personnel and additional equipment and infrastructure but a smaller sample size. Hence, we will answer them in a sub-sample of 60 villages, housing approximately 3,000 infants and located around four selected health centers close to the city of Kita (secondary outcome sample). Working in and from there health centers, there will be special teams of research personnel, that will collect information on the following outcome variables:
1. Mortality, as in all the other villages (see chapters 4.12 and 4.13 for details)
2. Morbidity for acute infections (see chapter 4.14 for details)

3. Growth and nutritional status (chapter 4.15)
4. Antimicrobial resistance (chapter 4.16)
5. Mechanism of azithromycin action (chapter 4.17)
6. Feasibility, acceptability and equity of intervention (chapter 4.18)
7. SAE and adverse events (AE) (chapter 4.19)

Whilst these 60 are in a more confined area than those constituting the primary outcome sample, they represent a similar, mostly rural population. Hence, there is no *a priori* reason to expect non-representativeness of the secondary outcome sample. To assess the representativeness, we will, however, compare selected baseline characteristics (such as child age, sex, weight-for-age, and household asset index) in the primary and secondary outcome sample before the statistical analysis. A dataset that excludes data from the secondary outcome sample will be used for a sensitivity analysis on the interventions effect on mortality.

Figure 2 shows a schematic illustration of the sample structure.

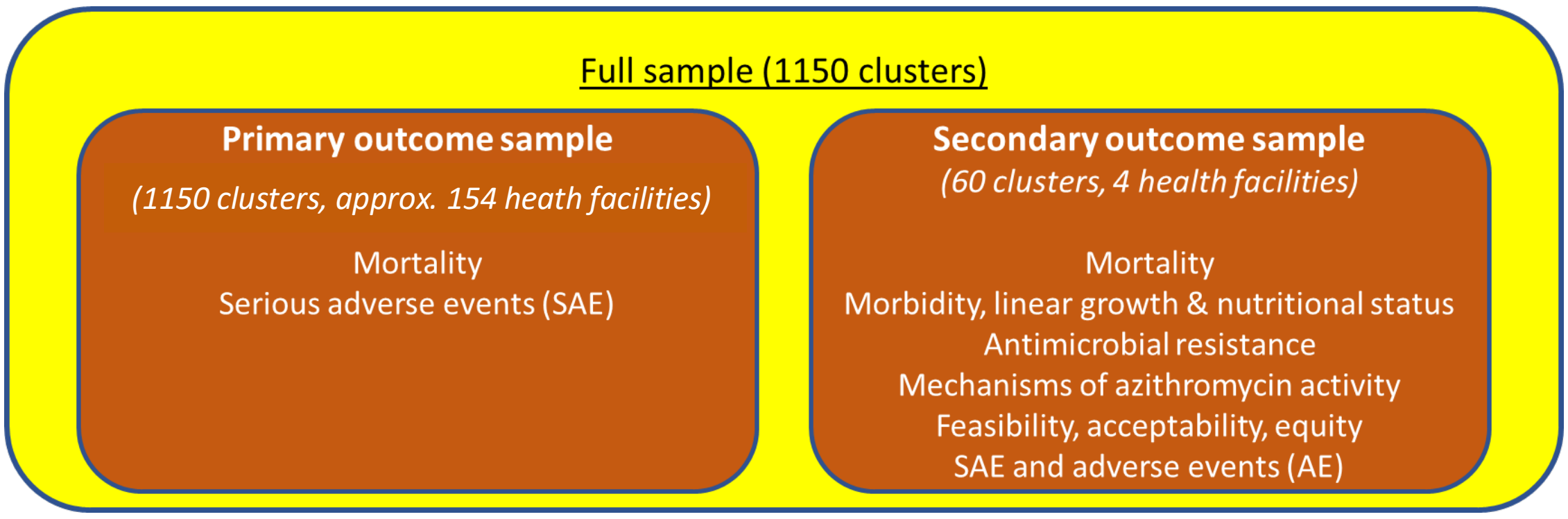

In addition to the above mentioned 60 villages in Kita, we intend to collect AMR samples and mechanistic study samples from infants and children living in a similar number of villages closer to Bamako, i.e., Koulikoro or Kati (tertiary AMR sample). For the mechanistic study in the tertiary sample, we will collect the biological samples during the rainy season (peak malaria transmission season). In addition to the AMR and mechanistic study samples, we will consider growth outcomes measurements (anthropometry) in the tertiary sample if the number of those measurements proved smaller than expected in the secondary sample villages.

4.09. Schedule and procedures for participant enrollment and follow-up

During a preparation phase, all villages / clusters in the included health districts of the three study regions will be listed and mapped. Scientists in the study team, working with the local health professionals, will review the map and exclude areas and villages that are considered urban, unsafe, inaccessible or unstable in population (in gold-mining areas, for example), to come up with a final list of invited clusters.

Senior-level study team supervisors, accompanied by local community volunteers and

local health service representatives, will visit leadership within each village / cluster, brief them about the trial (Appendix 1a) and invite them to participate. Villages that allow LAKANA activities to take place will be randomly allocated (in a ratio of 3:2:4) to three different intervention schemes: control, azi-quarterly, and azi-biannual . Village level permission will be given verbally and documented in writing in the project notebooks (Appendix 1b).

The included villages will be visited at quarterly intervals by GCP-trained study staff, accompanied by local community volunteers. At each round, there will be a two-week period, during which all households in the village will be enlisted, located, and visited if possible. At the first visit to each household, household representatives are briefed about the study (Appendix 2a) and invited to participate in the trial, including eight MDA treatments and a two-year follow-up. Those agreeing will be revisited eight more times at quarterly intervals. Households participating in an AMR sub-study (see chapter 4.15) will be visited one more time 12 months later.

At the enrollment visit, there will be a census of all household members and an interview with a household representative about some baseline socio-economic and other variables (including health facility that they normally use) as well as births and child deaths in the preceding year (to establish neonatal, infant, and under-five mortality rates at baseline). Children between 1 and 11 months of age will be assessed for MDA eligibility, weighed, and provided with study drug if eligible. At the subsequent seven visits, there will be an interview about recent births, child deaths, and health facility visits and again a study drug MDA to 1-11-month (29-364-day) old infants. At the 9th visit, there will be just the interview, but no MDA. At each of the visits 1-9, we will also ask about MDA treated infants' exposure to SMC (as a potential effect modifier) and document their EPI vaccination dates (from their vaccination card, as a marker of health service utilization).

There will be no upper or lower limit in the number of participants / study village, i.e. all households and infants who can be enrolled or treated with study drugs during any MDA round will be included in the study.

Infant age will be calculated with a computer, based on dates of visit and child's date of birth. Date of birth will be obtained primarily from a child health card, secondarily from caregivers' information (if exact date is reportedly known) and tertiarily estimated with the use of a time-bound event calendar.

Participating districts and villages will be enrolled into the study in a rolling manner over a period of 12 months and visited at regular 3-month intervals thereafter for 24 months. This will make logistics easier and reduce the risk of obtaining a biased estimate of azithromycin effect, in case this effect was modified by season and also by the order of MDA round. To shorten the trial period by six months, the last enrolled villages will be followed up only for 7 MDA rounds (30% of villages) or 6 MDA rounds (20% of villages).

As explained in general in chapter 4.08 and in detail in chapters 4.13 – 4.18, there will be additional data collection on several secondary outcomes in selected clusters and health centers. These data collections are timed in a way that maximises the possibility of observing a treatment effect and spreads the activities relatively evenly over the entire

trial period, thus maximising also staff efficiency.

Figure 3 shows an outline of the follow-up of individual LAKANA trial participants.

Outcome	Sample	District	Age group	Number of children	Year 1											
					1	2	3	4	5	6	7	8	9	#	#	#
Mortality	Full sample	A - F	01-11 mo	32,000	1			2			3			4		
Morbidity	4 CSCOMs	A	01-11 mo	1,350												
Antimicrobial resistance Participants & contacts	4 + 2 CSCOMs	A	04-14 mo 49-59 mo	1350+1350	1											
Mechanism of action	4 CSCOMs	A	06-08 mo 12-14 mo	1350+1350								3			4	
Growth	4 CSCOMs	A	06-08 mo 12-14 mo	1350+1350								3			4	
Feasibility	4 CSCOMs	A	01-11 mo	1,350				2								
Safety and adverse event	4 CSCOMs	A	01-11 mo	1,350	1							3			4	

Outcome	Year 2											
	1	2	3	4	5	6	7	8	9	#	#	#
Mortality	5			6			7			8		
Morbidity												
Antimicrobial resistance Participants & contacts	5											
Mechanism of action	5			6								
Growth	5			6								
Feasibility												
Safety and adverse event	5			6								

Outcome	Year 3											
	1	2	3	4	5	6	7	8	9	#	#	#
Mortality	9											
Morbidity												
Antimicrobial resistance Participants & contacts	9											
Mechanism of action												
Growth												
Feasibility				10								
Safety and adverse event												

Outcome	Year 4											
	1	2	3	4	5	6	7	8	9	#	#	#
Mortality												
Morbidity												
Antimicrobial resistance Participants & contacts	11											
Mechanism of action												
Growth												
Feasibility												
Safety and adverse event	11											

Additionally, we will collect samples at visit 11 from 15-26-months old children and at visit 9 in the Koulikoro/ Kati (tertiary AMR sample) region from 24-35-months old children.

4.10. Expected recruitment and loss-to-follow-up rates

The planned number of clusters (villages) to be included in the trial is approximately 1150, situated around approximately 154 health facilities. These villages are planned to be enrolled in the trial over a period of 18 months, i.e., 78 weeks. Hence, every week there will be on average 15 new clusters enrolled to the trial, situated around 1-3 new health facilities. The final number of clusters will depend on the cluster size; the village enrolment will be stopped before 1150 villages if 35,650 infants have been treated on MDA1 prior to that.

In each cluster, there are an estimated average of 35 infants of 1-11 months (29-364 days) of age. We assume to be able to obtain consent and provide study drugs to 31 (>85%) of them at each MDA round. Hence during the enrollment phase, we expect to provide MDA to 800 infants per week in the first three months, then 1600, 2400, and 3200 infants per week for three subsequent 3-month periods. We will then continue to treat approximately 32 00 infants / week for the next 12 months and then gradually stop treatments in the subsequent 9 months.

At each of the eight quarterly rounds of MDA, we thus expect to provide MDA to approximately 41,500 infants. At the first visit, all the treated infants will be new to the trial, on the subsequent visits, approximately 25% will be new infants, others will have received the study drugs already earlier. The maximum number of study drug treatments to any single individual will be four.

Since each time point will be assessed cross-sectionally, it will not be possible to calculate a traditional loss-to-follow-up rate. At each time, we expect to be able to get vital status information from approximately 80%, who were given study drug MDA at the previous home-visit. This proportion is based on prior experience from the Mordor trial, in which there was a 13% loss to follow-up at biannual home-visits.

4.11. Purchase, formulation, storage, and distribution of study drugs

The study drugs (azithromycin and placebo) will be donated by Pfizer Inc (New York City, NY, USA), that will manufacture and package them under good manufacturing practice (GMP) conditions and ship them to Mali.

The drugs will be packed in non-opaque, plastic bottles. Each bottle will contain the same amount of dry powder, either including 1.2 g of azithromycin or respective amount of the base powder.

A data manager at RTI International, an external partner for data support services, will randomly allocate 8 letter codes to azithromycin and 10 other letter coders to placebo and transmit the trial code to a pharmacist at Pfizer. Study drugs will be labelled with these letter codes. For each cluster, we will use bottles with two letter codes, one for visits between January and June and the other one for visits between July and December. Thus, there will be a total of 9 two-letter study drug regimens, of which 3 will be allocated to control (both letter codes for placebo), 2 will be for azi-quarterly (both letter codes for azithromycin) and 4 will be for azi-biannual (one letter code for placebo, the other one for azithromycin).

During shipment and storage at the study site, the study drug bottles will be stored in locked cabinets, in a dry place with a temperature between 15°C and 30°C.

For consumption, a drug bottle will be reconstituted with 15 ml of clean (commercial bottled) water to make 30 ml of study drug suspension. The reconstituted mixture will thus contain either 40 mg / ml azithromycin (active drug) or no azithromycin (placebo).

The dose given to eligible children will be 20 mg / kg, i.e. 0.5 ml / kg, rounded up to the nearest 0.2 ml. A data collector will measure and give the dose to study participants with a single-use sterile syringe, under direct observation. There will not be any planned observation period of the infant immediately after the study drug administration, but the caregivers are advised to contact the study team if anything unusual happens. If a study team member becomes aware that an infant has vomited soon after the study drug ingestion (within approximately 15 minutes), they will give a new, similar-size dose to the infant in question.

At any village (cluster), all infants will receive study drug of the same letter code. Hence, a bottle can be reconstituted and used for several participants on the same day. An expected mean dose will be 3 ml, i.e. one bottle may be enough for up to 10 participants. For each MDA round, we would thus need an average of 2,500 bottles of placebo and 2,000 bottles of azithromycin. Given the 8 MDA rounds and an assumption of 80% wastage in storage (with several letter codes for each product and a geographically a wide operation), we estimate to need 100,000 bottles of placebo and 80,000 bottles of azithromycin for the whole trial.

At the end of each day, the remaining reconstituted study drugs will be brought back to the study office. At the study office, the left-overs will be discarded, according to national instructions.

4.12. Data collection on infant and child mortality

At the enrollment visit, a data collector hired by the the study team will interview a household head to collect information on household composition. S/he will record the houshold members in a password-protected and encrypted electronic trial database, to which s/he can refer to and which s/he will update during subsequent visits. The database will include the following information on each member of the household: Sex, name, date of birth, age, date of start of follow-up (enrollment of household or in-migration), date of end of follow-up (death, withdrawal, or out-migration)

At each of the subsequent visits, the data collector will check the vital status (alive, dead, out-migrated, or unkown) of the previously registered household members and record any new births and in-migrations. If a child death - is noticed, there will be a special set of questions on the date and cause of death. Cause of death will be classified into trauma, acute illness of less than two weeks duration, prolonged illness of more than two weeks of duration, or other, i.e. no extensive verbal autopsies or other cause of death ascertainment will be done. Valid vital status information can be obtained from a parent or other caretaker of the child who lives in the same household; if such a person is not available, the information will be considered missing.

Mortality will be expressed as deaths / 1000 years at risk. Each 3-month interval will contribute to mortality calculation. Children will be included in the analyses if they were present in the household at the beginning of the interval and their vital status (dead or alive) was known and provided to the study team at the subsequent visit, by an eligible adult (see above for eligibility criteria). Time in follow-up will be the number of days between the beginning of the interval and death, outmigration, or end of interval, whichever came first. All mortality data i.e. from both the primary outcome sample and the secondary outcome sample will be used for the primary mortality outcome analysis.

Mortality rates will be calculated separately for 29-364 days old infants (the primary target population for the intervention) and for 12.00-59.99 month old children (who have not themselves received the MDA intervention). The indicated figures refer to the subject’s age at the beginning of the interval.

4.13. Adaptive design: possible changes after an interim analysis

An interim analysis will be conducted when approximately 60% of the planned 3-month time-intervals have been completed. The analysis results will be seen and reviewed only by the trial data safety and monitoring board (DSMB) that will advise the study team on trial continuation, according to its charter. Based on its adaptive design, the trial conduct will be altered in case the interim analysis gives either of the following results:

1. There is evidence of a mortality benefit in both of the azithromycin groups as compared to the placebo group but no statistically significant difference between the two azithromycin groups.

   a. In this scenario, the team will drop the placebo arm and re-randomize the previous placebo-clusters into either of the two azithromycin groups, at 1:1 allocation ratio. All trial participants will be informed about the change and a new consent will be requested for participation in the revised trial.
   b. Before implementation, the participant information leaflet and an amended consent form will be submitted to the appropriate ethics committees and institutional review boards (IRB) for review and approval.
   c. Subject to endorsement by the trial data safety and monitoring board (DSMB) before the onset of data collection, the team proposes to consider a mortality difference in an interim analysis statistically significant if a 2-sided p-value is smaller than 0.001 (Peto et al., 1976).
   d. After the results from the azi-quarterly and azi-biannual groups comparison are available, the team will offer to work with the Malian Ministry of Health on implementation of a public health MDA intervention in Kayes, Kita, and Koulikoro and possibly Bougouni and Segou regions of Mali, choosing the MDA regimen (quarterly or biannual) based on the trial data.

2. There is evidence of a mortality benefit in one or both of the azithromycin groups and also a statistically significant difference between the two azithromycin groups.
   a. In this scenario, the provision of the study MDA will be stopped, and the team will offer to work with the Malian Ministry of Health on implementation of azithromycin MDA in Kayes, Kita, and Koulikoro and possibly Bougouni and Segou regions of Mali, choosing the MDA regimen (quarterly or biannual) based on the trial data.

A separate trial (SANTE) in Sikasso Region is evaluating the benefits of azithromycin provided to pregnant women and to infants at the 6-week EPI visit. The combined results of the SANTE trial and the newly proposed trial will provide important evidence for decision-making around the optimal strategy for delivering azithromycin for child survival in the whole of Mali.

The adaptive design is illustrated in Figure 4 below.

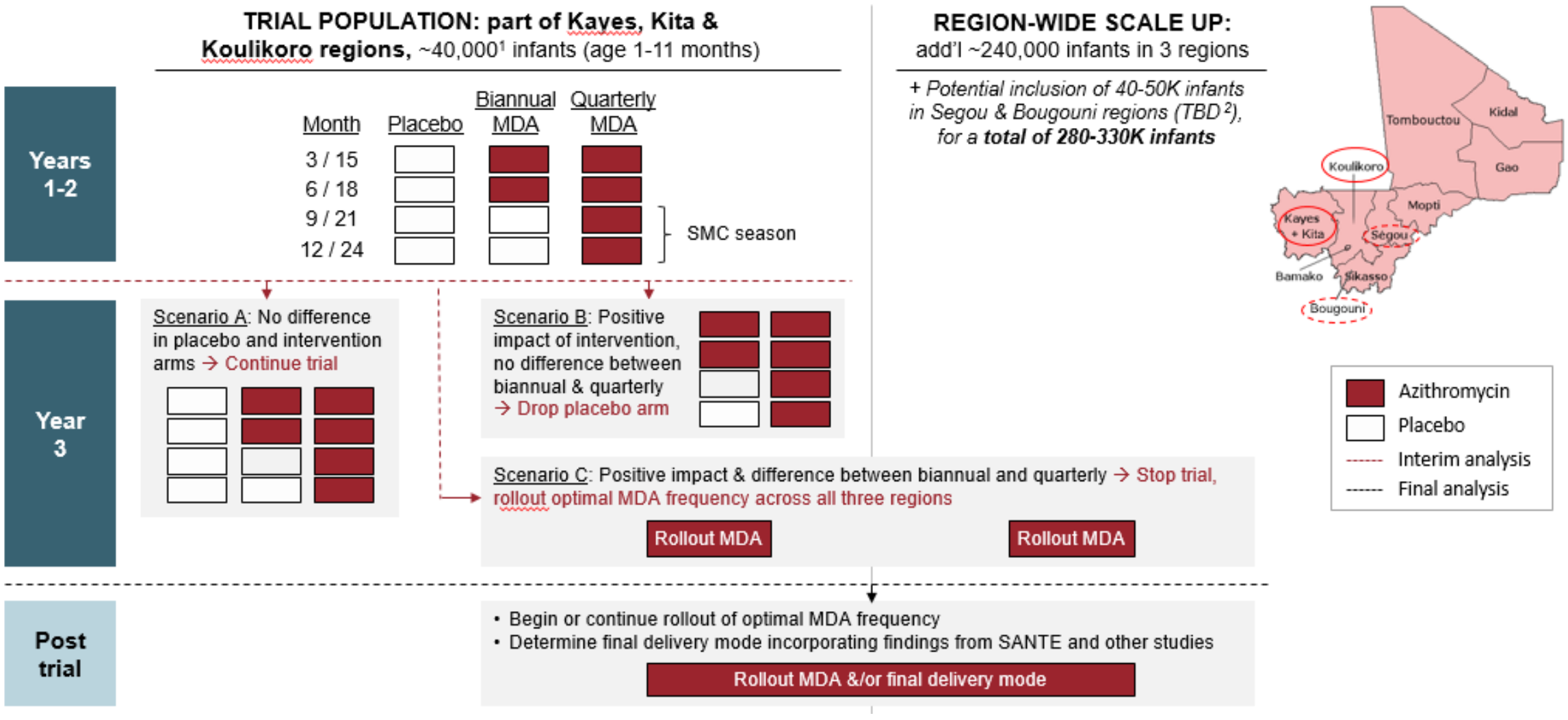

4.14. Data collection on infant morbidity

Earlier trials and other studies have suggested that azithromycin MDA might also have an impact on child morbidity. Whilst it would be difficult to assess morbidity reliably on an individual level in all LAKANA participants, we intend to collect morbidity related information among infants who live in the 60 villages selected for additional outcome assessment (secondary outcome sample). These villages are served by four health centers, where we will establish a LAKANA office and post research staff for the additional data collection.

During the normal MDA home visits in these selected villages, the LAKANA team will ask if a child has been ill in the preceding 14 days, with fever with respiratory symptoms (acute respiratory infection, ARI), fever without respiratory symptoms (proxy for malaria), diarrhea, or any other symtoms. These questions will be asked about each 4-14 month old infants and children, i.e. those who were eligible to receive the study drug intervention at an earlier MDA round.

From the collected data the team will calculate incidence of various types of illness episodes in the three study arms. The team will then calculate relative risks for the illnesses in azi-quarterly and azi-biannual groups, as compared to the control group.

4.15. Data collection on infant growth and nutritional status

A relatively recent meta-analysis suggested that antibiotic treatment of children in low-income countries might improve their linear growth (Gough et al., 2014). The review did not include enough studies on infants, so the question of antibiotic effect on infant growth is still open. It is, however, possible, that azithromycin might also promote growth in this age group (in which length and weight gain is faster than among older children), and hence it justifiable to study growth as a secondary outcome in a mortality trial.

The sub-sample will be formed from children, who reside in the 60 villages around the four selected health centers (secondary outcome sample). In these villages, data collectors will take anthropometric measurements from 6-8 and 12-14 month old children on study visits 6, 7, 8, and 9, i.e. at 15, 18, 21, and 24 months after the enrollment of the village into the trial. At these visits, the 6-8 month-old and 12-14 month old children will have received two and four doses of study drugs, respectively, unless they migrated into the village later, were absent, unlocated or declined study drug administration. Thus this sampling frame ensures maximal exposure to the study drug at size assessment.

At each time point, we expect to measure a mean of 7.5 children / village, i.e. the total sample size will be 1800 children, i.e. approximately 600 per group. This sample size will provide the study with 80% power to detect difference of approximately 0.2 SD units in anthropometric indices, which is a typical achievement in nutritional supplementation trials (Dewey and Adu-Afarwuah, 2008).

Measuments taken from the sub-sample include length, weight, and mid upper arm circumference (MUAC). Length will be assessed using a commercial length board (ShorrBoard®, WEIGH AND MEASURE, LLC, Olney, Md, USA) and recorded to the nearest 1 mm. Weight will be assessed using an electronic infant weighing scale (SECA 354) with reading increments of 10g. MUAC will be measured with non-stretchable plastic insertion tapes and the results will be recorded to the nearest 1 mm. All measurement will be done in triplicate.

The data collectors measuring anthropometrics will be trained and standardized prior to beginning of data collection, modified from the procedures used in the WHO 2006 growth standard study (WHO, 2006). Standardization sessions will be repeated every 6 mo during data collection. We will calculate weight-for-age (WAZ), length-for-age (LAZ), weight-for-length (WLZ), and arm circumference-for-age z-scores using the WHO 2006 Child Growth Standards. We will also calculate the percentage of children who are moderately or severely stunted (LAZ < -2 / -3), or moderately or severely wasted (WLZ< -2 / -3). Those with wasting will be referred for nationally recommended diagnostics and treatment.

4.16. Data collection on antimicrobial resistance

One of the major concerns in the use of azithromycin MDA is the possibility of increasing AMR, resulting in the loss of mortality benefits, and possibly even increasing health problems. As explained in chapter 3.03, macrolide resistance has earlier been observed in the treated individuals soon after azithromycin MDA, but there is relatively little data on longer follow-up or spread of AMR to unexposed populations. In LAKANA, we will therefore monitor prevalence of azithromycin resistance before, during and after the intervention and both among children receiving and those not receiving MDA with the study drugs.

To allow comparability to national and international AMR statistics, we will primarily study AMR with a traditional phenotypic culture method, isolating individual colonies of selected indicator bacteria (*S. pneumoniae* and *E. coli*) from children and assessing

bacterial growth on a Petri-dish in the presence or absence of azithromycin. To maximise comparison of these surveillance cultures with antimicrobial resistance results from other azithromycin MDA trials (such as the AVENIR trial in Niger), details of sample collection and processing methods will be harmonised between the different studies.

Studying individual colonies of selected indicator bacteria is a widely used approach for AMR assessment, but it gives no information on possible antibiotic effects on other colonies of the same species or any of the other bacterial species (some of which may be clinically much more important than the indicator bacteria). We will therefore also employ genetic methods to study microbiota composition and the presence of molecular markers of AMR in host bacteria. As sample collection for the AMR component will take three years and molecular methods are rapidly developing, we will store samples for these genetic (and possible other) analyses until the completion of data collection and choose the exact molecular methods only at that time point. Similar to the indicator bacteria analyses, these sample collection and processing for these studies will be harmonised with the AVENIR trial in Niger.

In the first phase, we will thus address the following questions:

1. What is the impact of biannual or quarterly azithromycin MDA to 1-11 month (29-364 days) old infants on the prevalence of phenotypic azithromycin resistance among *S. pneumoniae* or *E. coli* strains isolated from 4–14-month-old children, who have received 1-4 rounds of azithromycin MDA?
2. What is the impact of biannual or quarterly azithromycin MDA to 1-11 month (29-364 days) old infants on the prevalence of phenotypic azithromycin resistance among *S. pneumoniae* or *E. coli* strains isolated from 49–59-month-old children, who live in the same Malian communities but have not received azithromycin MDA?
3. What is the impact of biannual or quarterly azithromycin MDA to 1-11 month (29-364 days) old infants on the prevalence of phenotypic azithromycin resistance among *S. pneumoniae* or *E. coli* strains isolated from 15-26-month-old children, who have received 1-4 rounds of azithromycin MDA and live in a village that stopped azithromycin MDA 1 year before?
4. What is the impact of biannual or quarterly azithromycin MDA to 1-11 month (29-364 days) old infants on the prevalence of phenotypic azithromycin resistance among *S. pneumoniae* or *E. coli* strains isolated from 24-35-month-old children, who have received 1-4 rounds of azithromycin MDA 1 year before and who live in a village that continued azithromycin MDA?

To ascertain if clinical disease is being influenced by azithromycin MDA, we will also study AMR in bacteria isolated from stools with children presenting at any of the four selected health facilities because of dysentery (bloody stools) or blood, urine, or cerebrospinal fluid of 1–11-month-old infants or older children hospitalized in the nearby Kita District Hospital because of a severe, septic illness. For this purpose, we will establish a basic microbiology laboratory in this hospital and support its operations during the LAKANA trial. We have chosen Kita for this purpose, because it is the largest hospital in the Kita region and because we will establish our LAKANA biospecimen processing and storage laboratory in this hospital. Kita is also relatively near Bamako, which facilitates support and oversight by the CVD-Mali laboratories and the LAKANA research team.

In the main CVD-Mali laboratory in Bamako (level 3) or Kita laboratory (level 2), the study team will perform microbiological analyses that can be fed back to clinicians to support clinical management of infections. The clinical physicians can then target their treatment based on the microbiological findings on bacterial type and basic bacterial characterization, such as Gram stain. In addition to this simple analytical laboratory, the team may establish a more basic field laboratory (level 1) in a health facility in Koulikoro. At this facility, the study team can process, and store collected AMR samples and perform initial bacterial growth analysis using an automated suspension cultivation method. All AMR analyses will be performed in the main CVD-Mali laboratory in Bamako (level 3). In additional bacterial culture, the team will also perform resistome analysis and other molecular AMR testing from the isolated bacterial colonies, with methods that are harmonized with those of the AVENIR trial in Niger.

Our assumption is that among the treated children, AMR prevalence will increase during the intervention and return to pre-study levels after the intervention has stopped. Among the older children, who live in the same treatment communities but who have not themselves received any MDA, we expect no change in AMR prevalence. The assumed AMR prevalence over time in the two samples is shown in Figure 5 below.

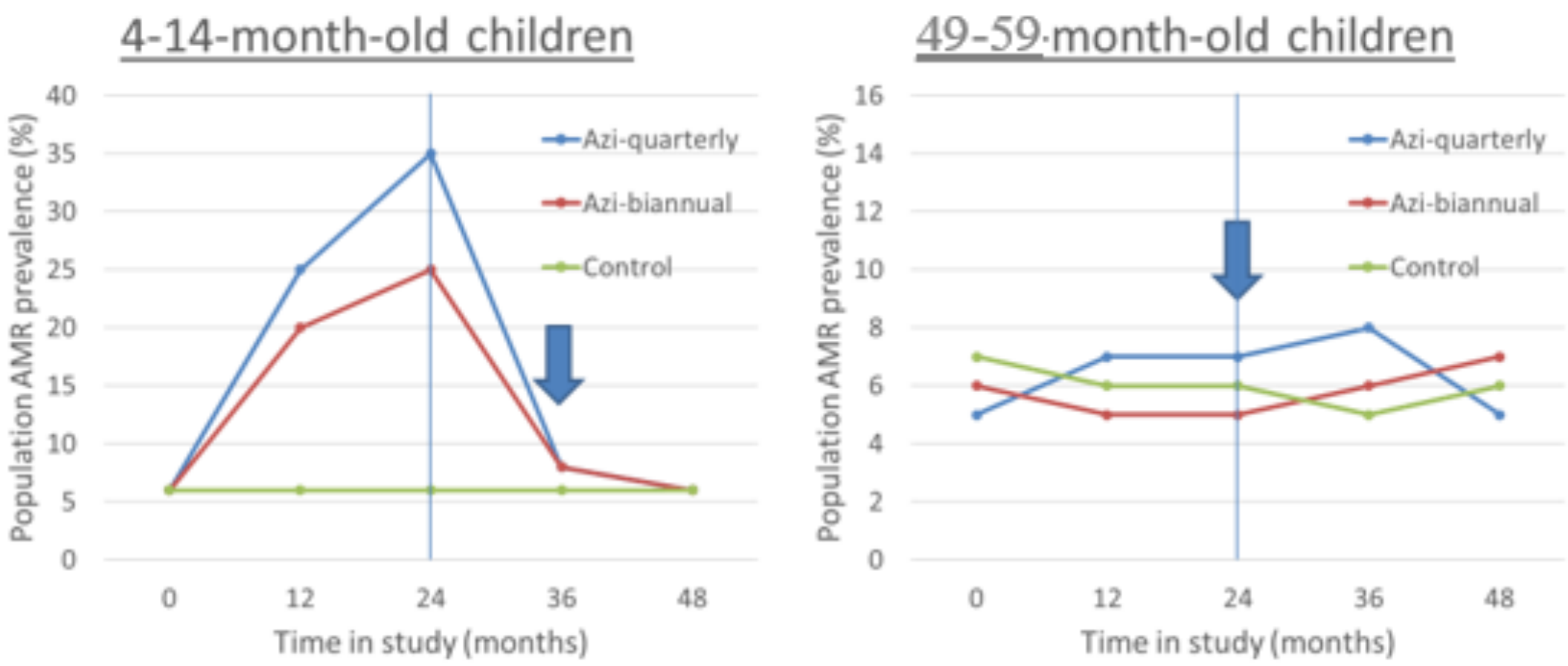

The hypothesis to be tested from the 4–14-month-old children is that azithromycin does not cause a sustained increase in AMR, i.e., that AMR prevalence will not be higher in the azithromycin clusters than the control clusters at one year after the intervention. For the older children (who received no MDA), the respective hypothesis is that AMR prevalence will not be higher in the azithromycin clusters than the control clusters at any point, not even at the peak of the intervention – i.e at 12-24 months from the study start. Thus, for AMR, we have a non-inferiority hypothesis – i.e., that azithromycin MDA can be used to reduce infant mortality without a clinically significant increase in AMR prevalence. In an exploratory analysis, we will also compare AMR prevalence in the two azithromycin groups, to investigate if quarterly MDA selects for more AMR that the biannual MDA.

The suggested set-up will allow us to describe concomitant time trends in the prevalence of AMR in indicator bacteria isolated from healthy children, the incidence of septic

diseases, and the prevalence of AMR in bacteria that are causing severe disease. This will facilitate understanding about the clinical significance of AMR, possibly caused by the MDA intervention.

For hypothesis testing on the prevalence of AMR in indicator bacteria, we will need a total of 1,350 children. These children will be identified in the 60 villages around the four health centers selected for secondary outcome data collection in Kita. All 4–14-month-old and 49-59 month old children in this sub-sample will be invited to provide biological samples for AMR analyses at enrollment (visit 1) and at 12 months (visit 5), 24 months (visit 9) and 36 months (visit 11) after the inclusion of the village into the study.

To facilitate the AMR sample collection before study drug administration, the team making home visits in this sub-sample of clusters will include a study nurse. Before any MDA, this nurse will collect one nasal and three rectal swabs from the study infants, in a tent set up in the village that is being visited.

To assess whether AMR carriage persists on an individual level we will sample children at the final time point (visit 11) who have received 1-4 MDA doses and who live in a village that stopped MDA intervention 1 year earlier (children at 15-26 month-old). Additionally, we will sample children (24-35 months old) at visit 9 (Koulikoro/Kati region) who have received 1-4 MDA doses 1 year earlier, but live in a village that continued MDA intervention.

After collection, the nasopharyngeal swabs will be placed in STGG media (skim milk, tryptone, glucose, and glycerin), one rectal swab will be placed in Cary-Blair media, the second rectal swab will be placed into will be placed in DESS media (DMSO/EDTA/saturated sodium chloride) and the third rectal swab will be frozen without preservatives in a cryovial. The third rectal swabs will be flash frozen in dry shippers containing liquid nitrogen that has been absorbed in the container linings. Other samples will be placed in a +4°C cooler box. All samples will be transported within 2-4 hours to a study laboratory in Kita or to the main laboratory in Bamako, where a laboratory technician will process them and store them at -80°C: From the Kita laboratory, samples will be periodically transported to the main CVD-Mali laboratory for processing.

Nasopharyngeal swabs will be primarily cultured on chocolate agar with an optochin disc, to identify pure colonies of *Streptococcus pneumoniae*. Rectal swabs will be primarily cultured on MacConkey plates, to identify pure clones of *Escherichia coli*. Isolated colonies will then undergo phenotypic AMR analysis with bacterial culture on Petri-dishes supplied with Azithromycin containing antibiotic strips (E-test).

In the first phase, we will determine azithromycin sensitivity from all the samples collected from 4–14-month-olds at 24 months and from 49-59 month olds at 36 months (n = 1,350 per time point and age group) and from 1/3 of the samples at the other time-point (for descriptive purposes, not for hypothesis testing). We will also determine azithromycin sensitivity from samples collected from 15-26-month-olds at 36 months (Kita region) and 24-35-month-olds at 24 months (tertiary AMR samples). We will then calculate AMR prevalence for in *S. pneumoniae* and *E. coli*, for each of the time points and each of the three trial arms separately. At each time point, we will calculate the

difference (95% confidence interval, CI) in AMR prevalence between the control and azi-biannual clusters on one hand and between the control and azi-quarterly clusters on the other. The pre-set non-inferiority margin is 10 %-points, i.e. if the entire 95%CI of the difference in less than 10%-points, the data will be considered supportive of the non-inferiority hypothesis.

The 10% non-inferiority margin has been obtained by a DELPHI method talking to many infectious disease specialists – and also used in the WHO coordinated ABCD trial. The margin is expressed as an absolute and not a relative difference in AMR prevalence, because absolute increase in AMR prevalence will indicate how many additional possible exposures with resistant bacteria the study area population may face. Thus, an increase from 1% to 11% or 50% to 60% will provide 10 new “risk” persons in a group of hundred even if one goes up in relative terms by 1000% and the other only 20%.

We will retain and freeze at -80°C the original swabs after plating as well as the identified bacterial colonies, to allow further analyses with the indicator bacteria or any other intestinal bacteria or other microbes with later evolving methods. Bacterial colonies will be stored at two points for later analyses: during primary culture when organisms other than the main targets are grown and from the antibiotic sensitivity plate in order to capture resistance diversity within the target organisms. The stored cultures can subsequently be analysed through standard culture or by molecular techniques.

Figure 6 summarises samples taken for AMR analyses and stored at -80°C. In total, there will be an estimated of 54,000 vials, without the so called “clinical isolates”, taken from ill children. In total, these stored samples thus will fill one -80°C freezer.

Duration of intervention in community	Child age	# of given MDA rounds / village	Biological specimen	Storage media	Estimated # of aliquots	Volume / aliquot	Storage temperature	# of children
0 months	4-14 mo & 49-59 mo	0	Nasopharyngeal swab	STGG media	2	100 ug	-80°	1350 + 1350
0 months	4-14 mo & 49-59 mo	0	Rectal swab	Cary-Blair media	1	100 ug	-80°	1350 + 1350
0 months	4-14 mo & 49-59 mo	0	Rectal swab	DESS Media	1	100 ug	-80°	1350 + 1350
0 months	4-14 mo & 49-59 mo	0	Rectal swab	None, stored dry	1	100 ug	-80°	1350 + 1350
12 months	4-14 mo & 49-59 mo	2	Nasopharyngeal swab	STGG media	2	100 ug	-80°	1350 + 1350
12 months	4-14 mo & 49-59 mo	2	Rectal swab	Cary-Blair media	1	100 ug	-80°	1350 + 1350
12 months	4-14 mo & 49-59 mo	2	Rectal swab	DESS Media	1	100 ug	-80°	1350 + 1350
12 months	4-14 mo & 49-59 mo	2	Rectal swab	None, stored dry	1	100 ug	-80°	1350 + 1350
24 months	4-14 mo & 49-59 mo	6	Nasopharyngeal swab	STGG media	2	100 ug	-80°	1350 + 1350
24 months	4-14 mo & 49-59 mo	6	Rectal swab	Cary-Blair media	1	100 ug	-80°	1350 + 1350
24 months	4-14 mo & 49-59 mo	6	Rectal swab	DESS Media	1	100 ug	-80°	1350 + 1350
24 months	4-14 mo & 49-59 mo	6	Rectal swab	None, stored dry	1	100 ug	-80°	1350 + 1350
12 months post-intervention	4-14 mo & 49-59 mo	0	Nasopharyngeal swab	STGG media	2	100 ug	-80°	1350 + 1350
12 months post-intervention	4-14 mo & 49-59 mo	0	Rectal swab	Cary-Blair media	1	100 ug	-80°	1350 + 1350
12 months post-intervention	4-14 mo & 49-59 mo	0	Rectal swab	DESS Media	1	100 ug	-80°	1350 + 1350
12 months post-intervention	4-14 mo & 49-59 mo	0	Rectal swab	None, stored dry	1	100 ug	-80°	1350 + 1350
12-month post intervention	15-26 mo	0	Nasopharyngeal swap	STGG media	2	100ug	-80°	1000
12-month post intervention	15-26 mo	0	Rectal swab	Cairy-Blair media	1	100ug	-80°	1000
12-month post intervention	15-26 mo	0	Rectal swab	DESS Media	1	100ug	-80°	1000
12-month post intervention	15-26 mo	0	Rectal swab	None, dry swab	1	100ug	-80°	1000
12-month post treatment (in villages with MDA)	24-35 mo	0	Nasopharyngeal swap	STGG media	2	100ug	-80°	600
12-month post treatment (in villages with MDA)	24-35 mo	0	Rectal swab	Cairy-Blair media	1	100ug	-80°	600
12-month post treatment (in villages with MDA)	24-35 mo	0	Rectal swab	DESS Media	1	100ug	-80°	600
12-month post treatment (in villages with MDA)	24-35 mo	0	Rectal swab	None, dry swab	1	100ug	-80°	600

To gain a standardized understanding of antibiotics utilized for common clinical syndromes in the study area, we will collect and analyse national and regional guidelines and protocols on antibiotic prescribing on an annual basis. We also aim to monitor the amount of common antibiotics sent out to health facilities from the six study districts. A

more detailed review of antibiotic prescription will be done in the four health facilities in which the AMR sample collection takes place.

4.17. Data collection on mechanisms of action for azithromycin

Azithromycin MDA has been associated with reduced child mortality, but its mechanism of action in this respect remains unclear. The drug is a broad-spectrum antibiotic with strong activity against many bacterial species and it also has anti-inflammatory properties (Kwiatkowska and Maślińska, 2012). A recent report from the MORDOR trial in Niger documented a lower population prevalence of malaria in communities where under-five-year-old children had received azithromycin than in control villages, but it is not known if this was caused by a direct antimalarial effect or through an indirect pathway (Arzika et al., 2019). Antibiotic treatment might also affect infants' immunity through multiple pathways, including alterations in enteric microbiota and pathogens, enteric and general inflammation, and immune development.

The current trial provides an excellent opportunity for a sub-study to test hypotheses about mechanisms of azithromycin activity against child mortality. In LAKANA, we will test three hypotheses that have been posed but not tested directly earlier, i.e. that azithromycin MDA eliminates malaria parasitaemia and reduces inflammation in the treated host. We will also store biological samples for later laboratory analyses on other hypotheses, to be more specifically determined once the mortality effect is known and some ongoing mechanistic studies elsewhere have been completed.

These are the three hypotheses that we will test in the first phase:

1. Biannual or quarterly azithromycin MDA to 1-11 month (29-364 days) old infants eliminates their asymptomatic malaria, reduces population prevalence of malaria parasitemia, and improves the infants' mean blood hemoglobin concentration.
    a. Malaria control may be a factor influencing mortality. Azithromycin effect on malaria can in the LAKANA trial be assessed both with direct parasitemia analysis among 4–11-month-old children before and 2 weeks after the fourth azithromycin or placebo treatment and by comparing prevalence of malaria parasitemia among 6–8-month-old and 12–14-month-old children who live in the study villages and who have been eligible to receive 2-4 doses of the study drug. As a marker of health consequences of malaria treatment, we can additionally study blood hemoglobin concentration in the same children.
    b. To assess community-level prevalence of malaria across communities at the end of the intervention, and a possible carry-over effect, we will also study prevalence of malaria among 1-59-month-old children. This survey will be conducted on visit 9, i.e., when the intervention has lasted for 2 years. The sample size for this survey will be 3000 children aged 1-59 months. The Probability-proportional-to-size (PPS) sampling method will allow that each household in the sample has the same probability of being selected. In each household selected, all 1-59-month-old children will be tested with a malaria rapid diagnostic test.

2. Biannual or quarterly azithromycin MDA to 1-11 month (29-364 days) old infants reduces their general inflammation.
    a. General inflammation may be an important driver of subsequent disease acquisition and activity. This may be driven by bacteria resident on mucosal surfaces or through the inflammatory modulating properties of azithromycin and can be reflected using simple measure of CRP in small volumes of plasma, in the same groups of children from whom we will do the malaria analyses.
    b. Larger plasma samples and peripheral blood mononuclear cells from 6–8-month-old and 12–14-month-old children who have been eligible to receive 2-4 doses of study drug will be used for more extensive inflammatory profiling as previously described in our studies on immune activation, inflammation and antibiotic impact in children with HIV (Fitzgerald et al., 2016; Prendergast et al., 2016; Bourke at al., 2019). We will focus on markers we have found to distinguish children with evidence of increased inflammation and immune activation which include, IL6, IL8, IL10, TNF, IL1-RA, soluble adhesion molecules, Serum Amyloid A, markers of disordered thrombogenesis, and markers of cell activation and proliferation (HLA-DR, CD38, Ki67) on naïve and memory CD4 and CD8 populations.

3. Biannual or quarterly azithromycin MDA to 1-11 month (29-364 days) old infants reduces their intestinal inflammation.
    a. In addition to any impact on general inflammation, intestinal inflammation and environmental enteric dysfunction (EED) could be an important driver of disease. This will be studied by measuring neopterin, myeloperoxidase, and alpha-1-antitrypsin (and possibly other biomarker) concentrations in stools collected from 4–11-month-old infants before and two weeks after the fourth MDA and among 6–8-month-old and 12–14-month-old children who live in the study villages and who have been eligible to receive 2-4 doses of the study drug.

After the trial, we propose to test the following hypotheses (and possibly others) if they are still valid then. For LAKANA, the budget includes only sample collection and storage for these analyses, funding for the actual analyses will be sought later.

4. Biannual or quarterly azithromycin MDA to 1-11 month (29-364 days) old infants leads to improved immunity and vaccine response among 6–14-month-old children.
    a. Vaccine efficacy is reduced in low-income settings, potentially due to heavy microbial load (Parker et al., 2018). In some settings, it has been possible to improve vaccine response by sanitation and hygiene interventions (Church et at, 2019) or antimicrobials given before vaccination (Uchiyama et al., 2014). This suggests vaccine response enhancement as one of potential mechanisms by which azithromycin could reduce infant mortality. As markers of vaccine response, we will in the LAKANA trial measure antibody titers to rotavirus, tetanus, and measles vaccines. Rotaviral and tetanus antibody concentrations will be analyzed

from plasma samples collected at 6-8 months of age and measles antibody concentration at 12-14 months of age.

   b. Azithromycin could also modulate cellular immunity, either directly or indirectly, as we have previously described for the antibiotic cotrimoxazole (Bourke et al., 2019). We and others have shown that the ratio of naïve-to-memory CD4+ and CD8+ T-cells was much lower in African children than in resource-rich settings. Our data shows that this is probably driven by infection driving T cell activation and memory cell expansion despite greater thymic output (Klein et al., manuscript in preparation). In LAKANA, we will determine if azithromycin modulates thymic output, the ratio of naïve to memory CD4 and CD8 sub-populations, their cytokine production and proliferation, and T cell receptor repertoires (TCR) (Gibbons et al., 2014; Sandgaard et al., 2014; Gkazi et al., 2018).

5. Biannual or quarterly azithromycin MDA to 1-11 month (29-364 days) old infants eliminates specific pathogenic microbes from their intestinal tract.
   a. Elimination of specific pathogenic microbes is one of the key hypotheses on how azithromycin might reduce child mortality. In LAKANA, this hypothesis can be tested using TaqMan cards of 40-50 potentially pathogenic intestinal microbes and stored stool samples from the trial participants. The analyses can be done from paired stool samples collected from 4–11-month-old infants before and two weeks after the fourth MDA (to track what happens with one MDA dose on an individual level) and from 6–8-month-old and 12–14-month-old children who live in the study villages and who have been eligible to receive 2-4 doses of the study drug (to see a cumulative effect of multiple doses).

6. Biannual or quarterly azithromycin MDA to 1-11 month (29-364 days) old infants modulates their critical bacterially driven metabolic pathways.
   a. Metabolic profiling in combination with fecal microbiome will be performed in stored plasma, urine and stool as previously described (Wijeyesekera et al., 2019). Fecal and urine metabolites will be determined by ^{1}H nuclear magnetic resonance (NMR) spectroscopy. The extent to which the metabolic signature obtained reflects the corresponding microbiota state will be possible combining microbiome data. Special attention will be given to the presence of short-chain fatty acids (acetate/propionate/butyrate/valerate and their isoforms) as well as studying bile-acid metabolism, which play a pivotal role in many aspects of microbial ecology, most importantly in maintaining community homeostasis; their potential contribution to ‘mono-domination’ and ‘stochastic dysbiosis’ following azithromycin administration.

7. Biannual or quarterly azithromycin MDA to 1-11 month (29-364 days) old infants does not cause a sustained reduction in the diversity and immune impact of their intestinal microbiota (measured at one year after the last provided MDA).
   a. The composition of the microbiota is known to be influenced by infections and antibiotic usage. Modelling these changes over time is typically challenging due to the large number of potential interactions between

species. However, by using diversity relative to baseline as a measure of displacement from an 'equilibrium state', our group has recently developed a simple analytical model of microbiota reconstitution after antibiotic treatment (Shaw et al., 2019). This model successfully shows that short courses of common antibiotics (including ciprofloxacin, clindamycin, minocycline, and amoxicillin) can have year-long effects in adults, causing transitions to alternative microbiota states (quantified using diversity relative to baseline). We will employ this modelling approach to quantify the *in vivo* impact of antibiotics on our patients. By fitting the model to different patient characteristics (*e.g.,* age, weight, MDA) we will also be able to compare model parameters and comment on the potentially different impacts of antibiotics between groups. Simultaneously quantifying microbiota stability and immunity over time, we will be able to study how the two interact and their relative contribution to clinical outcomes using multivariable time series modelling.

Data collection for these analyses will be done in the same sub-sample of 60 clusters as used for the AMR work and other secondary outcomes. From these clusters, we will invite three groups of children for the mechanistic studies:

1. 4–11-month-old infants living in communities that will receive their 4th MDA.
2. 12–14-month-old infants who live in communities that will receive their 6th, 7^{h}, 8th study MDA and at 9th study visit.
3. 6–8-month-old infants who live in communities that will receive 6th, 7th, 8th study MDA and at 9th study visit.

We will test the first three of the above-described hypotheses (about malaria elimination and reduction of general or intestinal inflammation) from the first group of children, i.e., 4–11-month-old infants living in communities receiving their 4th MDA. From these infants, a study nurse will collect a heel-prick blood sample and a flocculated rectal swab before they have received any MDA. They will collect another heel prick blood sample and a stool sample (or rectal swab if stool collection fails) two weeks after the 4th round of MDA has been conducted in their communities. From the collected blood, the nurse will measure hemoglobin and CRP concentration with an on-site reader and store blood on a filter paper for later malaria diagnostics with DNA-amplification. From the stool sample, a laboratory technician will determine calprotectin and alpha-1-antitrypsin concentrations with commercially available ELISA tests. Additional stool and urine samples will be collected and stored for later analyses (on hypotheses 5-7). In the analysis phase, we will compare prevalence of malaria parasitemia, mean blood hemoglobin and CRP concentration and mean fecal neopterin, myeloperoxidase, and alpha-1-antitrypsin concentration in the three study arms, both before and after the first MDA treatment.

From the second and third group of children (12–14-month-olds who have received 3-4 doses of MDA and 6–8-month-olds who have received 2-3 doses of MDA) we will test hypotheses 2-4, i.e., those about broader general and intestinal inflammation and immune function and development. These infants will be invited to a health facility or a central location in a village - after the MDA visit to their household. At the clinic, a study nurse will receive a home collected stool sample or take a rectal swab if stool collection has failed. S/he will also draw a 5 ml blood sample and collect a 10 ml

urine sample, which will be delivered to a nearby study laboratory by a motorcycle messenger. A laboratory technician will measure Hb, separate plasma and white blood cells, aliquot plasma, urine and stool, and store the samples at -80°C freezer. Approximately once a month, the stored samples will be shipped to a central laboratory at CVD-Mali in Bamako, where another laboratory technician will perform the immunological and inflammatory assays. In the analysis phase, we will compare mean plasma concentrations of vaccine antibodies and selected cytokines, thymic output, the ratio of naïve to memory CD4 and CD8 sub-populations in the three study arms. For the azi-biannual group, we will perform an additional exploratory analysis to assess the impact of timing of the azithromycin dose (given at 1-2 and 6-8 months of age vs at 3-5 and 9-11 months of age).

For hypothesis on microbial clearance and reduction of inflammation, we expect to see the largest effect in infants who have not received azithromycin earlier. We also expect that age of the child may be an effect modifier. Hence, we primarily focus on one time-point (fourth MDA) and a wide age-bracket (4–11-month-old infants). The youngest infants (less than 4 months old) will be excluded for logistical reasons as they will not be invited to sample collection for AMR analyses. Since we want to be able to track individual level changes in test variables, we will collect and analyse samples before and after the MDA. The chosen blood analyses can be completed a battery-operated on-site reader from a small volume taken with a heel-prick – and hence we will not need to take any venous samples from these children.

For the hypotheses on immune function and development, we expect to see the largest effect among infants who have received repeated MDA doses. Therefore, we will choose children at a time when there have been at least 4 rounds of MDA in their home villages. At this point, most or all the 12–14-month-old children have received 3-4 MDA doses and 6–8-month-old children have received 2-3 doses. For the immune function and maturity analyses, we will need a larger blood volume and hence we will take it by a venous puncture, at a study clinic after MDA. Since we are interested in a cumulative effect of multiple MDA doses and only want to do group level comparisons, there is no need for paired sample collection.

The age groups have been selected in a way that there will be no more than one venous blood sample per child during the study. From the 6-8-month of children, we will primarily assess immune development and vaccine responses to tetanus and rotaviral vaccines (given before the age of 6 months). From the 12-14-month of children, we will assess immune development and vaccine response to measles vaccine (given usually between 9 and 12 months of age). From both age-groups, we will also assess malaria prevalence by a DNA amplification test. Immune development and infection are best studied among over-6-month-old children, since by this age infants will have lost most of their passive maternal immunity and will be increasingly susceptible for infection, inflammation, dysbiosis, and EED.

Figure 7 summarises analyses planned to be completed in the first phase.

Study question	Biological specimen	Laboratory analysis	Child age	Level of analysis	Timing of lab sample vs MDA	# of given MDA doses	# of children
Malaria clearance	Blood (dried)	P. falciparum DNA amplification	4-11 mo	Individual	Paired (before and 14 d after)	1	1000
Consquence of malaria clearance	Blood (fresh)	Hemoglobin concentration	4-11 mo	Individual	Paired (before and 14 d after)	1	1000
Reduction of systemic inflammation	Blood (fresh)	CRP concentration	4-11 mo	Individual	Paired (before and 14 d after)	1	1000
Reduction of intestinal inflammation	Stool	Calprotectin concentration	4-11 mo	Individual	Paired (before and 14 d after)	1	1000

Figure 8 summarises biological samples planned to be stored from the study participants (in addition to those, stored in the AMR part of the study). These samples will be used address hypotheses 5-7 of the mechanistic studies as well as for any additional hypotheses formulated during the trial. If all samples were collected and aliquoted as planned, there would be a total of 51,800 stored vials, i.e., these samples will fill one -80ºC freezer.

Biological specimen	Child age	MDA or study visit	Timing of lab sample vs MDA	# of given MDA doses	Storage media	Estimated # of aliquots	Volume / aliquot	Storage temperature	# of children
Blood	4-11 mo	4	Before	1-3	Filter paper	2	50 ul	-80°	1000
Stool	4-11 mo	4	Before	1-3	Cryovial	4	1.5 g	-80°	1000
Urine	4-11 mo	4	Before	1-3	Cryovial	3	1.5 ml	-80°	1000
Blood	4-11 mo	4	14 d after	1-3	Filter paper	2	50 ul	-80°	1000
Stool	4-11 mo	4	14 d after	1-3	Cryovial	4	1.5 g	-80°	1000
Urine	4-11 mo	4	14 d after	1-3	Cryovial	3	1.5 ml	-80°	1000
Plasma	6-8 mo	6-9	Before	2-3	Cryovial	4	500 ul	-80°	1350
Peripheral blood mononuclear cells	6-8 mo	6-9	Before	2-3	Cryovial	3	500 ul	-80°	1350
Stool	6-8 mo	6-9	Before	2-3	Cryovial	4	1.5 g	-80°	1350
Urine	6-8 mo	6-9	Before	2-3	Cryovial	3	1.5 ml	-80°	1350
Plasma	12-14 mo	6-9	Before	3-4	Cryovial	4	500 ul	-80°	1350
Peripheral blood mononuclear cells	12-14 mo	6-9	Before	3-4	Cryovial	3	500 ul	-80°	1350
Stool	12-14 mo	6-9	Before	3-4	Cryovial	4	1.5 g	-80°	1350
Urine	12-14 mo	6-9	Before	3-4	Cryovial	3	1.5 ml	-80°	1350

As shown in the figure, we estimate to collect samples from approximately 1,000 infants at the fourth MDA visit and 1,350 thereafter, i.e., approx. 330 - 450 / intervention group.

4.18. Stakeholder perspectives on feasibility, including economic analyses

As previously stated in this protocol (p. 11), in the case of positive clinical results from the LAKANA trial, there is an intention (although not necessarily yet a political commitment) to implement a routine national public health intervention for azithromycin

administration in Mali. By studying demand and supply side determinants, the feasibility sub-study aims at providing comprehensive policy advice to the decision makers on if and how this intervention could be implemented, what can be the bottlenecks and how to go around them.

The feasibility study draws from data collected during the main LAKANA clinical trial, but it will also collect data which is not directly linked to the clinical trial MDA approach in order to explore delivery options which are beyond implementation of the current clinical trial protocol. Going beyond the clinical trial protocol is a necessity as any routine public health intervention model would not have the same type of research component as a clinical trial.

The feasibility study framework breaks down to four research components:

1. Acceptability
2. Equity
3. Health system compatibility
4. Economic analysis based on costing and cost-effectiveness analysis

Figure 9 below depicts framework for the feasibility study and its components.

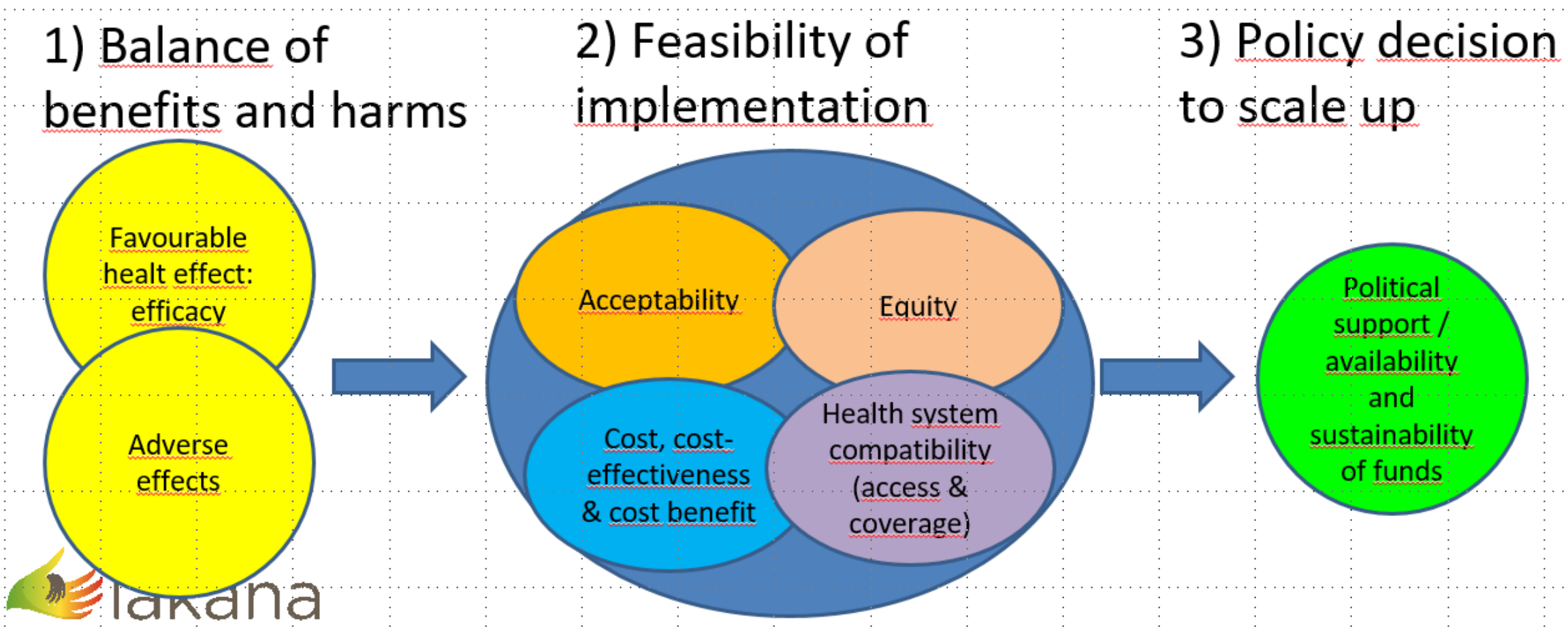

Acceptability

Understanding perspectives and views of various stakeholders on the current LAKANA MDA intervention will form the basis of the acceptability study component. The aim of the acceptability study is to describe how stakeholders conceptualize, perceive and experience the current LAKANA MDA intervention and how they might conceptualize and perceive a future national routine intervention.

Acceptability is a multi-faceted construct that reflects the extent to which stakeholders consider an Azithromycin intervention to be appropriate, based on anticipated or experienced cognitive and emotional responses to the intervention (Sekhon at al 2017). We will confine the present study on health system and household levels (those delivering and receiving the intervention) because of the important role they play. We will focus on local practices and interpretations related to child health, illnesses and well-being and will provide a mostly qualitative analysis of the acceptability of the drug and

the delivery model.

The acceptability study stream will rely on a mixed methods approach: for collecting qualitative data, we will conduct focus-group discussions and semi-structured interviews with families, health workers, *relais*, village chiefs and elders and other key stakeholder groups. Interviews will take place during the trial implementation. Additionally, throughout the intervention, we will conduct in-depth interviews with other stakeholders such as health sector decision makers and politicians. The number of qualitative interviews needed will be based on data saturation. Typically, the total number would be around 50 interviews. We will contextualize the qualitative data using quantitative data collected from all participating households (household socio-economic status, location of residence, family composition and other background variables).
We will also collect quantitative data from households through a questionnaire which will be administered in the 59 sub-study villages. The questionnaires will cover questions related to acceptability but also to the economic analysis (see below)

The CVD social science team in collaboration with Point Sud research unit will conduct qualitative interviews in the vernacular (Bambara), transcribed and translated into French. The CVD social science team, together with Tampere University feasibility team will read the interviews immediately after they have been conducted and add new themes to cover topics that remain unanswered to the interview guide. The analysis of the qualitative data will be done using a multipronged approach including thematic and discourse analysis. The analysis might include use of a software such as Atlas.ti. We will analyse quantitative data for proportions and means using Stata software.

<u>Equity</u>
The drugs will be distributed in a research context aiming to determine efficacy of azithromycin. By collecting various types of background information from the study participants and assessing if the background variables (such as poverty or household location), we will be able to assess the equity of impact of this intervention. However, since there will be no data on those whom the study team does not reach, villages that refuse participation, or areas that are excluded from the study due to security or other issues, we will not be able to quantitatively measure equity of access to the intervention. Instead, we will assess equity of access indirectly, using qualitative interviews among stakeholder groups. We will use snowball sampling for selecting the interviewees, but preliminary, we plan to include representatives of multilateral organizations, funding partners, national Ministry of Health (MOH) teams, district MOH teams, volunteer rural health workers, and community members participating in MDA campaigns. In this sub-study, we aim to explore the possible bottlenecks of achieving effective coverage and ways to overcome the difficulties in implementation.

We will consider the village location and accessibility as an equital aspect: we will treat the village's Strategie Avancée status as a potential effect modifier of azithromycin MDA. Additionally, we will conduct a survey amongst the field workers to obtain a subjective assessment on how difficult the villages are to reach as a health worker. The results will be assessed in the context of whether the district or health area has a system in place to take the remote and difficult locations into account in terms of arranging health care.

Health system compatibility

The health system compatibility study component aims at answering the study question of: what are the health system compatibility parameters for different implementation scenarios for an Azithromycin public health intervention in the context of Mali?

This study stream will include questions on the transferability of the LAKANA intervention to a scaled-up national intervention, but it will go beyond the LAKANA trial implementation by exploring other possible implementation models. The different possible implementation models are assessed through supply side elements (health system building blocks) in the Malian context. The implementation models will detail the people and institutions involved in each model and the process of implementation of each model.

The health system compatibility study stream will be based on literature and observations on the Malian health system and on interviews with key stakeholders, such as health system researchers, health policy makers, health workers at different levels of the system. The health system compatibility study stream will also draw from lessons learned in implementing the LAKANA trial.

The theoretical framework for the health system compatibility study stream relies on implementation research / science. Bauer et al (2015) have defined implementation science as “The study of methods to promote the adoption and integration of evidence-based practices, policies, research findings and evidence into healthcare policy and practice.”

Economic analysis based on costing and cost-effectiveness analysis

The economic analysis study component aims at answering the study question of: what is the cost of the different implementation models and what are the differences in cost-effectiveness between the different models? The costing exercise will be based on an ingredients approach in which all the different inputs or ingredients that are needed to produce one unit of the intervention need to be costed trough market prices or prices derived from competitive market prices. All the prices will be translated into one single monetary unit.

The costs include in the costing are direct, indirect and opportunity costs. The direct costs will be mostly derived and modelled from the LAKANA trial costs with the deduction of research costs. These costs will be modelled to represent the base line MDA approach. For the other implementation models the costing model will require further adjustments according to how the models will be defined. This adjustment can only be done after the implementation models have been developed.

The costing exercise will also include household level opportunity cost. This equates to the indirect cost paid by the participants, more precisely the care givers bringing their children to receive the Azithromycin intervention. To calculate the household level cost the intervention logic (as described above) will need to cover also the household perspective, i.e., what is required from the household (in terms of time and indirect payments to reach the point of service) to receive the service.

The effectiveness will be determined by the trial outcome – both the primary outcome of mortality and the secondary outcomes of morbidity and possible AMR effects. The effectiveness can be expressed in different units such as deaths averted, or disability adjusted life years (DALYs).

Theoretical framework for the costing and cost-effectiveness analysis are based on standard economic evaluation methods – with focus on theoretical frameworks covering economic evaluation of public health programmes (WHO 2004; NICE International, 2014; Sohn et al., 2020).

4.19. Strategie Avancée and field workers' assessment of hard-to-reach areas

Villages under Strategie Avancée will be identified by the local field supervisors from the records maintained by the local districts and health areas. The Stategie Avancée status information will be appended to the study data prior to the analysis by the study data manager.

We will conduct a survey amongst the trial data collection staff to obtain a subjective assessment on what is the geographical accessibility of a village. The overarching aims of the survey for the data collectors would be to develop an instrument to assess the hardness-to-reach from the point of view of data collectors or health care workers going to the locations, and to evaluate whether hardness-to-reach modify the effectiveness of LAKANA intervention. The survey will be implemented as an electronic questionnaire and the survey will be anonymous: no staff name or position will be recorded.

The participants will be approached by their field supervisors with an announcement of an invitation to fill the questionnaire based on their own experiences. Filling the questionnaire will be voluntary, but they will be informed on the benefits of what such information can provide: insight on their challenges during the data collection, an improved viewpoint on the collected trial data with hardness-to-reach as a predictor or effect modifier on the trial outcomes, and information that could improve the planning of future interventions that could help even the most remote communities.

The participants will be asked to fill the questionnaire before synchronizing the tablets for data collection, either at the beginning of the day or at the end of the day, at their convenience. The questions will be in French language, and at the beginning of the questionnaire, there will a brief introduction and instructions on filling the electric form.

The form will consist of 12 questions for a single village. Each field worker will fill the form for 10 villages. The questions will be multiple choice questions and filling the form for one village will take approximately 5 minutes.

The expected number of answers will be 75% of the available field workers. The field supervisors will be reminded to ask the field workers to fill the form until 75% is reached, or until the field supervisors report that there are no more field workers who are willing to fill the form. As the data collectors enter villages in teams, there are multiple data

collectors who have experience from a single village. Each village should be assessed by the full team of field workers, that is, each village should have at minimum two assessment by separate field workers.

There will be multiple survey rounds: the survey will be tested with 5 data collectors to assess the workload filling the forms for multiple villages, and to assess the data quality. If the data quality suffers from the workload of filling the questionnaire for many villages, the length of the electronic data capture form will be reduced by allowing the field workers to continue form filling at the later time.

4.20. Data collection on trial safety

We will monitor intervention safety in LAKANA with two approaches:

1. Passive surveillance for the incidence of serious adverse events (SAE) within 14 days of study drug administration, in the whole study area
2. Active surveillance for the incidence of SAEs and adverse events (AE) within 14 days of study drug administration, in the 60 villages selected for more detailed data collection on outcomes other than mortality.

The passive surveillance will be done through health facilities in the study area. Whilst an MDA round is being implemented in any location and and for two weeks thereafter, a study coordinator will visit or call the local health center on each week-day and record any suspected SAEs that have been notified among 1-11 month old infants at the facility. Caregivers of infants who receive study drugs are also adviced to notify study team members if the infant is hospitalised within two weeks of the MDA.

The following events will be considered SAEs, if they occur within 14 days of study drug administration: Death, life-threatening event, hospitalization, and other serious events as judged by a study physician.

Active surveillance for SAE and AE will be completed for participants in the 60 clusters selected for more detailed data collection on a number of secondary outcomes and other variables. Infants aged 4-11 month old will be invited to the health facilities or central location in a village two weeks after the 4th round of MDA has been conducted in their communities. At this visit, a study nurse or another trained study team member will also interview a caregiver about symptoms that the infant has experienced after the azithromycin MDA and that may possibly be related to it.

4.21. Group allocation and trial code managament

Participating study clusters (villages) will be randomly allocated into the three intervention groups at 3:2:4 ratio (control: azi-quarterly: azi-biannual), in public allocation events where village representatives blindly pull lottery tickets out of a container. The randomization will be stratified by cluster size (below or above 100

infants / cluster) and by district, i.e., each participating district will have its own randomization event. The randomization lists (trial code) will be stored electronically, in a password protected file, by a statistician not involved in trial implementation, the data management consulting company, and by the Chairman of the trial's data safety and monitoring board (DSMB).

The trial code will be opened when all trial data for the 24-month follow-up visit have been entered into a computer database and data accuracy has been verified. To facilitate an analysis on mortality and other efficacy outcomes whilst follow-up for AMR is still going on, a statistician will temporarily break the code, add group information to a database containing the efficacy outcomes and then recode participant and cluster codes into new identifiers that cannot be linked to the actual identification. With this approach, the statistician can then share a full database with researchers analysing the efficacy outcomes, whilst others continuing the data collection of AMR outcomes will remain blinded to the group code.

4.22. Methods for protecting against other sources of bias

Randomisation into the trial, group allocation, and distribution of study drugs will be done by personnel not participating in the outcome evaluation.

The interventions will be double-masked, i.e., the study drugs will look and taste identical and coded only with a letter code.

To ensure adherence, all study drugs are given under direct observation.

4.23. Withdrawal from participation after enrollment

The enrolled household representatives can decide to discontinue the study participation or decline provision of study drug to an infant at any point. If known, the reason for household withdrawal or study drug denial will be indicated in electronic Case Report Forms. The participants can, however, withdraw their consent or decline study provision to a child without giving any reason for their withdrawal.

Individuals experiencing a serious adverse event that is likely to be related to the trial intervention are withdrawn from further study drug provision by the investigators (but will be included in the follow-up and analysis).

Withdrawn or discontinuing participants will not be replaced by others, i.e. possible dropouts will not influence the sample size, once the study has started. The study team will compare the baseline characteristics of discontinuing households to those who do not discontinue, in order to assess the possibility of follow-up bias.

4.24. Other treatments to trial participants

During and after the trial, members of all participating households (i.e., those completing the planned follow-up period as well as those withdrawing at some point) will receive standard preventive and curative health services, as recommended and provided by the national health system. SMC with amodiaquine and sulfadoxine-pyrimethamine will be offered through the national health service to all 3–59-month-old children on a monthly basis between July and October-November in each year.

At the end of the trial, if the analysis suggests a mortality reduction by azithromycin, all 1-11 month (29-364 days) old infants in the participating households will be offered a 20 mg / kg dose of azithromycin. If deemed appropriate thereafter, the study team will work with Malian Ministry of Health to roll-out a longer-term azithromycin MDA program at the trial site and elsewhere in Mali.

4.25. Co-enrollment guidelines

LAKANA trial participants are encouraged not to enrol in other clinical trials using antibiotics before the end of the LAKANA follow-up period. However, participation in or enrollment to another trial is not an exclusion criterion, i.e. child can be included in the trial and receive study drug MDA if s/he was participating also in another trial.

4.26. Storage of biological samples

All collected biological samples will originally be shipped daily to study laboratory in Kita, where they will be processed and stored at a project freezer (-80°C). On a monthly

basis, the samples will be shipped to a main laboratory at CVD-Mali in Bamako and stored at -80°C. All biological samples will be aliquoted in small volumes, to allow appropriate vials to be easily shipped to various collaborators, where they will be stored until analysis. Chapters 4.15 and 4.16 gives details of the stored aliquots.

4.27. Data recording and management

All data will be captured electronically, using a custom-made data entry software installed on personal smartphones or small tablet computers. The exact software is yet to be decided but the data management approach will involve offline data collection, mostly at participant's home. Data transmission to a cloud-based server will occur in real-time from households in the field or daily from cluster centers, using a 3G broadband connection. All server-based data will be backed up to another location daily.

Access to the tablet computers and the cloud-based data will be username and password protected. During transmission and at the cloud, data will be encrypted to ensure data privacy. The tablet computers will be stored and charged overnight at locked study offices that will have safety and electricity.

Error and inconsistency checks will be automated in the software. A data manager will review new data entries and full data report on a weekly basis (daily at the beginning of data collection) and make corrections where appropriate, to ensure data integrity.

All documentation regarding the participants, including the laboratory samples, source data and the Case Report Forms, will be identified in the database with appropriate participant codes. The names will only appear on informed consent forms and a separate coding list. A minimal set of identifying information, necessary for the implementation of the trial, will be stored in an encrypted form on the data collection devises and in a separate encrypted database. Access to the identifying information will be limited to authorised data collectors with a secure username and password.

The investigators shall maintain electronic records of study drug receipts and transfer logs and electronic copies of the Case Report Forms and regulatory documents (informed consents, ethical approval) for 5 years after the end of the study, as advised by local authorities. All records will be kept in a secure place. Clinical information will not be released without written permission of the subject, except as necessary for monitoring.

Selected data (participant's name, sex, date of birth, date of last visit, home village, household identification number, enrollment date and enrollment number) will be summarised in an electronic register. The registers are stored in encrypted form and accessible only with an authorised username and password in the data collectors tablet computers (to facilitate participant identification) and a cloud-based folder. Data from this register may be given to selected members of the research teams and authorities guiding health research in Mali or Finland.

After the trial completion, the study team will place the study database and respective metadata publicly available, at a suitable non-profit internet-site (such as ClinEpiDB).

The study team will contract an external partner (RTI International) for the development of the data entry and management system support for its maintenance. After the initial 14 months, i.e. when the system is running and enrollment almost completed, the study team will assume full responsibility of data management.

4.28. Pilot study

There will be a 3-month pilot phase, during which the team will hire and train study staff and test standard operating procedures, data collection forms, and data management. Many practical arrangements and standard operating procedures (SOPs) will be adopted from earlier ABCD and SANTE -trials, also testing various aspects of azithromycin impacts on children and currently being carried out in Mali, with the same Malian co-PI as in LAKANA trial.

Mortality data from the first two 3-month intervals, from the villages that will be enrolled in the first week of trial activities, will be treated as pilot data and not included in the main database for trial analysis.

4.29. Quality assurance and control

Internal quality assurance is implemented using standard operating procedures (SOPs) and weekly internal monitoring. The team in Mali will employ 1-3 quality assurance officers, who will regularly work with the staff on quality assurance procedures. For external monitoring, the team will contract a clinical research organization that will complete a pre-site trial assessment, quarterly monitoring visits during the trial implementation, and data collection closure visit. Additionally, Tampere University staff members will conduct bi-annual internal monitoring visits to the study sites.

If requested, the investigators will make study documents (e.g., consent forms, drug distribution forms, case report forms) and pertinent hospital or clinic records readily available for inspection by the site auditors for confirmation of the study data if the study site is selected for auditing by the Comité D'Ethique de la FMPOS (Faculte de Medicine, de Pharmacie et D’Odonto-Stomatologie, Universite des Sciences, des Techniques et des Technologies de Bamako).

4.30. Originally planned time schedule

(not updated after version 1.0)

In total, the project will take 4 years and 2 months. The detailed time schedule is below.

November - December, 2019	Finalising research plan<br>Approval of the plan by appropriate institutional

	review boards (IRB)<br>Developing SOPs and planning practical arrangements<br>Developing data entry and management system
January – March 2020	Finalising data entry and management system<br>Staff recruitment and training<br>Obtaining study drugs
April 2020 – March 2021	First study visits at all study villages
April 2021 – December 2021	Completion of 4 MDA rounds in all villages
January 2022	Interim analysis. Dropping placebo if justified
January 2022 – September 2022	Completion of all main study follow-up visits
October 2022 – December 2022	Data cleaning and analysis
January 2023 – December 2023	Write-up of main study results (mortality, morbidity, and growth outcomes<br>Local results dissemination on main outcomes<br>Completion of AMR follow-up visits and analyses, internationl and local results dissemination on AMR outcomes<br>Continued feasibility analyses<br>Roll-out of azithromycin MDA program, if indicated

Figure 10 shows a graphic illustration of trial schedule. Planned interim analysis is marked in transparent red color. To shorten the trial period by six months, the last enrolled villages will be followed up only for 7 MDA rounds (30% of villages) or 6 MDA rounds (20% of villages).

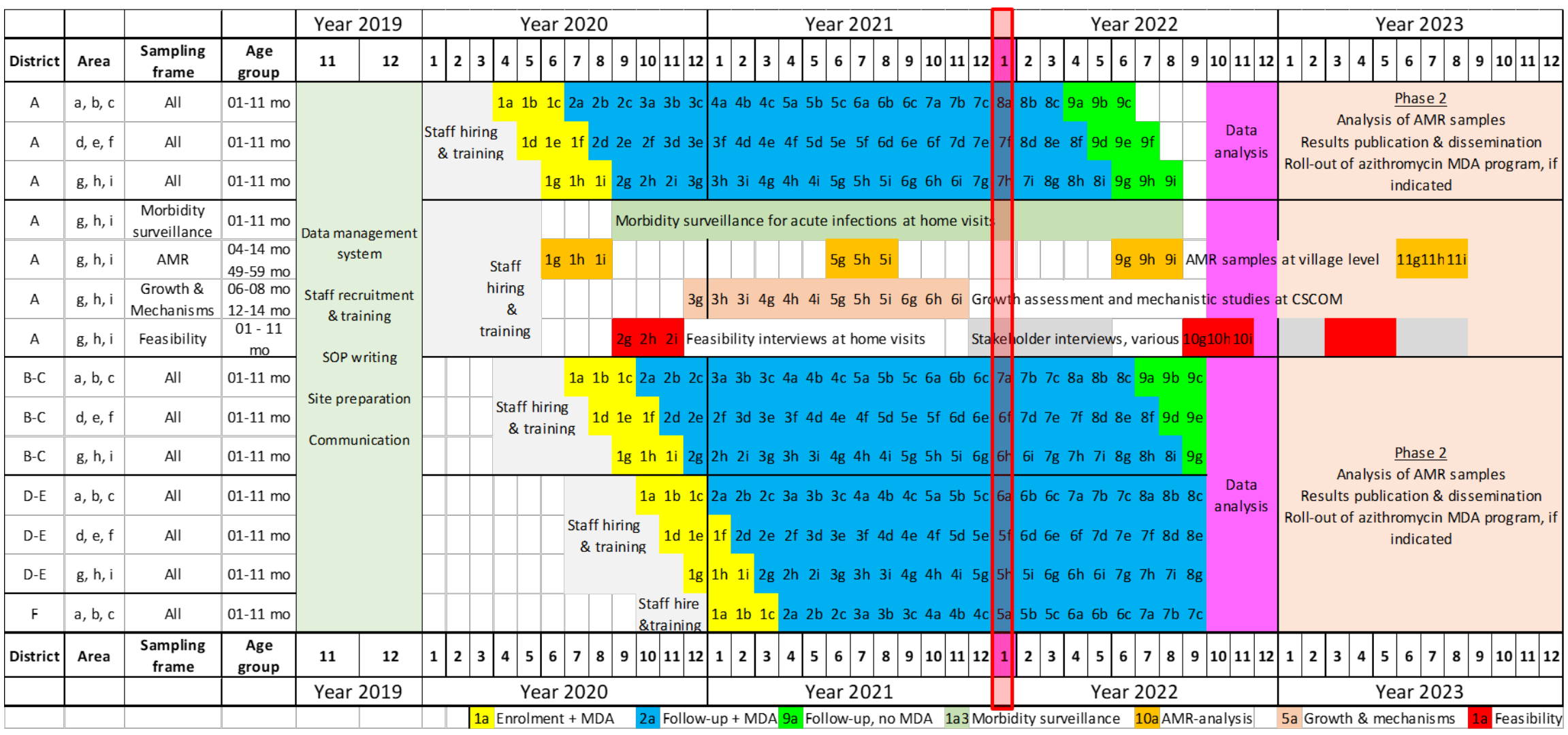

5. Statistical considerations and data analysis

5.01. Specific hypotheses

The main study hypotheses in terms of mortality effect are the following:

1. Biannual azithromycin MDA to 1-11 month (29-364 days) old infants reduces their mortality
2. Quarterly azithromycin MDA to 1-11 month (29-364 days) old infants reduces their mortality
3. Quarterly azithromycin MDA has a bigger mortality effect than biannual MDA

The study hypotheses in terms of other health effects are the following:

4. Quarterly azithromycin MDA to 1-11 month (29-364 days) old infants reduces their 14-day period prevalence of fever with respiratory symptoms (ARI), fever without respiratory symptoms (malaria), and diarrhea
    a. Separately, there is a similar hypothesis on the effect of biannual azi MDA
5. Quarterly azithromycin MDA to 1-11 month (29-364 days) old infants reduces their health facility visits for ARI, malaria, diarrhea, and other conditions
    a. Separately, there is a similar hypothesis on the effect of biannual azi MDA
6. Quarterly azithromycin MDA to 1-11 month (29-364 days) old infants improves their length gain, and nutritional status (MUAC, WHZ)?
    a. Separately, there is a similar hypothesis on the effect of biannual azi MDA

The study hypotheses in terms of intervention health effects on AMR are the following:

7. Quarterly azithromycin MDA to 1-11 month (29-364 days) old infants does not cause a sustained increase in the prevalence of phenotypic azithromycin resistance among *S. pneumoniae* or *E. coli* strains isolated from their nasopharyngeal secretions or stools.
    a. Measured at 12 months after the intervention has stopped
    b. Separately, there is a similar hypothesis on the effect of biannual azi MDA
8. Quarterly azithromycin MDA to 1-11 month (29-364 days) old infants does not increase the prevalence of azithromycin resistance among *S. pneumoniae* or *E. coli* strains isolated from 49–59-month-old children, who live in the same Malian communities but have not received azithromycin MDA?
    a. Measured when the intervention has lasted for 12-18 months
    b. Separately, there is a similar hypothesis on the effect of biannual azi MDA
9. Quarterly azithromycin MDA to 1-11 month (29-364 days) old infants does not cause a sustained increase in the prevalence of azithromycin resistance genes in their intestinal microbiota.
    a. Measured when the intervention has lasted for 12-18 months
    b. Separately, there is a similar hypothesis on the effect of biannual azi MDA
10. Quarterly azithromycin MDA to 1-11 month (29-364 days) old infants does not increase carriage of azithromycin resistance bacteria in treated infants 1 year after

their treatment has stopped, who live in a village where MDA intervention has also ceased.
   a. Measured after the intervention for individual child has stopped for 1 year
   b. Separately, there is a similar hypothesis on the effect of biannual azi MDA
11. Quarterly azithromycin MDA to 1-11 month (29-364 days) old infants does not increase carriage of azithromycin resistance bacteria in treated infants 1year after their treatment has stopped, who live in a village where MDA intervention has continued.
    a. Measured after the intervention for individual child has stopped for 1 year
    b. Separately, there is a similar hypothesis on the effect of biannual azi MDA.

The study hypotheses in terms of azithromycin mechanism of action are the following:

12. Azithromycin MDA reduces malaria prevalence and parasite density, and improves mean blood hemoglobin concentration among 4–11-month-old infants, within 14 days of the MDA
    a. For these analyses, the two azithromycin can be combined
13. Azithromycin MDA reduces general inflammation (CRP) among 4–11-month-old infants, within 14 days of the MDA
    a. For these analyses, the two azithromycin can be combined
14. Azithromycin MDA reduces intestinal inflammation (fecal neopterin, myeloperoxidase, and alpha-1-antitrypsin concentrations) among 4–11-month-old infants, within 14 days of the MDA
    a. For these analyses, the two azithromycin can be combined

There is no predefined hypothesis about the impact of biannual or quarterly azithromycin MDA to 1–11-month-old infants on mortality among 12–59-month-old children living in the same communities, but we will measure group-level differences on this as exploratory analysis.

There are also no predefined hypotheses about the feasibility, acceptability, or equity of the azithromycin MDA intervention.

5.02. Analytical approach

The study team will express mortality in the three groups as number of deaths per 1000 person-years at risk (deaths/PYR) and AMR as the proportion of *E. coli* or *S. pneumoniae* isolates that have reduced susceptibility (R or I category) towards azithromycin. For pairwise comparisons between any of the two groups, the team will calculate incidence rate ratio and its 95% confidence interval (CI) for mortality and risk ratio and its 95% CI for AMR prevalence at 12 and 24 months after the onset of the azithromycin MDA intervention.

As an exploratory analysis, the team will also assess mortality (deaths / 1000 years at risk) among children who were 12–59-month-old at the time of the previous study drug administration in their village of residence. There is no predefined hypothesis for this

analysis, but it can provide information about possible herd protection generated through targeted azithromycin MDA.

Mixed-effect Poisson regression model will be used to estimate the intervention effects on mortality, with random intercepts for the clusters. For twins, no adjustment for inter-dependence will be made, i.e. we plan to analyse the data assuming independence between infants from the same mother. This is because our previous methodological research has demonstrated that analytic models that assume independence are robust in situations where cluster size is small and only a few of the analysable units are clustered (such as 1-2% twin rate, Xu et al., 2014). As per the closed-testing method for controlling multiplicity arising from multiple group comparisons, global null hypothesis of mortality in all three groups being the same will be tested at 5% significance level. Pairwise null hypothesis can be rejected only if the global null hypothesis has been rejected. Pairwise incidence rate ratio and corresponding 95% confidence intervals will be estimated.

For the main analysis, we will analyse the mortality data of infants/children who receive azithromycin/placebo at the age of 1-11 months (29-364 days) at the beginning of each 3-month period. Some of the person-time and deaths may occur at age 12-14 months during the 3-month periods. This is consistent with the study aim to assess the effect of MDA giving at the age of 1-11 months (29-364 days). As a secondary analysis, we will use the Lexis expansion approach to partition the follow-up time and deaths into intervals of 1-5, 6-11, and 12-14 months of age (and also 1-11 and 12-14 months) and cross-classified with intervals defined according to the 3-month periods. This allows for more precise estimation of age-specific intervention effects and more precise adjustment for the time period effects.

For AMR, the team will calculate the risk differences (and their two-sided 95% CIs) for AMR prevalence in azi-quarterly and azi-biannual groups as compared to the control group, separately. If the entire 95% CI of the risk difference of a MDA regime is below a predefined non-inferiority margin of 0.1 (meaning less than 10%-point absolute increase in AMR prevalence), the sample findings will be considered supportive of the hypothesis of no clinically significant increase in AMR prevalence for that regime.

For morbidity analyses, we will calculate 14-day period prevalence of fever with respiratory symptoms (ARI), fever without respiratory symptoms (malaria), and diarrhea in the three intervention arms and do a three-group comparison using likelihood ratio tests. The latter will be obtained from mixed-effects logistic regression models, which accounts for the clustering of observations. If the test shows a statistically significant difference ($p<0.05$) between the three groups, we will proceed into three pair-wise comparisons, providing relative risks (RR) and their 95% Cis. Hypothesis testing in these pairwise comparisons will be done with likelihood ratio test obtained from mixed-effects logistic regression models. If the p-value is <0.05, the results will be considered supportive of the trial hypothesis.

For growth analysis, we will calculate anthropometric indices LAZ (length for age Z-score), WAZ (weight for age Z-score), WLZ (weight for length Z-score), and MUAC-Z (mid upper arm Z-score) for each participant as described in chapter 4.10. We will then calculate mean (SD) group values for each variable. In analyzing differences between groups, controlling for multiplicity will be conducted as per the closed-testing procedure,

by testing a global null hypothesis at 5% significance level. If the global null hypothesis is rejected ($p<0.05$), three pairwise comparisons will be carried out. The differences between the three groups and their 95% CIs will be obtained from mixed-effects regression models, that account for the clustering of observations. The global p-value will also be obtained from these models.

For mechanism of action analyses, we will calculate the prevalence of malaria parasitemia as well as mean plasma CRP, blood hemoglobin and other biomarker concentration before and at two weeks after the MDA. We will then test a global hypothesis about differences between the groups using mixed-effects models, which account for clustering, for continuous variables and proportions, respectively with different, suitable link functions. If the global test shows a statistically significant difference ($p<0.05$) between the three groups, we will proceed into three pair-wise comparisons, providing the difference (95% CI) in means or proportions. If the p-value obtained from mixed-effects models is <0.05, the results will be considered supportive of the trial hypothesis.

All children will be analysed in the group into which they were initially randomized, i.e. on an intention-to-treat basis.

5.03. Frequency of analyses

The randomisation code will be broken after the ninth study visit has been completed in all clusters (24 months after the last cluster was included in the study) and the database has been reviewed for data accuracy. The main analysis will be done at this point. The analysis will first be completed using letter-coded intervention names (A, B, C). Once the principal investigators have reviewed the analysis results and agreed that there is no need for further data or analysis edits, the code linking specific interventions to the letter codes will be opened.

As described in chapter 4.13, one interim efficacy analysis will be completed when 60% of the planned post-MDA 3-month intervals have been completed. If there is a statistically significant mortality difference between both of the azithromycin groups and the placebo group but not between the two azithromycin groups, the placebo arm will be discontinued, and trial completed with two arms only. If there is also a statistically significant difference between the two azithromycin groups, the trial will be stopped, and the team will offer to work with Malian Ministry of Health to provide azithromycin MDA in the trial site and two other regions of Mali.

Concomitantly with the interim efficacy analysis, there will be an interim safety analysis.

Additional safety or efficacy analyses will be completed if requested by the study DSMB.

5.04. Sample size and its justification

The sample size will be in total 1150 clusters (villages), of which we will use all for the primary analyses on mortality and secondary and tertiary samples for the secondary outcome analyses. At each round of home visits, each cluster is expected to contain on average 39 infants, of whom 34 will be found and given MDA and 31 found in the subsequent visit, thus providing data to the mortality analysis. Thus, approximately 35,650 infants will provide follow-up data at first six home visit rounds, 28,520 on the 7th round and 17,820 on the 8th round. In total the trial will result in approximately 260,200 analysable time intervals.

The sample size is based on the following assumptions:

1. Mortality among 1-11 month (29-364 days) old children in control group of 20 deaths / 1000 PYR (20% lower than in the latest DHS)
2. Mortality in the azi-biannual intervention group 16/1000 (20% relative reduction)
3. Mortality in the azi-quarterly intervention group 12 / 1000 (25% lower than control, 40% lower than azi-biannual)
4. 2-year intervention with 8 quarterly cycles of MDA for 50% of clusters, 7 rounds for 30%, 6 rounds for 20%), due to staggered entry but equal stopping time
5. One interim analysis planned, when 60% of the planned 3-month time intervals have been completed
6. One-sided 2.5% type 1 error, controlling of multiple group comparisons by the closed testing procedure and multiple looks by the Peto method.
7. Coefficient of variation (k=sd/mean) of 0.1 in mortality among clusters.
8. Unequal number of infants per cluster: on average 22 infants in a small village and 70 in a big village
9. Unequal ratio of clusters. Control vs azi-biannual vs azi-quarterly = 3 : 4 : 2

Using the power-by-simulation approach stratified by size of the clusters (dichotomized with cut-off point of 100 infants per cluster), it is determined that a sample size of 1150 clusters with on average 31 analysable infants per cluster / time interval, there will be approximately 89% power for testing the hypothesis that biannual azithromycin MDA will reduce mortality, >99% power for testing the hypothesis that quarterly azithromycin MDA will reduce mortality and 80% power for testing a hypothesis that quarterly azithromycin MDA will reduce mortality more than biannual azithromycin MDA.

The relative risk reduction (RRR) of 20% in the azi-biannual group is modelled on the MORDOR-trial, in which the point-estimate for mortality reduction among under-one-year old infants was 25% (in all the study countries) (Keenan et al, 2018). We estimate this reduction be slightly lower in Mali, because of the seasonal malaria chemoprevention that will be offered to 3-11-month-old infants in the Mali but was not practiced at the MORDOR sites. The 40% RRR for the azi-quarterly group is also modelled on MORDOR result indicating that in that sample practically all the mortality effect was concentrated in the first 3 months after the MDA (Porco et al, 2019).

For the AMR data collection, the sample size will be 60 clusters or 1,350 children per time-point. This number will be tested from 4–14-month-old children at 12 months after the intervention has stopped and from 49–59-month-old children when 8 rounds of MDA have been completed. At the three other time points, we will test 450 children (taken

randomly from 20 clusters / arm). In total, this will mean 5,400 AMR-analyses for *S. pneumoniae* and the same number for *E. coli*.

The AMR sub-sample size is based on the following assumptions

1. 95% *E. coli* and 65% *S. pneumoniae recovery* rate from the collected samples
2. AMR prevalence of 12% in the control group for *E. coli* and 6% for *S. pneumoniae*. These figures come from analysis of azithromycin resistance in 50 *E. coli* and 50 *S. pneumoniae* samples from Mali in the so called ABCD trial.
3. Non-inferiority margin of 10%-points in AMR prevalence
4. 80% power, one-sided 2.5% type 1 error rate for each pairwise comparison against placebo control
5. Coefficient of variation of 0.3 in AMR among clusters.

For the analyses on azithromycin mechanism, we are using a convenience sample, i.e. all available children in the intended age-brackets (and avoiding repeated venous blood collection from the same children), in the study clusters where the AMR work is done. The sample size of 1,000 – 1,350 / analysis, or a mean of 333 - 450 / intervention arm, will provide these analyses 80% power at 5% two-sided type 1 error rate to detect an effect size of 0.25 – 0.30, assuming an intra-cluster correlation coefficient of 0.03 and a design effect of 1.6 to 2.

For intra-household analyses there are no pre-set hypotheses, but the data will be used for descriptive purposes.

5.05. Subgroup analyses

For hypothesis generating purposes, we plan to carry out exploratory tests for interaction between the MDA intervention and the selected baseline variables, using the mortality and growth outcomes. Tests of hypotheses concerning intervention effects in these subgroups will be performed only if the interaction tests give statistically significant results ($p<0.1$).

The following variables would be assessed potential effect modifiers

1. Infant age at the time of MDA (1-5 months vs 6-11 months)
2. Infant weight-for-age, length-for-age, weight-for-length at the time of MDA
3. Infant sex
4. Season of MDA dosing (malaria versus non malaria season) and SMC implementation in village (SMC given versus SMC not given)
5. Cluster level coverage of SMC
6. Cluster level baseline mortality (established at first census)
7. Cluster and individual level coverage and number of administered azithromycin MDA doses
8. District of residence
9. Distance from the nearest health facility
10. Household asset or income index
11. Household WASH index
12. National outreach strategy category (standard/advanced)

13. Hard-to-reach village status assessed by the field workers

6. Ethical considerations

6.01. General principles

The trial will be performed according to Good Clinical Practice guidelines (ICH-GCP) and it will adhere to the principles of Helsinki declaration and regulatory guidelines in Mali. Trial recruitment shall not begin before the ethics committee in Mali (Comité D'Ethique de l'USTTB (Université des Sciences, des Techniques et des Technologies de Bamako) has given a favourable statement on it. An ethics committee in Finland has no legal mandate to authorize trials abroad, but a favourable opinion from one (Pirkanmaan Sairaanhoitopiirin eettinen toimikunta) will be required for TAU researchers to participate in the project.

6.02. Informed consent

Permissions for trial implementation will be sought on three levels at the study site: Village (cluster), household, and individual child.

For village enrollment, members of the study team will visit village leadership, give them oral and written information about the trial (Appendix 1a) and request a permission to make an information campaign and perform study activities in the selected village. The response will be given verbally and documented electronically in a study database (Appendix 1b).

If a village level permission is granted, the study team will run an information campaign about the upcoming trial in the village and its nearby health facilities. After one week of general information campaign, the team will start household visits.

A team consisting of a community volunteer and a study team member will conduct the household visits. At each household visit, the team members will inform the heads of household and other household members about the purpose and processes of the trial, provide a written documentation about it (Appendix 2a) and request a consent (Appendix 2b) to visit the household and collect trial data on a total of nine times over a period of two years. The response will be given verbally and documented electronically in a study database. If the person giving consent is illiterate, study personnel will ask her / him to invite an impartial witness to verify the consent. The name and home village of the impartial witness will be recorded in the consent database.

Since the local language (Bambara) is mostly not a written language, many people may not be able to read it. For standardisation purposes, the team will write and hand out the participant information and consent form texts both in French and in Bambara. From the

Bambara text, the trial team will make an audiotape, so that the message can be given in a standardised manner by different data collectors. For information purposes, the team will also make a picture booklet as well as Bambara language videos describing the study procedures.

During the household visits, the community volunteer and the data collector request a permission to weight and provide study drugs to all 1-11- month-old children (age 29-364 days). Information about the purpose and process for this treatment is provided verbally and the response is also provided verbally and documented electronically in a study database (Appendix 3).

At selected visits, sub-samples of children are invited for further data collection on secondary outcomes and other relevant data at a nearby health center or at central location in a village. Participant information and consent forms for the secondary outcome data collection are shown as Appendices 4a (participant information) and 4b (consent form).

6.03. Possible risks and benefits to study participants

Azithromycin stimulates smooth muscle contraction and may cause mild abdominal discomfort, vomiting, or diarrhea in some children. In earlier MDA trials with single-dose azithromycin use in infants, this has not been frequently reported by caregivers (Porco et al., 2009; Keenan et al., 2018; Chandramohan et al., 2019). In several studies, where azithromycin treatment has been compared to other antibiotics, the incidence of gastrointestinal side-effects has been smaller in the azithromycin than the comparison groups (Roord et al., 1996; Ferwerda et all., 2001; Langly at al., 2004). Hence, we don't expect much of this problem for LAKANA participants.

Among newborns, there is one theoretical safety concern, because of which neonates will be excluded from study drug administration in the LAKANA trial. In this age group, azithromycin may induce pyloric stenosis, an obstruction of gastric outlet that may require surgical intervention. In some population analyses, an increased risk for this rare condition has been associated with azithromycin use among under-14-day-old neonates, but not older children (Eberly et al., 2015). Since it is not known if the increased pyloric stenosis risk has been eliminated by one month of post-natal age also among preterm babies and since we will not be able to ascertain the duration of pregnancy at birth, all infants whose weight is below 3.0 kg will be excluded from the trial (3.0 kg is the 3rd weight centile among healthy 1 month olds, according to WHO growth charts).

Besides the possibly increased risk of the rare pyloric stenosis in newborns and possibly causing abdominal discomfort, azithromycin has an excellent safety profile and is not expected to pose health risks to LAKANA participants. In the United States, azithromycin has been FDA approved for use only for children who are six-months or older, but there are numerous scientific reports and a systematic review that document safety also for younger infants (Pinto et al., 2012; McCallum et al., 2013; Beigelman et al., 2015; Smith et al., 2015). There are also several official US guidelines that recommend azithromycin use for under-six month old infants (American Academy of

Pediatrics, 2012; Centers for Disease Control, 2019). Also, in trachoma control programs, azithromycin has been used for post-neonatal infants for 20 years without safety concerns.

All participants will benefit from trial participation in terms of repeated home visits, during which caregivers may ask health-related questions from the study personnel. The study staff making home visits will not be medically trained and they will be instructed not to provide direct medical advice. However, if needed, they will facilitate referral to a local health facility. If a further referral to a hospital is required, the study team will assist in transfer where necessary. For children participating in the mechanistic substudy for whom blood samples are collected and Hb and CRP levels measured on site: on the first visit, if a child is asymptomatic or has mild respiratory symptoms, and the CRP is above or Hb is below a critical threshold based on reference ranges from the Mali national and local guidelines in use at that time, the child will be given the study drug and then actively referred to the nearest health clinic. Transportation will be ensured. On the second visit two weeks later, if the Hb reaches a critical threshold based on reference ranges from the Mali national and local guidelines in use at that time, the child will be actively referred to the nearest health clinic and transportation will be ensured. Upon referral, a paper copy of an instruction leaflet to health clinics will be given to the caregiver to show at the clinic (Appendix 5).

Two thirds of the participants will receive azithromycin MDA, which is expected to reduce mortality and offer other health benefits to the participants. At the end of the trial, if the analysis suggests a mortality reduction by azithromycin, all participants who are 1-11 months (29-364 days) old, will receive 20 mg / kg azithromycin.

6.04. Amount of collected blood and other biospecimens

From most participants, there will be no biological specimen collection.

At four time points, for a sub-sample of approximately 1,350 4-14 month old children and the same number of 49-59 month old children, we will also collect rectal and nasopharyngeal or rectal swabs. At each time point, the children will be different, so for each child, there will only be one sampling. Additionally, at visit 11 we will sample 15-26-months old children who have received 1-4 doses of MDA 1 year earlier in villages that have stopped MDA. In the Koulikoro or Kati (tertiary AMR sample) region we will sample 24-35 months old children who have received 1-4 doses of MDA 1 year earlier and who live in a village that has continued MDA. This will allow us to assess whether azithromycin resistant bacteria survive in individuals that have previously been treated. We will use flocculated swabs for the nasopharyngeal and stool samples. The estimated mass of the collected stool is 200 ug.

From a subgroup of 6–8-month-old or 12-14 month old infants and young children, there will be one venous blood collection, with a total collected blood volume of maximum of 5 ml. The collected volume represents approximately 1% of the infant's total blood volume and it is replaceable from the infants' own blood production within 24 hours. From the same individuals, we will collect 2-10 g of stool and 5-10 ml of urine

6.05. Insurance coverage

The Malian trial team will purchase an insurance that will cover professional liabilities for the study team and all medical costs for study participants, caused by their participation in LAKANA trial.

Any adverse reactions arising during the study will be reported to the study team through the appropriated process and then referred into the national health system. Medical costs will be covered by the study budget.

The team will be provided with security services when deemed necessary by national security advice. Tampere University will purchase additional security services for all expatriate staff, consisting of travel advice, security briefings and assistance in medical and other emergencies. All visiting study team members and guests will be required to abide by security advice and procedures.

6.06. Compensation to study participants

For most LAKANA participants, the scheduled home visits (9 in total) will take approximately 20 –30 minutes. For time used on these visits, the households will not be compensated.

For children participating in sub-samples, participants will be invited to a nearby health facility or central location in a village and visits will include collection of detailed data on household economic, biological specimen collection or measuring anthropometrics. Such visits are expected to take approximately an hour. For these visits, the participants will be compensated with approx. $2-$5 or another amount deemed appropriate by the local study coordination team to cover transport. If blood is drawn, some food and drink will be offered.

For children participating in sub-samples and screened for Malaria at visit 9, the study nurses will provide the result of the rapid diagnostic test to the caregivers. The nurses will actively refer the children with a positive test result to the nearest health clinic where they can receive the appropriate care according to the Mali national treatment guidelines in use at that time. The health clinics will be compensated by the study team on a regular basis for the treatment costs incurred.

6.07. Payments to the trial organisers

Trial organisers do not receive any incentives for enrolling participants to the study. During the trial period, they will receive their normal salary from their employers or a personal stipend supporting postgraduate studies and living expenses.

6.08. Data safety and monitoring board (DSMB)

A review board will be constituted prior to commencement of the study to oversee the progress and assess the safety of the intervention. The trial team will provide the DSMB members a monthly summary of trial progress and documented and expected numbers of serious adverse events (SAEs).

The DSMB will meet in person before the trial onset and conduct thereafter scheduled electronic meetings 1-2 times a year, to discuss the progress and safety data. Additionally, any member of the DSMB may request an additional meeting at any point.

As part of the adaptive design, there will a planned interim data analysis, as explained in chapter 4.13. In case of any unusual events, members of the DSMB may request additional interim analyses at their discretion.

6.09. Stopping rules for the study

The DSMB will convene before the onset of the trial to agree on specific stopping rules for the trial.
Tentatively, DSMB may recommend termination, dropping the placebo arm, or other modification of the study if:

1. In an interim analysis, there is strong evidence of a mortality benefit (reduced mortality) from the study intervention. The DSMB will agree on the exact definition of "strong evidence", but according to the Peto-rule, in an interim analysis a mortality difference should be considered statistically significant only if the 2-sided p-value is lower than approximately 0.001 (Peto et al., 1976). Simulation studies have shown that the Peto's rule is not overly extreme and results in practically no inflation of type 1 error in the final analysis (Freidlin et al., 1999).
2. In an interim analysis, there is strong suspicion of harm (increased mortality or incidence of other SAEs) from the study intervention. For harm assessment, no fixed statistical rules will be applied, but the DSMB will holistically consider point estimates and confidence intervals for mortality / SAE incidence differences, p-values from appropriate statistical tests and other relevant factors, when determining its recommendation about study continuation or discontinuation.
3. In an interim analysis, there is strong evidence of futility (mathematical expectation predicting from the accrued data that the major study hypotheses cannot be supported with statistical significance with the originally planned sample size).

While stopping rules will be utilized to help the DSMB to assess the justification of trial continuation, the DSMB will consider the balance of risks and benefits as well as consistency with external evidence. Thus, the DSMB recommendation to continue or

discontinue will not be based on any single issue or value in any of the analyses but a comprehensive analysis considering multiple issues around the trial.

7. Personnel, resources, and study management

7.01. Research institutions

The main participating research institutions are CVD-Mali in Mali (CVD-Mali), Tampere University in Finland (TAU), University College London in UK (UCL) and Tro Da Ltd, a UK-based global health consulting company.

The project will be implemented by CVD-Mali (Mali), Tampere University (Finland), University College London (UK), and Tro Da Ltd (UK). The partners will all contribute to trial design, implementation, analysis, and reporting. For their specific contributions, TAU will act as the coordinating institution and main grantee and provide expertise in clinical trials, social science, and certain laboratory analyses, CVD-Mali will bear the main responsibility of the trial implementation and provide knowledge on research and health in Mali, UCL will coordinate the AMR and economics work and provide expertise on these subjects and azithromycin mechanisms, and Tro Da Ltd will provide expertise in MDA platforms and support the team in trial implementation and internal communication.

All the participating organizations have extensive experience from conducting large clinical trials in Africa, coordinating international networks of researchers, and managing large multi-site grants. The principal investigators (PIs) and the Co-PIs have also earlier experience from working together: the Mali and TAU PIs on another clinical trial involving azithromycin treatment in Mali and the UCL and TAU ones from multiple projects in Malawi and elsewhere in East Africa.

All the organizations are equipped with necessary research infra-structure in their main offices. For trial implementation, CVD-Mali will need to review health facilities in the Kayes region and likely refurbish some office and basic laboratory facilities (including e.g. freezer purchase). TAU and UCL will need to identify 1-2 new scientists, but otherwise their project personnel have already been identified. At CVD-Mali, there is also a need to identify 1-2 more scientists to work on the project. In addition, there is a need to hire the entire team of data collectors and field supervisors at CVD-Mali.

7.02. Principal investigators

Dr. Per Ashorn (overall PI) has coordinated the design of the LAKANA trial and will bear the overall responsibility of its completion. He will also act as a supervisor to Finnish or Malian students involved in the project and enrolling for post-graduate studies

at Tampere University. Dr. Per Ashorn serves as a Professor of Paediatrics and Director of the Center for Child Health Research at Tampere University. He is a medical specialist in paediatrics and paediatric infectious diseases, and he has carried out research on child health and nutrition in low-income settings for more than 25 years. Dr. Per Ashorn has formal training in and extensive experience from designing, coordinating and reporting clinical trials, including two on the use of azithromycin to promote child health.

Dr. Samba O. Sow (CVD-Mali co-PI) will be responsible for the practical implementation of the trial in Mali. He serves as Director-General of the Center for Vaccine Development (CVD) in Mali and Professor of Medicine at the School of Medicine, University of Maryland (USA), where he has coordinated clinical trials and epidemiological studies on a range of vaccine-preventable diseases. A medical doctor and epidemiologist, he has received numerous honors, including the French Legion of Honor and National Order of Merit and Officer in 2017 for his work on the Ebola epidemic. He was the Commemorative Fund Lecturer of the American Society of Tropical Medicine and Hygiene in 2006 and held the post of Minister of Health for Mali from April 2017 until May 2019.

Dr. Nigel Klein (UCL co-PI) will lead the MDA antimicrobial resistance component in the LAKANA trial. He works as a Professor and Consultant in Pediatric Infectious Diseases and Immunology at Great Ormond Street Children's Hospital (GOH), London, and Professor of Infection and Immunity at the UCL Great Ormond Street Institute of Child Health (ICH). He has established and led the Infectious Diseases and Microbiology Unit at ICH the Department of Infection at UCL and was recently appointed as Lead of the Paediatric Programme at the Wellcome Trust funded African Health Research Institute in KwaZulu-Natal. Dr. Klein has been working in the field of Infectious Diseases for more than 30 years, published more than 400 research papers and supervised over 60 PhD students. He has a particular interest in pathogenesis of infection and conducts also microbiome research, including two studies with azithromycin and other antibiotic interventions during pregnancy in low-income settings. He is key to a newly established Precision AMR Centre at UCL, GOH and ICH that provides infrastructure for both molecular and phenotypic technologies to examine antimicrobial resistance and will support capacity building in Mali where needed.

Dr. Ulla Ashorn (TAU co-PI) will coordinate the sub-study on MDA equity, feasibility and acceptability, contribute to all other parts of the trial and bear the responsibility of international program management. She will also act as a supervisor to Finnish or Malian students involved in the project and enrolling for post-graduate studies at Tampere University. Dr. Ulla Ashorn is a social scientist with background in public and global health. She currently works as a Senior Scientist at the Center for Child Health Research at Tampere University. She has conducted research on women's and children's health mainly in East Africa. She is familiar with both qualitative and quantitative health research methods as well as policy and politics research including stakeholder analysis and engagement.

Dr Camilla Ducker (Tro Da co-PI) will be working with CVD-Mali on implementation, communication, and helping to build the capacity of personnel recruited and assisting on the equity, feasibility and acceptability sub-study. Dr. Ducker is a clinical doctor and public health consultant. She launched her own global health company Tro Da Ltd in

2018, which has successfully carried out work for the Bill and Melinda Gates Foundation (REACH program), the Task Force for Global Health (laboratory capacity strengthening), and the World Health Organization. Prior to that, Camilla worked for the UK Government's Department of International Development, managing the majority of its Neglected Tropical Disease programs. Camilla Chairs the Neglected Tropical Disease Equity Working Group.

Dr. Yin Bun Cheung will act as the principal statistician on the project and advise other biostatisticians at Tampere University on analytic strategy, statistical programming and interpretation of the trial findings and provide troubleshooting where necessary. Dr. Cheung is a biostatistician with background in paediatric epidemiology and infectious disease clinical trials. He serves as a Professor at the Centre for Quantitative Medicine at Duke-NUS Medical School, Singapore. He is also an Adjunct Professor of International Health at the Tampere University, Finland. He has been the principal investigator of multiple research projects funded by the National Medical Research Council, Singapore, to improve statistical methods in infectious disease research. He has studied child health in Asian and African countries and is the author of Statistical Analysis of Human Growth and Development (2014) and co-author of Survival Analysis: A Practical Approach (2006).

7.03. Study statisticians

Dr. Yin Bun Cheung will act as the principal statistician on the project. Data review and cleaning, as well as data and metadata annotation and public posting will be completed by two biostatisticians at Tampere University, Ms. Lotta Hallamaa and Mr. Juho Luoma. They both have an MSc degree in biostatistics and experience from data cleaning and analysis. Ms. Hallamaa is currently completing her PhD on long-term follow-up of Malawian children, whose mothers were treated with azithromycin in pregnancy.

7.04. Other main scientists and their responsibilities

Dr Elaine Cloutman-Green will ensure high standard laboratory practice and diagnostic stewardship for the AMR studies. She is the National Institute of Health Research national diagnostic infection lead in the UK. She sits on the UK AMR diagnostic stewardship committee and is a Principal Clinical Scientist at Great Ormond Street Hospital (GOSH), leads the Precision AMR facility at GOSH and holds a clinical lectureship at UCL.

7.05. Other employed staff

There will be a study team based in Bamako for overall supervision of the trial. This team will be made up of a trial project manager, data managers, administrators, and financial managers.

In additional to the central Bamako office, there will be a regional office in Kita City and 6-9 district offices. These will employ a supervisor who will supervise a group of data collectors.

At each of the study districts, there will be several field teams that will implement the azithromycin MDA and collect study data. Each team will be made up of one GCP-trained data collector and at least two *relais* workers (community volunteers) who will act as community drug distributors in conjunction with a data collector. In total, the team will hire one hundred and eighty data collectors for the main study and each of them will cover 3-6 villages (together with the community volunteers).

For the analyses on morbidity, growth, AMR, azithromycin mechanisms, feasibility, cost and safety, an additional research team will be posted in the four health facilities where data collection on these additional variables takes place. The team will consist of a nurse, two anthropometrists and one motorcycle messenger. The nurse will collect biospecimens, monitor health facility visits by trial participants, and interview families about possible side-effects. The anthropometrists will measure child size, interview families about feasibility and economics, and help the nurse in other issues. The motorcycle messenger will help gather the participants and deliver biospecimens daily to the study laboratories in Kita.

Biological samples collected within LAKANA are planned to be primarily shipped to a site-laboratory that the LAKANA team will establish in the city of Kita. In this laboratory, two laboratory assistants will do primary processing and storage of the samples. For further analyses, the samples will be shipped to CVD-Mali laboratory in Bamako. For high-quality analyses, all laboratories will be provided with high quality laboratory equipment. Laboratory personnel specific to the project will be recruited locally and capacity built by the national and international institutions involved in the trial.

Most of the employed study personnel will be Malians as many as possible data collectors and supervisors will be hired locally. This will ensure the trust of the population who are receiving the intervention and contribute to local capacity building.

7.06. Arrangements for day-to-day management of the study

The overall, general management of the study will be coordinated by the lead PI and Tampere University. Operational and day-to-day management will be the responsibility of CVD-Mali in collaboration with Tro Da.

The study team at CVD-Mali will have a management group, led by the Malian co-PI and responsible for trial implementation. The management group will meet weekly, to review progress reports on enrollment and follow-up, to discuss any problems encountered and to accept a general plan of activities for the subsequent week. A data manager will produce weekly progress reports of the success of enrollment and follow-up as well as data quality, for use by the core group.

A CVD-Mali project manager will produce weekly duty lists for data collectors and supervisors, using custom-made computer software that is linked to the trial database. S/he and her sub-ordinates will distribute these plans to members of the study team at the end of each week. Once a month, the study team will have a larger staff meeting to discuss the progress and any acute issues related to the study.

7.07. Communication plan

For external communication, there will be a trial website in national and international languages to cascade information. The study team will also prepare an electronic quarterly newsletter, to be shared with local partners, Ministry of Health and the wider scientific community. Tampere University will prepare regular progress reports to the funder, as agreed in the grant contracts.

In addition to the website and quarterly newsletters, the team will organize a national stakeholder meeting once a year and regional stakeholder meetings as needed. Before onset of data collection, there will be multiple launches of the trial at district level. During the study, there will be district level and village level community meetings to distribute information. There will also be a system to allow village elders and CSCOM staff to raise issues through the CDDs and Data collectors, so that local additional level meetings can be organised as needed, to deal with rumours or any other community anxieties.

The internal trial communication will be coordinated and conducted by the study team in Mali. Data Collectors will meet with their supervisors on a weekly basis and feedback any issues and the central team in Bamako will receive weekly reports from each office and feedback also. The report will include highlights, activities, risks and future activities planned.

Leaflets in local languages and using infographics will be developed and tested in the community to communicate the trial to the communities.

7.08. Project Steering Group

LAKANA trial implementation will be overseen by a 5-member project steering group (PSG) that will consist of the overall PI and the four co-PIs from the organizations that will receive the project funding (Ashorn, Sow, Klein, Ashorn, Ducker). The PSG will meet electronically at 1-2-week intervals at the beginning of data collection and later at 1-2-month intervals, to review progress and plan for future. A trial statistician from TAU will produce monthly progress reports of the success of enrollment and follow-up as well as data quality, for use by the PSG.

The PSG will make all the key strategic decisions over the trial, i.e. it will decide submission of the project plan and reports the funder, to IRBs, and to other key authorities. The PSG will also make decisions on amendments to trial protocol, project

personnel or budget, or data ownership and use.

7.09. National and international advisory groups

There will be two advisory groups to the study team: A National Advisory Committee (NAC) and an international Technical Advisory Group (TAG).

The NAC will consist of approximately 10 health professionals, who will advise the Research team on general design issues, implementation strategy, conduct and progress of the trial. It will also be consulted to provide advice on any ad-hoc national issues that arise during the trial. The NAC will provide insights to the team especially regarding stakeholder interests and priorities as well as other relevant initiatives taking place at the study site. The NAC will also provide technical advice, assist with resolving issues and risks especially at a community level, use influence and authority to assist the project in achieving its outcomes, and communicate about the project in their respective organizations.

Approximately once a year, the NAC will organize a wider stakeholder meeting. This meeting, attended by 100 – 150 participants especially from the study area, will serve mostly an information purpose, but also, to help the study team with details of trial planning and implementation and related communication in the communities.

The TAG will consist of three members, who are internationally recognized experts in the fields of child survival, clinical trials, mass drug administration, or antimicrobial resistance. It will advise the PIs and co-PIs on general design issues, implementation strategy, conduct and progress of the trial. It will also be consulted to provide advice on any ad-hoc technical issues that arise during the trial. It will discuss DSMB reports and recommendations and advise the trial team on their implementation.

8. Training provided

The study will offer numerous training opportunities to Malian and other undergraduate, graduate and post-graduate students, but no specific projects have yet been identified. The budget includes a 4-year remuneration for one Malian PhD student who would tentatively study the feasibility and acceptability questions.

9. Possible constraints

Heavy rains between June and September may reduce human mobility and access to some locations and hence complicate the MDA and data collection. The team will, however, make extra preparations with rain gear etc and hence the delays or missed visits

are expected to be non-significant.

Safety (for the population and the research team) is another possible constraint. The Northern part of Mali is politically instable and there is a safety concern in that area. Most of Kayes Region, where the study is being planned is, however, stable and there is no reason to expect a change in that situation. CVD-Mali has budgeted for security support for internal travel.

10. Funding

Tampere University has received funding for the proposed trial from the Bill & Melinda Gates Foundation (Seattle, WA, USA). For implementation, Tampere University will issue subawards to CVD-Mali, UCL, and Tro Da Ltd.

11. References

American Academy of Pediatrics: Report of the committee on Infectious disease. Red Book 2012.

Arzika AM, Maliki R, Boubacar N, Kane S, Cotter SY, Lebas E, Cook C, Bailey RL, West SK, Rosenthal PJ, Porco TC, Lietman TM, Keenan JD; MORDOR Study Group. Biannual mass azithromycin distributions and malaria parasitemia in pre-school children in Niger: A cluster-randomized, placebo-controlled trial. PLoS Med. 2019 Jun 25;16(6):e1002835. doi: 10.1371/journal.pmed.1002835. eCollection 2019 Jun.

Asiimwe C, Kyabayinze DJ, Kyalisiima Z, Nabakooza J, Bajabaite M, Counihan H, Tibenderana JK: Early experiences on the feasibility, acceptability, and use of malaria rapid diagnostic tests at peripheral health centres in Uganda-insights into some barriers and facilitators. Implementation Science 2012; 7:5

Bauer MS, Damschroder L, Hagedorn H, Smith J, Kilbourne AM. An introduction to implementation science for the non-specialist. BMC Psychol. 2015;3(1):32.

Beigelman A, Isaacson-Schmid M, Sajol G, Baty J, Rodriguez OM, Leege E, et al. Randomized trial to evaluate azithromycin's effects on serum and upper airway IL-8 levels and recurrent wheezing in infants with respiratory syncytial virus bronchiolitis. The Journal of allergy and clinical immunology. 2015 May;135(5):1171-8 e1.

Blake IM, Burton MJ, Solomon AW, West SK, Basáñez MG, Gambhir M, Bailey RL, Mabey DC and Grassly NC. Targeting antibiotics to households for trachoma control. PLoS Negl Trop Dis, 2010; 4(11): e862.

Bourke CD, Gough EK, Pimundu G, Shonhai A, Berejena C, Terry L, Baumard L, Choudhry N, Karmali Y, Bwakura-Dangarembizi M, Musiime V, Lutaakome J, Kekitiinwa A, Mutasa K, Szubert AJ, Spyer MJ, Deayton JR, Glass M, Geum HM, Pardieu C, Gibb DM, Klein N, Edens TJ, Walker AS, Manges AR, Prendergast AJ. Cotrimoxazole reduces systemic inflammation in HIV infection by altering the gut microbiome and immune activation. Sci Transl Med. 2019 Apr 3;11(486).

Centers for Disease control and Prevention. Pertusis: Treatment. Available https://www.cdc.gov/pertussis/clinical/treatment.html. Accessed 02.06.2019.

Chandramohan D, Dicko A, Zongo I, Sagara I, Cairns M, Kuepfer I, Diarra M, Barry A, Tapily A, Nikiema F, Yerbanga S, Coumare S, Thera I, Traore A, Milligan P, Tinto H, Doumbo O, Ouedraogo JB, Greenwood B. Effect of Adding Azithromycin to Seasonal Malaria Chemoprevention. N Engl J Med 2019; 380:2197-2206

Church JA, Rukobo S, Govha M, Lee B, Carmolli MP, Chasekwa B, Ntozini R, Mutasa K, McNeal MM, Majo FD, Tavengwa NV, Moulton LH, Humphrey JH, Kirkpatrick BD, Prendergast AJ. The impact of improved water, sanitation and hygiene on oral rotavirus vaccine immunogenicity in Zimbabwean infants: sub-study of a cluster-randomized trial. Clin Infect Dis. 2019 Feb 16. pii: ciz140. doi: 10.1093/cid/ciz140. [Epub ahead of print]

Coles CL, Seidman JC, Levens J, Mkocha H, Munoz B, West S. Association of mass treatment with azithromycin in trachoma-endemic communities with short-term reduced risk of diarrhea in young chil-dren. Am J Trop Med Hyg 2011; 85: 691-6.

Coles CL, Levens J, Seidman JC, Mkocha H, Munoz B, West S. Mass distribution of azithromycin for trachoma control is associated with short-term reduction in risk of acute lower respiratory infection in young children. Pediatr Infect Dis J 2012; 31: 341-6.

Conteh L, Patouillard E, Kweku M, Legood R, Greenwood B, Chandramohan D. Cost effectiveness of seasonal intermittent preventive treatment using amodiaquine & artesunate or sulphadoxine-pyrimethamine in Ghanaian children. PLoS One. 2010 Aug 17;5(8):e12223. doi: 10.1371/journal.pone.0012223. PubMed PMID: 20808923; PubMed Central PMCID: PMC2923188.

Dewey KG, Adu-Afarwuah S. Systematic review of the efficacy and effectiveness of complementary feeding interventions in developing countries. Matern Child Nutr. 2008 Apr;4 Suppl 1:24-85. doi: 10.1111/j.1740-8709.2007.00124.x.

Eberly MD, Eide MB, Thompson JL, Nylund CM. Azithromycin in early infancy and pyloric stenosis. Pediatrics. 2015 Mar;135(3):483-8.

Emerson PM, Hooper PJ, Sarah V. Progress and projections in the program to eliminate trachoma. PLoS Negl Trop Dis 2017; 11(4): e0005402.

Ferwerda A1, Moll HA, Hop WC, Kouwenberg JM, Tjon Pian Gi CV, Robben SG, de Groot R. Efficacy, safety and tolerability of 3 day azithromycin versus 10 day co-amoxiclav in the treatment of children with acute lower respiratory tract infections. J Antimicrob Chemother 2001;47:441-6.

Fitzgerald FC, Lhomme E, Harris K, Kenny J, Doyle R, Kityo C, Shaw LP, Abongomera G, Musiime V, Cook A, Brown JR, Brooks A, Owen-Powell E, Gibb DM, Prendergast AJ, Walker AS, Thiebaut R, Klein N; CHAPAS-3 Trial Team. Microbial translocation does not drive immune activation in Ugandan children with HIV. J Infect Dis. 2019 Jan 1; 219(1): 89–100

Freidlin B, Korn EL, LGeorge SL. Data Monitoring Committees and Interim Monitoring Guidelines. Control Clin Trials 1999;20:395–407

Frick KD, Lietman TM, Holm SO, Jha HC, Chaudhary JS, Bhatta RC. Cost-effectiveness of trachoma control measures: comparing targeted household treatment and mass treatment of children. Bulletin of the World Health Organization. 2001;79:201-7.

Fry AM, Jha HC, Lietman TM, et al. Adverse and beneficial secondary effects of mass treatment with azithromycin to eliminate blindness due to trachoma in Nepal. Clin Infect Dis 2002; 35: 395-402.

Gaynor BD, Amza A, Kadri B, et al. Impact of mass azithromycin distribution on malaria parasitemia during the low-transmission season in Niger: a cluster-randomized trial. Am J Trop Med Hyg 2014; 90: 846-51.

Gedge LM, Bettis AA, Bradley MH, Hollingsworth TD, Turner HC. Economic evaluations of lymphatic filariasis interventions: a systematic review and research needs. Parasites & vectors. 2018 Dec;11(1):75.

Gibbons D, Fleming P, Virasami A, Michel ML, Sebire NJ, Costeloe K, et al. Interleukin-8 (CXCL8) production is a signatory T cell effector function of human newborn infants. Nat Med. 2014;20(10):1206-10

Gkazi AS, Margetts BK, Attenborough T, Mhaldien L, Standing JF, Oakes T, et al. Clinical T Cell Receptor Repertoire Deep Sequencing and Analysis: An Application to Monitor Immune Reconstitution Following Cord Blood Transplantation. Front Immunol. 2018;9:2547.

Gough EK, Moodie EE, Prendergast AJ, Johnson SM, Humphrey JH, Stoltzfus RJ, Walker AS, Trehan I, Gibb DM, Goto R, Tahan S, de Morais MB, Manges AR. The impact of antibiotics on growth in children in low and middle income countries: systematic review and meta-analysis of randomised controlled trials. BMJ. 2014 Apr 15;348:g2267. doi: 10.1136/bmj.g2267. Review.

Keenan JD, Ayele B, Gebre T, et al. Childhood mortality in a cohort treated with mass azithromycin for trachoma. Clin Infect Dis 2011; 52: 883-8.

Keenan JD, Bailey RL, West SK, Arzika AM, Hart J, Weaver J, Kalua K, Mrango Z, Ray KJ, Cook C, Lebas E, O'Brien KS, Emerson PM, Porco TC, Lietman TM; MORDOR Study Group. Azithromycin to Reduce Childhood Mortality in Sub-Saharan Africa. N Engl J Med. 2018 Apr 26;378(17):1583-1592. doi: 10.1056/NEJMoa1715474

Kolaczinski JH, Robinson E, Finn TP. The cost of antibiotic mass drug administration for trachoma control in a remote area of South Sudan. PLoS Negl Trop Dis 2011; 5(10): e1362

Kwiatkowska B, Maślińska M. Macrolide therapy in chronic inflammatory diseases. Mediators Inflamm. 2012;2012:636157. doi: 10.1155/2012/636157. Epub 2012 Aug 21. Review.

Langly LM, Halperin SA, Boucher FD, Smith B, and the Pediatric Investigators Collaborative Network on Infections in Canada (PICNIC). Azithromycin Is as Effective as and Better Tolerated Than Erythromycin Estolate for the Treatment of Pertussis. Pediatrics. 2004;114:e96

McCallum GB, Morris PS, Chatfield MD, Maclennan C, White AV, Sloots TP, et al. A single dose of azithromycin does not improve clinical outcomes of children hospitalised with bronchiolitis: a randomised, placebo-controlled trial. PloS One. 2013;8(9):e74316

Mack I, Sharland M, Berkley JA, Klein N, Malhotra-Kumar S, Bielicki J. Antimicrobial Resistance Following Azithromycin Mass Drug Administration: Potential Surveillance Strategies to Assess Public Health Impact. Clin Infect Dis. 2019 Oct 21. pii: ciz893. doi: 10.1093/cid/ciz893. [Epub ahead of print]

NICE International. Health Intervention and Technology Assessment Program, Thailand, Centre for Health Economics, University of York, Bill and Melinda Gates Foundation. The Gates Reference Case, What It Is, Why It Is Important, and How to Use It National Institute for Health and Care Excellence, London 2014.

NICE international, The Gates Reference Case What it is, why it's important, and how to use it. 2014

Nonvignon J, Aryeetey GC, Issah S, Ansah P, Malm KL, Ofosu W, Tagoe T, Agyemang SA, Aikins M. Cost-effectiveness of seasonal malaria chemoprevention in upper west region of Ghana. Malar J. 2016 Jul 16;15:367. doi: 10.1186/s12936-016-1418-z. PubMed PMID: 27423900; PubMed Central PMCID: PMC4947302.

Parker EP, Ramani S, Lopman BA, Church JA, Iturriza-Gómara M, Prendergast AJ, Grassly NC. Causes of impaired oral vaccine efficacy in developing countries. Future Microbiol. 2018 Jan;13:97-118

Peto R, Pike MC, Armitage P, Breslow NE, Cox DR, Howard SV, Mantel N, McPherson K, Peto J, Smith PG. Design and analysis of randomized clinical trials requiring prolonged observation of each patient. I. Introduction and design. Br J Cancer. 1976;34:585-612.

Pinto LA, Pitrez PM, Luisi F, de Mello PP, Gerhardt M, Ferlini R, et al. Azithromycin therapy in hospitalized infants with acute bronchiolitis is not associated with better clinical outcomes: a randomized, double-blinded, and placebo-controlled clinical trial. J Pediatr 2012 Dec;161(6):1104-8.

Pitt C, Ndiaye M, Conteh L, Sy O, Hadj Ba E, Cissé B, Gomis JF, Gaye O, Ndiaye JL, Milligan PJ. Large-scale delivery of seasonal malaria chemoprevention to children under 10 in Senegal: an economic analysis. Health Policy Plan. 2017 Nov 1;32(9):1256-1266. doi: 10.1093/heapol/czx084. PubMed PMID: 28981665; PubMed Central PMCID: PMC5886061.

Porco TC, Gebre T, Ayele B, et al. Effect of mass distribution of azithromycin for trachoma control on overall mortality in Ethiopian children: a randomized trial. JAMA 2009; 302: 962-8.

Porco TC, Hart J, Arzika AM, Weaver J, Kalua K, Mrango Z, Cotter SY, Stoller NE, O'Brien KS, Fry DM, Vanderschelden B, Oldenburg CE, West SK, Bailey RL, Keenan JD, Lietman TM; Macrolides Oraux pour Réduire les Décès avec un Oeil sur la Résistance (MORDOR) Study Group. Mass Oral Azithromycin for Childhood Mortality: Timing of Death After Distribution in the MORDOR Trial. Clin Infect Dis. 2019 May 30;68(12):2114-2116. doi: 10.1093/cid/ciy973.

Prendergast AJ, Szubert AJ, Berejena C, Pimundu G, Pala P, Shonhai A, Musiime V, Bwakura-Dangarembizi M, Poulsom H, Hunter P, Musoke P, Kihembo M, Munderi P, Gibb DM, Spyer M, Walker AS, Klein N; ARROW Trial Team. Baseline Inflammatory Biomarkers Identify Subgroups of HIV-Infected African Children With Differing

Responses to Antiretroviral Therapy. J Infect Dis. 2016 Jul 15;214(2):226-36.

Rogawski ET, Meshnick SR, Becker-Dreps S, Adair LS, Sandler RS, Sarkar R, Kattula D, Ward HD, Kang G, Westreich DJ. Reduction in diarrhoeal rates through interventions that prevent unnecessary antibiotic exposure early in life in an observational birth cohort. Journal of Epidemiology and Community Health. 2015 Nov 30. pii: jech-2015-206635. doi: 10.1136/jech-2015-206635

Roord JJ, Wold BHM, Goossens MMHT, Kimpen, JLL. Prospective Open Randomized Study Comparing Efficacies and Safeties of a 3-Day Course of Azithromycin and a 10-Day Course of Erythromycin in Children with Community-Acquired Acute Lower Respiratory Tract Infections. Antimicrobial Agents and Chemotherapy 1996;40:2765-2768.

Sánchez R1, Fernández-Baca V, Díaz MD, Muñoz P, Rodríguez-Créixems M, Bouza E. Evolution of susceptibilities of Campylobacter spp. to quinolones and macrolides. Antimicrobial Agents and Chemotherapy. 1994 Sep; 38(9):1879-82.

Sanders GD, Neumann PJ, Basu A, Brock DW, Feeny D, Krahn M, Kuntz KM, Meltzer DO, Owens DK, Prosser LA, Salomon JA. Recommendations for conduct, methodological practices, and reporting of cost-effectiveness analyses: second panel on cost-effectiveness in health and medicine. Jama. 2016 Sep 13;316(10):1093-103.

Sandgaard KS, Lewis J, Adams S, Klein N, Callard R. Antiretroviral therapy increases thymic output in children with HIV. AIDS. 2014;28(2):209-14

Schachterle SE, Mtove G, Levens JP, et al. Short-term malaria reduction by single-dose azithromycin during mass drug administration for trachoma, Tanzania. Emerg Infect Dis 2014; 20: 941-9.

Schémann JF, Guinot C, Traore L, Zefack G, Dembele M, Diallo I, Traore A, Vinard P, Malvy D. Longitudinal evaluation of three azithromycin distribution strategies for treatment of trachoma in a sub-Saharan African country, Mali. Acta tropica. 2007 Jan 1;101(1):40-53.

Sekhon M, Cartwright M, Francis JJ: Acceptability of healthcare interventions: an overview of reviews and development of a theoretical framework. BMC Health Services Research 2017; 17: 88

Shaw LP, Bassam H, Barnes CP, Walker AS, Klein N, Balloux F. Modelling microbiome recovery after antibiotics using a stability landscape framework. ISME J. 2019 Jul;13(7):1845-1856.

Smith C, Oluwaseun E, Choonara I, Kotecha S, Jacqz-Aigrain E, Sammons H. Use and safety of azithromycin in neonates: a systematic review. BMJ Open 2015;5:e008194. doi: 10.1136/bmjopen-2015-008194

Sohn, H., Tucker, A., Ferguson, O. et al. Costing the implementation of public health interventions in resource-limited settings: a conceptual framework. Implementation Sci

15, 86 (2020)

Turner HC, Truscott JE, Hollingsworth TD, Bettis AA, Brooker SJ, Anderson RM. Cost and cost-effectiveness of soil-transmitted helminth treatment programmes: systematic review and research needs. Parasites & vectors. 2015 Dec;8(1):355.

Turner HC, Walker M, Pion SD, McFarland DA, Bundy DA, Basáñez MG. Economic evaluations of onchocerciasis interventions: a systematic review and research needs. Tropical Medicine & International Health. 2019 Apr 23.

Uchiyama R, Chassaing B, Zhang B, Gewirtz AT. The Antibiotic treatment suppresses rotavirus infection and enhances specific humoral immunity. J Infect Dis 2014;210:171–82

UNICEF, State of World's Children, 2017

United Nations Development Program, Sustainable development goals: Goal 3 targets. http://www.undp.org/content/undp/en/home/sustainable-development-goals/goal-3-good-health-and-well-being/targets/. Accessed 02.06.2019

Whitty CJ, Glasgow KW, Sadiq ST, Mabey DC, Bailey R. Impact of community-based mass treatment for trachoma with oral azithromycin on general morbidity in Gambian children. Pediatr Infect Dis J 1999; 18: 955-8

Wilkinson T, Sculpher MJ, Claxton K, Revill P, Briggs A, Cairns JA, Teerawattananon Y, Asfaw E, Lopert R, Culyer AJ, Walker DG. The international decision support initiative reference case for economic evaluation: an aid to thought. Value in health. 2016 Dec 1;19(8):921-8.

World Health Organization 2004. Making choices in health: WHO guide to cost-effectiveness analysis [Online]. Geneva: World Health Organization. Available at: <http://www.who.int/choice/publications/p_2003_generalised_cea.pdf>

WHO. Child growth standards, technical report. The World Health Organization, 2006

World Health Organisation. The world health report: health systems financing: the path to universal coverage. Geneva: World Health Organisation, 2010.

World Health Organisation. Making fair choices on the path to universal health coverage. Geneva: World Health Organisation, 2014.

WHO, Causes of child mortality 2018. http://www.who.int/gho/child_health/mortality/causes/en/. Accessed 02.06.2019

Wijeyesekera A, Wagner J, De Goffau M, Thurston S, Rodrigues Sabino A, Zaher, S, White D, Ridout J, Peters MJ, Ramnarayan P, Branco RG, Torok ME, Valla F,Meyer R, Klein N, Frost G, Parkhill J, Holmes E, Pathan N. Multi-CompartmentProfiling of Bacterial and Host Metabolites Identifies Intestinal Dysbiosis and Its Functional Consequences in the Critically Ill Child. Crit Care Med. 2019 Jun DOI:

10.1097/CCM.0000000000003841

World Bank. Open Data. 2019 [cited 25 July 2019]. Available from: https://data.worldbank.org/

Xu Y, Lee CF, Cheung YB. Analyzing binary outcome data with small clusters: A simulation study. Communications in Statistics - Simulation and Computation,43:7, 1771-1782, DOI: 10.1080/03610918.2012.744044

12. Appendices

Appendix 1a: Information about the trial to village leadership

(English version, to be translated into French and Bambara and read aloud to village leaders

CENTRE POUR LE DEVELOPPEMENT DES VACCINS, MALI

Director General: Samba Ousmane SOW, M.D., M.Sc., FASTMH

Professor of Medicine

Infectious Diseases Project: Measles Vaccine Initiative ♦ Enteric Disease Project ♦ Pneumococcus, Meningococcus, & *Haemophilus influenzae* Prevention Program
Malaria Project: Bandiagara Malaria Project ♦ Malaria Training Program

CVD-MALI

Large-scale Assessment of the Key health-promoting Activities of New mass drug administration regimens with Azithromycin (LAKANA)

Information for Participants and Informed Consent Forms for village leaders

This document contains information for villages participating in a cluster-randomised, double-blinded, parallel group, controlled trial, to test the effects of mass drug administration of azithromycin on mortality and other outcomes among 1-11-month-old infants in rural Mali. It also contains the necessary consent forms for participants to enrol in the trial.

Key Investigators

Centre pour le Développement des Vaccins–Bamako, Mali

Per Ashorn, MD, PhD [1], Ulla Ashorn, PhD [1], Yin Bun Cheung, PhD [1,2], Camilla Ducker, MSc., MBBS [3], Nigel Klein, MBBS, PhD [4], Samba O. Sow MD., MSc., FASTMH [5].

Participating Academic Institutions

[1] Tampere University, Finland; [2] Duke-NUS Medical School, Singapore; [3] Tro Da Ltd, UK; [4] University College London, UK; [5] CVD-Mali, Bamako, Mali.

Study Overview

Your village is invited to participate in the LAKANA trial, in which we will try to determine if a drug called azithromycin can help Malian infants to enjoy better health and better rates of survival. In the study, some infants will receive azithromycin and others will receive something called a placebo, which looks and tastes like azithromycin but does not contain any medicine. The study will compare these different groups of children.

Azithromycin is an effective and safe medicine that is commonly used to treat illnesses – it has been used for many years to successfully and safely treat infections, including millions of cases of an eye infection called trachoma. There are studies that show that periodically using this medicine to treat healthy children in West Africa might reduce mortality. The LAKANA trial team is investigating if such treatment would also be beneficial to Malian infants.

In total, we plan to include 50–60 000 children in the study, from approximately 1150 villages or urban areas in the Kayes, Kita and Koulikoro regions. If you give consent for your village to participate, the LAKANA team will visit the village every three months over the next two years, to ask you some questions, and give study medicine to all 1-11 month old infants living in the village.

Occasionally, children who take azithromycin may suffer stomach upsets for a short period, but otherwise we don't expect the treatment to cause any harmful side effects. Reactions, although rare, are always possible when medicines are given to children. So we will monitor the health of all the children who receive the medicine. In the unlikely case that a child in your village experiences a negative reaction, CVD-Mali will help that child to receive medical care and cover any costs related to that care.

This study could lead to major improvements in the health of infants in Mali. By participating in this trial, your village may be contributing to the development of better health programmes for the whole country. If this trial is successful, it is possible that azithromycin may be given to infants across Mali and West Africa.

You do not have to give permission for your village to take part in this study – you are free to decide if the village should participate or not.

What is the study about?

Azithromycin is a type of medicine, an antibiotic, that is commonly used to treat infections, including eye infections that cause blindness. Recent research studies have shown that infants in certain African countries had better survival rates when they took azithromycin every six months, when compared to those who did not take the medicine. In the LAKANA trial, researchers from the Centre pour le Développement des Vaccins-Mali (CVD-Mali) and their partners in Finland and the United Kingdom are studying how well azithromycin works in Mali. The trial is being funded by an American organization called the Bill & Melinda Gates Foundation.

The LAKANA trial will be carried out in 1150 villages and urban areas in the Kayes, Kita,

and Koulikoro regions, if local representatives have given permission for study activities to take place there. After receiving permission, in each village or town area, the study team will visit all households every three months for a period of two years. The team members will ask for permission to record and store a list of household members. If there are any 1-11 month old infants in the household, the household will be offered the chance to participate in the study and the infants living there will be offered the study medicine. At subsequent visits, team members will monitor the effects of the study medicine by asking questions about the infants' health.

Two types of study mixture will be given to infants in the LAKANA trial: azithromycin and placebo. Placebo is something that looks and tastes like azithromycin but does not contain any active ingredient that can treat infection. In one third of the study villages, all infants will receive azithromycin during all the study visits, every three-months. In another third of the study villages, infants will receive azithromycin at half of the visits and placebo at the other half. In the last third of the study villages, infants will always receive placebo.

Deciding which villages would be in which group was done by chance, like taking one single grain from a bag full of rice. During the study nobody will know whether the infants in your village are receiving placebo or active azithromycin at any of the home visits – this information will only be available later on to the study team. This way of carrying out the trial will help the study team to make correct conclusions about the health effects of azithromycin.

Study outcomes

The study will test to see if azithromycin has a positive impact on infant health and if it reduces infant mortality. The results of the study will be used to influence public health policy in Mali and in the wider sub-Saharan African region.

At approximately midway during the trial, researchers will analyze the obtained results. If there are clear indications that azithromycin is having a positive effect on mortality and the general health of infants, the trial will be modified, and azithromycin will then be given to all infants in the selected areas – and in other regions of Mali.

Who can take part in this study?

All households in the villages selected for the study will be offered the chance to join the study, for the purposes of collecting data. At each of the quarterly visits to households taking part in the study, all 1-11 month-old (age 29-364 days) infants will be offered the study medicine, as long as the infant has no known intolerance to azithromycin.

What will happen in the study?

If your village takes part in this study, the study team will visit the village four times a year for two years (eight times in total, after an initial visit to collect information). At each visit, we will ask participants questions about their households and their infants' health and illnesses, we will measure the weight of 1-11 month old infants, and will give them an appropriate dose (one teaspoonful or less) of study medicine. The medicine will

come in the form of a mixture and will be placed in the infant's mouth using a disposable syringe.

Since the study visits will take place at three-month intervals and only 1-11 month old (age 29-364 days) infants will be treated, the maximum number of treatments given to any individual infant will be four.

The first visit will take approximately 45 minutes, the subsequent visits will likely take approximately 15 minutes each.

If any household or child enrolled in the study is not at home when the study team comes to visit, they will try to visit again later or they may call to agree on a time when the missing people will be at home. If for some reason the visit cannot be completed, infants can still continue to take part in the study and will be seen during subsequent home visits.

How do I give consent for trial participation?

As representatives of your village, you will be asked for permission to carry out trial-related activities in the village. If we receive your permission, we will start the study activities. First of all, at each household, we will ask for permission to visit the household and ask some questions every three months for the next two years. Additionally, on each visit, we will ask for parents' or guardians' permission to weigh 1-11 month old infants and administer study medicine to them.

You don't need to sign any documents to give consent for the trial to take place in your village but we will record your consent on our computer. We will also ask an independent person to witness your consent if you are not literate. His or her name will also be recorded in the computer system.

Is participation voluntary?

Your village's participation in this study is entirely voluntary – for the village as a whole and for each individual household/child within the village. Your village does not have to take part in this study. Once enrolled, individual participants in the study will be free to leave the study at any time without giving a reason. Infants' participation will not change anything about the health care they receive at any health facilities. If, for any reason, infants are no longer available for follow-up visits, we will ask to contact them again to check on their health. Participants may refuse this as well. If a participant withdraws from this study, we will keep the information that we have already collected from infants in your village.

We will tell you about any significant new findings that are available during the study that may affect your willingness to involve infants in your village in the study.

Can villages / infants be removed from this study?

The study doctors and Malian, Finnish, or UK-based research committees overseeing the safety and rights of study participants, can remove your village and/or any infants in the village from the study or even stop the entire study without your approval if serious

health problems are noticed. If a parent or guardian decides to stop an infant's participation in the study, or if an infant's participation is ended, the study doctor or study staff may still ask the parents/guardian about that infant's participation in the study. Answering any such questions is, however, voluntary.

What are my village's responsibilities if we take part in this study?

If you agree to your village's participation in this research, we will visit households every three months and ask villagers to inform us if they move or expect to be unavailable. If villages and individuals agree to take part Taking part in this study does not change responsibilities with regard to other non-study related health care for infants in the village.

What risks are involved in azithromycin use and trial participation?

Azithromycin is commonly used and is known to be safe for infants. Even so, a few people may react to azithromycin and experience brief episodes of diarrhoea, nausea, abdominal pain and vomiting. A very small number of children may also experience liver problems, rashes, itching, or swelling of the lips or throat when they take azithromycin.

In extremely rare cases, some young infants may experience a problem where they have trouble passing food from the stomach to the intestines, which can cause vomiting and may require surgery. This can happen to any infant, whether they take azithromycin or not. If an infant experiences a reaction like this or has developed other symptoms, care should be sought urgently at the closest health facility and the study doctor should also be contacted.

If an infant in your village falls ill because of the study treatment, CVD-Mali will pay for the costs of medical treatment according to Malian standards of care.

What are the possible benefits of being in this study?

There may be no direct benefits to the people in your village as a result of participating in this study. However, the information obtained in this study will be important in deciding if azithromycin could improve the health of infants in Mali and other similar countries.

What kind of feedback can we expect from research results?

The results of the study will be known after it is finished. The study team will hold meetings to inform the community about the results.

What are the costs of taking part in this study?

All research activities will be performed free of charge.

What are the payments for being in this study?

There are no payments for taking part in the study. But infants in your village will be visited every three months while the study is active, and participants may signal other issues related to infants' health to the *relais* and data collector who will visit you.

What are the alternatives to participating in this study?

Giving azithromycin to infants in Mali is not currently part of routine care. The alternative to participating in the study is choosing not to take part.

How will information be kept private?

To protect the privacy of the parents/guardians and children living in your village, we will keep the information collected by the study secure. We will only allow authorized people to see it, including the study team from Mali, UK, and Finland, their representatives and the Ethics Committee in Mali, who act to protect people who take part in research. If study results are released to the public, the identities of participants will not be shared.

Who can answer questions about this study?

If you have any questions regarding your infant's participation or if an infant has a problem as a result of participating in this study, you may contact at any time Dr Fatoumata Diallo on (00223) 74 60 18 19, Dr Fadima Cheick Haidara on (0023) 66 73 34 91, Prof Samba Sow on (00223) 76348947, or CVD-Mali, CNAM, Ex-Institut Marchoux on (00223) 20 23 60 31.

To learn more about the ethical approval of this study or about your rights as a research subject, you can contact the Ethics Committee of USTTB at (00223) 2022 5277, Prof. Saïbou Maïga at (00223) 66750118 or Prof. Mahamadou Diakité (Permanent Secretary) at (00223) 66231191

Appendix 1b: Verbal Consent form for Village Participation

To be recorded electronically by a member of the LAKANA study team, on behalf of the head of the village chiefs, representatives or other persons authorised to speak on behalf of a village/grouping of hamlets

The giving and recording of consent should be confirmed by an independent witness

The persons named below affirm that they have given verbal consent and received information about the proposed trial or had this information explained to them, and that they consent to all eligible infants living in their village being enrolled in the trial. Witnesses to the consent process affirm that consent was given and information about the trial received according to the conditions laid out in the information about the trial.

Name of village

____

Name of Person authorised to give consent on behalf of village

____

Position / Role

Did the above-named person give consent? Yes/No Date

Name of Witness to Consent Procedures: ______________________________

(*If subject is illiterate, or otherwise unable to give consent)*

____

Witness's Confirmation Yes/No Date

Name of Investigator: ______________________________

or Authorized Representative obtaining informed consent

____

Investigator's Confirmation (initials or study code) Yes/No

Date

Appendix 2a: Information about the trial to a potential participant

(English version, to be translated into French and Bambara and read aloud to village leaders

CENTRE POUR LE DEVELOPPEMENT DES VACCINS, MALI

Director General: Samba Ousmane SOW, M.D., M.Sc., FASTMH

Professor of Medicine

Infectious Diseases Project: Measles Vaccine Initiative ♦ Enteric Disease Project ♦ Pneumococcus, Meningococcus, & *Haemophilus influenzae* Prevention Program
Malaria Project: Bandiagara Malaria Project ♦ Malaria Training Program

CVD-MALI

Large-scale Assessment of the Key health-promoting Activities of New mass drug administration regimens with Azithromycin (LAKANA)

Information for Participants and Informed Consent Forms for households

This document contains information for participants in a cluster-randomised, double-blinded, parallel group, controlled trial, to test the effects of mass drug administration of azithromycin on mortality and other outcomes among 1-11-month-old infants in rural Mali. It also contains the necessary consent forms for participants to enrol in the trial.

Key Investigators

Centre pour le Développement des Vaccins–Bamako, Mali

Per Ashorn, MD, PhD [1], Ulla Ashorn, PhD [1], Yin Bun Cheung, PhD [1,2], Camilla Ducker, MSc., MBBS [3], Nigel Klein, MBBS, PhD [4], Samba O. Sow MD., MSc., FASTMH [5].

Participating Academic Institutions

[1] Tampere University, Finland; [2] Duke-NUS Medical School, Singapore; [3] Tro Da Ltd, UK; [4] University College London, UK; [5] CVD-Mali, Bamako, Mali.

Study Overview

You are invited to participate in the LAKANA trial, in which we will try to determine if a drug called azithromycin can help Malian infants to enjoy better health and better rates of survival. In the study, some infants will receive azithromycin and others will receive something called a placebo, which looks and tastes like azithromycin but does not contain any medicine. The study will compare these different groups of children.

Azithromycin is an effective and safe medicine that is commonly used to treat illnesses – it has been used for many years to successfully and safely treat infections, including millions of cases of an eye infection called trachoma. There are studies that show that periodically using this medicine to treat healthy children in West Africa might reduce mortality. The LAKANA trial team is investigating if such treatment would also be beneficial to Malian infants.

In total, we plan to include 50–60 000 children in the study, from approximately 1150 villages or urban areas in the Kayes, Kita, and Koulikoro regions. If you agree to participate, the LAKANA team will visit you every three months over the next two years, to ask you some questions, and give study medicine to 1-11 month old infants living in your household.

Occasionally, children who take azithromycin may suffer stomach upsets for a short period, but otherwise we don't expect the treatment to cause any harmful side effects. Reactions, although rare, are always possible when medicines are given to children. So we will monitor the health of your child(ren) after they receive the medicine. In the unlikely case that your child experiences a negative reaction, CVD-Mali will help the child to receive medical care and cover any costs related to that care.

This study could lead to major improvements in the health of infants in Mali. By participating in this trial, you may be contributing to the development of better health programmes for the whole country. If this trial is successful, it is possible that azithromycin may be given to infants across Mali and West Africa.

You do not have to take part in this study – you are free to decide if you want to participate or not.

What is the study about?

Azithromycin is a type of medicine, an antibiotic, that is commonly used to treat infections, including eye infections that cause blindness. Recent research studies have shown that infants in certain African countries had better survival rates when they took azithromycin every six months, when compared to those who did not take the medicine. In the LAKANA trial, researchers from Centre pour le Développement des Vaccins-Mali (CVD-Mali) and their partners in Finland and the United Kingdom are studying how well azithromycin works in Mali. The trial is being funded by an American organization called the Bill & Melinda Gates Foundation.

The LAKANA trial will be carried out in 1150 villages or urban areas in the Kayes, Kita,

and Koulikoro regions, after local representatives have given permission for study activities to take place there. In each village or town area, the study team will visit all households every three months for a period of two years. The team members will ask for permission to record and store a list of household members. If there are any 1-11 month old infants in the household, the household will be offered the chance to participate in the study and the infants living there will be offered the study medicine. At subsequent visits, team members will monitor the effects of the study medicine by asking questions about the infants' health.

Two types of study mixture will be given to infants in the LAKANA trial: azithromycin and placebo. Placebo is something that looks and tastes like azithromycin but does not contain any active ingredient that can treat infection. In approximately one third of the study villages, all infants will receive azithromycin during all the study visits, every three-months. In another third of the study villages, infants will receive azithromycin at half of the visits and placebo at the other half. In the last third of the study villages, infants will always receive placebo.

Deciding which villages would be in which group was done by chance, like taking one single grain from a bag full of rice. During the study neither you nor anyone in the study team will know whether your infant receives placebo or active azithromycin at any of the home visits – this information will only be available later on to the study team. This way of carrying out the trial will help the study team to make correct conclusions about the health effects of azithromycin.

Study outcomes

The study will test to see if azithromycin has a positive impact on infant health and if it reduces infant mortality. The results of the study will be used to influence public health policy in Mali and in the wider sub-Saharan African region.

At approximately midway during the trial, researchers will analyze the obtained results. If there are clear indications that azithromycin is having a positive effect on mortality and the general health of infants, the trial will be modified, and azithromycin will then be given to all infants in the selected areas – and in other regions of Mali.

Who can take part in this study?

All households in the study area will be offered the chance to join the study for the purposes of collecting data. At each of the quarterly visits to households taking part in the study, all 1-11 month old infants will be offered the study medicine, as long as the infant has no known intolerance to azithromycin.

What will happen in the study?

If you take part in this study, the study team will visit you four times a year for two years (eight times in total, after an initial visit to collect information). At each visit, we will ask you some questions about your household and your infants' health and illnesses, we will measure the weight of your 1-11 month old infants, and will give them an appropriate dose (one teaspoonful or less) of study medicine. The medicine will come in the form of a mixture and will be placed in the infant's mouth using a disposable syringe.

Since the study visits will take place at three-month intervals and only 1-11 month old infants will be treated, the maximum number of treatments given to any individual infant will be four.

The first visit will take approximately 45 minutes of your time, the subsequent visits will likely take approximately 15 minutes each.

If you are not at home when the study team comes to see you, they will try to visit again later or they may call you to agree on a time when you will be at home. If for some reason the visit cannot be completed, you and your infant can still continue to take part in the study and be seen during subsequent home visits.

How do I give my consent for trial participation?

Before starting any trial-related activities in your home village, we will have received permission to conduct trial activities there from your village representatives. At your household, first of all, we will ask for permission to visit you and ask some questions every three months for the next two years. Additionally, on each visit, we will ask your permission to weigh your 1-11 month old infants and administer study medicine to them.

You don't need to sign any documents for your participation, but we will record your consent on our computer. We will also ask an independent person to witness your consent if you are not literate. His or her name will also be recorded in the computer system.

Is participation voluntary?

Your infant's participation in this study is entirely voluntary. You do not have to take part in this study, and you are free to leave the study at any time without giving a reason. Your infant's participation will not change anything about the health care your infant receives at any health facilities. If, for any reason, you are no longer available for follow-up visits, we will ask to contact you again to check on the health of your infant. You may refuse this as well. If you withdraw from this study, we will keep the information that we have already collected from your infant.

We will tell you about any significant new findings that are available during the study that may affect your willingness to involve your infant in the study.

Can my infant be removed from this study?

The study doctors and Malian, Finnish, or UK-based research committees overseeing the safety and rights of study participants, can remove you or your infant from the study or even stop the entire study without your approval if serious health problems are noticed. If you decide to stop your infant's participation in the study, or if your infant's participation is ended, the study doctor or study staff may ask you some questions about your infant's participation in the study. Answering any such questions is, however, voluntary.

What are my responsibilities if I take part in this study?

If you participate in this research, we will visit your household every three months and

ask you to inform us if you move or expect to be unavailable. If you give consent for your infant to participate in this study, you will still be responsible for all other non-study related health care for your infant.

What risks are involved in azithromycin use and trial participation?

Azithromycin is commonly used and is known to be safe for infants. Even so, a few people may react to azithromycin and experience brief episodes of diarrhoea, nausea, abdominal pain and vomiting. A very small number of children may also experience liver problems, rashes, itching, or swelling of the lips or throat when they take azithromycin.

In extremely rare cases, some young infants may experience a problem where they have trouble passing food from the stomach to the intestines, which can cause vomiting and may require surgery. This can happen to any infant, whether they take azithromycin or not. If you think your infant is experiencing a reaction like this or has developed other symptoms, please seek care urgently at the closest health facility and contact the study doctor.

If your infant falls ill because of the study treatment, CVD-Mali will pay for the costs of medical treatment according to Malian standards of care.

What are the possible benefits of being in this study?

There may be no direct benefits to you or your infant as a result of participating in this study. However, the information obtained in this study will be important in deciding if azithromycin could improve the health of infants in Mali and other similar countries.

What kind of feedback can I expect from research results?

We will know the results of the study after it is finished. The study team will hold meetings to inform the community about the results.

What are the costs of taking part in this study?

All research activities will be performed free of charge.

What are the payments for being in this study?

There are no payments for taking part in the study. But you and your infant will be visited every three months while the study is active, and you may signal other issues related to your infant's health to the *relais* and data collector who will visit you.

What are the alternatives to participating in this study?

Giving azithromycin to infants in Mali is not currently part of routine care. The alternative to participating in the study is choosing not to take part.

How will information be kept private?

To protect your privacy, we will keep the information collected by the study about your infant secure. We will only allow authorized people to see it, including the study team

from Mali, UK, and Finland, their representatives and the Ethics Committee in Mali, who act to protect people who take part in research. If study results are released to the public, the identities of participants will not be shared.

Who can answer questions about this study?

If you have any questions regarding your infant's participation or if your infant has a problem as a result of participating in this study, you may contact at any time Dr Fatoumata Diallo on (00223) 74 60 18 19, Dr Fadima Cheick Haidara on (0023) 66 73 34 91, Prof Samba Sow on (00223) 76348947, or CVD-Mali, CNAM, Ex-Institut Marchoux on (00223) 20 23 60 31.

To learn more about the ethical approval of this study or your rights as a research subject, you can contact the Ethics Committee of USTTB at (00223) 2022 5277, Prof. Saïbou Maïga at (00223) 66750118 or Prof. Mahamadou Diakité (Permanent Secretary) at (00223) 66231191.

Appendix 2b: Verbal Household Consent form for Trial Participation

(English version, to be translated into French and Bambara and read aloud to village leaders

To be recorded electronically by a member of the LAKANA study team, on behalf of the head of the household or other person authorised to give consent for household participation

The giving and recording of consent should be confirmed by an independent witness

The persons named below affirm that they have given verbal consent and received information about the proposed trial or had this information explained to them, and that they consent to all eligible infants living in the household being enrolled in the trial. Witnesses to the consent process affirm that consent was given and information about the trial received according to the conditions laid out in the information about the trial.

Name of household

____

Name of Person giving consent for household

____

Role in household

Did the above-named person give consent? Yes/No Date

Name of Witness to Consent Procedures: ______________________________

(*If subject is illiterate, or otherwise unable to give consent)*

____

Witness's Confirmation Yes/No Date

Name of Investigator: ______________________________

or Authorized Representative obtaining informed consent

____

Investigator's Confirmation (initials or study code) Yes/No
Date

Appendix 3: Consent to provide study medicine to an individual infant

(English version, to be translated into French and Bambara and read aloud to village leaders

For **Parent/Guardian aged 18 years or older or married Parent/Guardian aged 16 years or older**
[If parent or guardian is younger than 18 and unmarried, or younger than 16 years of age, skip to next page]

The persons named below affirm that they have given verbal consent and received information about the proposed trial or had this information explained to them, and that they consent to the infant or infants in their care being enrolled in the trial, to receive study medicine and for information about these infants to be collected by the trial team. Witnesses to the consent process affirm that consent was given and information about the trial received according to the conditions laid out in the information about the trial.

____

Name of Household & identifier for Infant receiving study medicine

____

Name and Role of Person giving consent

Did the above-named person give consent? Yes/No
Date

Name of Witness to Consent Procedures: _______________________________
(*If subject is illiterate, or otherwise unable to give consent)*

____

Witness's Confirmation Yes/No
Date

Name of Investigator: _______________________________
or Authorized Representative obtaining informed consent

____

Investigator's Confirmation (initials or study code) Yes/No
Date

Appendix 3: Consent to provide study medicine to an individual infant
(English version, to be translated into French and Bambara and read aloud to village leaders

Informed Consent Form for **Parents/Guardian of participants younger than 16 years old, or unmarried participants younger than 18 years old**

The persons named below affirm that they have given verbal consent and received information about the proposed trial or had this information explained to them, and that they consent to the infant or infants in their care being enrolled in the trial, to receive study medicine and for information about these infants to be collected by the trial team. Witnesses to the consent process affirm that consent was given and information about the trial received according to the conditions laid out in the information about the trial.

____

Name of Household & identifier for Infant receiving study medicine

____

Name and Role of Person giving consent

Did the above-named person give consent? Yes/No
Date

Name of Witness to Consent Procedures: ________________________________
(*If subject is illiterate, or otherwise unable to give consent)*

____

Witness's Confirmation Yes/No
Date

Name of Investigator: ________________________________
or Authorized Representative obtaining informed consent

____

Investigator's Confirmation (initials or study code) Yes/No
Date

Appendix 4a: Information on secondary outcome data collection

(English version, to be translated into French and Bambara and read aloud to village leaders

CENTRE POUR LE DEVELOPPEMENT DES VACCINS, MALI

Director General: Samba Ousmane SOW, M.D., M.Sc., FASTMH

Professor of Medicine

Infectious Diseases Project: Measles Vaccine Initiative ♦ Enteric Disease Project ♦ Pneumococcus, Meningococcus, & *Haemophilus influenzae* Prevention Program
Malaria Project: Bandiagara Malaria Project ♦ Malaria Training Program

CVD-MALI

Large-scale Assessment of the Key health-promoting Activities of New mass drug administration regimens with Azithromycin (LAKANA)

Information about Participation in the Anti-Microbial Resistance and other sub-studies and Informed Consent Forms for individuals

This document contains information for participants in a cluster-randomised, double-blinded, parallel group, controlled trial, to test the effects of mass drug administration of azithromycin on mortality and other outcomes among 1-11-month-old infants in rural Mali. It also contains the necessary consent forms for participants to enrol in the trial.

Key Investigators

Centre pour le Développement des Vaccins–Bamako, Mali

Per Ashorn, MD, PhD [1], Ulla Ashorn, PhD [1], Yin Bun Cheung, PhD [1,2], Camilla Ducker, MSc., MBBS [3], Nigel Klein, MBBS, PhD [4], Samba O. Sow MD., MSc., FASTMH [5].

Participating Academic Institutions

[1] Tampere University, Finland; [2] Duke-NUS Medical School, Singapore; [3] Tro Da Ltd, UK; [4] University College London, UK; [5] CVD-Mali, Bamako, Mali.

Protocol version 7.0: 13. November. 2024

Introduction

Thank you for participation in the LAKANA trial, which will try to determine if a drug called azithromycin can help Malian infants to enjoy better health and better rates of survival.

You will have already received information about the trial and have given consent for the trial team to ask questions about the health of the child/children in your care and for us to give them trial medicine (azithromycin or placebo).

If you would like more information about the trial or be reminded of its key aims, please speak to the members of the trial team or the *relais communautaire* who visit your village or the study team.

LAKANA sub-studies

The main LAKANA study will be implemented in approximately 1150 villages or town areas in Kayes. In some of the villages, there will be additional sub-studies, in which we will collect further information on the effects and feasibility of azithromycin distribution. Your village and others around the CSComs of Kati, Bendougouba, Dafela, Djidian, Kofeba and Koulikoro have been selected for the sub-studies. Therefore, we would like to describe them to you now and ask if you are in principle willing to participate or allow your child to participate in some of them.

In total, there will be four sub-studies that will be completed over the next 2-3 years: one on infant growth, second on the possible development of antimicrobial resistance, third on the mechanisms how azithromycin works and fourth on the feasibility of distributing azithromycin to populations in Mali. No child will be invited to participate in these sub-studies more than once or twice. Later, when we are about to do any of these sub-studies, we will ask your permission again.

Infant growth sub-study

In some earlier studies, antibiotic treatment has improved child growth. Therefore, we would also like to study if infants who receive azithromycin are bigger than infants who receive placebo.

To measure growth, we will at certain home visits invite all 6-8 month-old and 12-14 month old children to a nearby health centre or central location in village. At the health centre/central location, we will measure the child's weight, length, and arm. Growth measurement is harmless, although some infants cry a bit when their length is taken. This visit will take approximately 30 minutes plus your travel time to the location.

The Antimicrobial resistance (AMR) sub-study

When antibiotics are used, bacteria sometimes become resistant to them, leading to reduced options for treatment of children or adults with infections. In order to judge if this happens with azithromycin in Kayes, the trial team will conduct an antimicrobial

resistance (AMR) sub-study in your village.

To measure AMR, the study team will at selected home visits collect swab samples from children who are 4-14 month-old or 49-59 month-old. The samples will be collected with a thin cotton swab from the nose and rectum of the child, at your home or a central location of your village. The procedure is harmless and takes only some minutes, although swabbing the nose may sometimes feel a bit uncomfortable and some children may cry a bit. Any irritation is, however, mild and transient.

The AMR samples will be collected on four of the home visits that the LAKANA team will make: when the household joins the study and at 12, 24, and 36 months thereafter. Since the samples are taken only from children who are at the right age-bracket at these visits, the same child can provide samples only once and we will ask for your permission each time before taking any samples.

The collected samples will be stored and analysed at CVD-Mali laboratory in Bamako. Selected samples will be shipped for further analyses in collaborating centres in UK, Finland and possibly elsewhere. All samples will be coded and stored anonymously.

How azithromycin works

In order to better understand how azithromycin works in infants, the study team will carry out two sets of data collections.

At the fourth study visit, before provision of the study medicine, the study team will take a small blood sample with a finger-prick or heel-prick from all 4-11 month-old infants, at a central location of your village. The team will also collect a stool sample from the same infant. Two weeks later, you will be asked to bring the infant to a nearby health facility, for collecting another finger-prick or heel-prick blood sample and a stool sample. At this second visits, there will also be an interview about the health of the infant after she received the study medicine.

Later, at selected home visits, we will invite 6-8 month-old and 12-14 month-old infants to be brought to a health centre or another central location in the village. At the health centre or central location, a study nurse will collect a small blood sample (less than a tea-spoon) from the child's arm. Before the visit, you will be given a disposable diaper and a small plastic container. We will ask you to place the disposable diaper on the child on the morning of the health centre visit and the study team will collect a urine sample from it. In the container, we will ask you to collect a sample of the child's stool and bring it to the health centre. There will also be an interview about the health of the infant after she received the study medicine.

The health facility/central location visits will take approximately 45 minutes plus your travel time. The procedures during these visits are harmless, but blood sampling can cause some discomfort and the child may have mild tenderness and bruising following the blood being drawn. Very rarely children can feel lightheaded for a short period of time.

Since the samples are taken only from children who are at the right age-bracket at selected visits, the same child can provide samples only once or at maximum twice and we will ask for your permission each time before taking any samples.

The collected samples will be stored and analysed at CVD-Mali laboratory in Bamako.

Selected samples will be shipped for further analyses in collaborating centres in UK, Finland and possibly elsewhere. All samples will be coded and stored anonymously.

Acceptability and feasibility of azithromycin distribution

The study will also try to answer questions about the acceptability and feasibility of treating infant with azithromycin. In order to do so, the study team will ask questions about these themes during selected home visits. Questions will be about your infant's health, about the local health system and about the acceptability of azithromycin to your community, for example. Your answers will help to shape the outcomes of the study.

These additional questions will be asked at your home, by specifically trained study personnel. In total, there will be three interviews and each of them will take approximately 30 minutes. Your answers will be entirely confidential and will not be used to identify you or your family in any way.

Is participation voluntary?

Your infant's participation in these sub-studies is entirely voluntary. You do not have to take part in these, and you are free to leave them at any time without giving a reason.

What are the possible benefits of being in these sub-studies?

There may be no direct benefits to you or your infant as a result of participating in these sub-studies. However, the information obtained in these sub-studies will be important in deciding if azithromycin could improve the health of infants in Mali and other similar countries.

What kind of feedback can I expect from research results?

We will know the results of the sub-studies when the trial has finished. The study team will hold meetings to inform the community about the results.

What are the alternatives to participating in these sub-studies?

These sub-studies are an addition to the main azithromycin trial and are not a routine part of the trial's drug treatment. They are not either a part of routine health care. The alternative to participating in these sub-studies is choosing not to take part. Not participating in this sub study does not affect your right to participate in the main Azithromycin trial.

How will information and biological samples be kept private?

To protect your privacy, we will keep the information collected by the sub-studies about your infant secure. We will only allow authorized people to see it, including the study team from Mali, UK, and Finland, their representatives and the Ethics Committee in Mali, who act to protect people who take part in research. If study results are released to the public, the identities of participants will not be shared.

Biological samples taken from your child will also be stored securely at the CVD laboratory in Mali. All samples will be coded and stored anonymously.

Who can answer questions about these sub-studies?

If you have any questions regarding your infant's participation or if your infant has a problem as a result of participating in this study, you may contact at any time Dr Fatoumata Diallo on (00223) 74 60 18 19, Dr Fadima Cheick Haidara on (0023) 66 73 34 91, Prof Samba Sow on (00223) 76348947, or CVD-Mali, CNAM, Ex-Institut Marchoux on (00223) 20 23 60 31.

To learn more about the ethical approval of this study or your rights as a research subject, you can contact the Ethics Committee of USTTB at (00223) 2022 5277, Prof. Saïbou Maïga at (00223) 66750118 or Prof. Mahamadou Diakité (Permanent Secretary) at (00223) 66231191.

Appendix 4b: Consent form for Participation in LAKANA Sub-studies

(English version, to be translated into French and Bambara and read aloud to village leaders

For **Parent/Guardian aged 18 years or older or married Parent/Guardian aged 16 years or older**

[If parent or guardian is younger than 18 and unmarried, or younger than 16 years of age, skip to next page]

The persons named below affirm that they have given written consent and received information about the proposed LAKANA sub-studies or had this information explained to them, and that they consent to all eligible infants in their care being enrolled in the sub-studies. This includes consenting to the trial team taking all necessary biological samples. Witnesses to the consent process affirm that consent was given and information about the sub-studies received according to the conditions laid out in the trial information.

I am consenting to:

1. Participation in the growth sub study ☐
2. Participation in the antimicrobial resistance sub study ☐
3. Participation in the mechanisms sub study ☐
4. Participation in the acceptability and feasibility sub study ☐

____

Printed name of participant

____

Printed name of participant's parent/guardian

____

Signature or Fingerprint of Participant's Parent/Guardian Date

Name of Witness to Consent Procedures: ________________________________

(*If subject is illiterate, or unable to sign)*

____

Witness's Signature Date

Name of Investigator: ____

or Authorized Representative obtaining informed consent

____

Investigator's Confirmation (initials or study code) Yes/No
Date

Appendix 4b contd: Consent form for Participation in LAKANA Sub-studies
(English version, to be translated into French and Bambara and read aloud to village leaders

Informed Consent Form for **Parents/Guardian of participants younger than 16 years old, or unmarried participants younger than 18 years old**

The persons named below affirm that they have given written consent and received information about the proposed LAKANA sub-studies or had this information explained to them, and that they consent to all eligible infants in their care being enrolled in the sub-studies. This includes consenting to the trial team taking all necessary biological samples. Witnesses to the consent process affirm that consent was given and information about the sub-studies received according to the conditions laid out in the trial information.

I am consenting to:

1. Participation in the growth sub study ☐
2. Participation in the antimicrobial resistance sub study ☐
3. Participation in the mechanisms sub study ☐
4. Participation in the acceptability and feasibility sub study ☐

____

Printed name of participant

____

Printed name of participant's parent/guardian

Signature or Fingerprint of Participant's Parent/Guardian ____________ Date

Name of Witness to Consent Procedures: ________________________

(*If subject is illiterate, or unable to sign)*

____

Witness's Signature Date

Name of Investigator: ____

or Authorized Representative obtaining informed consent

____

Investigator's Confirmation (initials or study code) Yes/No
Date

Appendix 5, referral letter for asymptomatic children with abnormal laboratory values observed during a village visit

(English version, to be translated into French)

Referral of a study child to CSCOM

Date: ____________________________

Child name: ____________________________

Child weight: ____________________________ kg

Home village: ____________________________

LAKANA study ID: ____________________________

Blood Hb concentration (g / dl): ____________________________

Blood CRP concentration (mg / l): ____________________________

Please be advised that members of the LAKANA study team have today seen this child and have measured her / his blood concentration of haemoglobin (Hb) and C-reactive protein (CRP). We have observed some abnormal laboratory values[1] in these tests and are referring these children for your assessment. Below, we have listed the observed abnormality and our recommended action for the case (based on recommendations made in the Pocket Book of Hospital Care for Children, World Health Organization, 2013

☐ **Hb below critical threshold[1]. The child has severe anemia.**
Test for malaria with a rapid test. Treat for malaria, if the test is positive.
Perform blood transfusion if possible.
Follow other Malian guidelines for treatment of severe anemia.

☐ **Hb values in critical range[1]. The child has moderate anemia.**
Test for malaria with a rapid test. Treat for malaria, if the test is positive.
If malaria test is negative, provide iron syrup (3 mg / kg) for 3 months.
Advise the caregiver about good feeding practice.

☐ **CRP at or above critical threshold[1]. The child has systemic inflammation and possibly an infection**
Test for malaria with a rapid test. Treat for malaria, if the test is positive.
Assess for symptoms indicative of symptoms that might warrant further diagnostics.
If malaria test is negative and no other clues, provide amoxycillin syrup (20 mg / kg twice daily), for 5 days.

We appreciate your collaboration and assistance to the child. If you have any questions, please do not hesitate to contact us.

____

On behalf of the LAKANA study team
Prof. Samba Sow
Contact details

[1] *According to references from the Mali national and local guidelines in use.*
