# Supplementary material for: A single dose of azithromycin versus placebo for malaria in infants: A cluster-randomized trial
Source: PLOS Glob Public Health. 2026 Aug 20;6(8):e0006518. doi: 10.1371/journal.pgph.0006518 (PMC13492813; doi:10.1371/journal.pgph.0006518)
Supplement: Supplementary file 4 [file pgph.0006518.s005.docx]

Alassane Dicko
Academic Editor
PLOS Global Public Health

Dear Dr. Alassane Dicko and Reviewers,

We are grateful for the constructive and insightful feedback received from reviewers and the editorial team for the manuscript: **A single dose of azithromycin versus placebo for malaria in infants: A cluster-randomized trial.**

We have reviewed the comments carefully and revised the manuscript accounting for each comment. Detailed point-by-point responses and the corresponding changes made to the manuscript are provided below. Reviewer and editorial comments are presented in black font, our responses in blue font, and quoted pieces of the manuscript in green italics font with line numbers specified. Line numbers refer to the revised manuscript. Both a tracked and a clean version of the revised manuscript are uploaded in the submission system.

We believe these changes have clarified the manuscript’s narrative and presentation, and we thank the Reviewers and editorial team for their valuable comments. We hope that you would consider this revised manuscript for publication in the *PLOS Global Public Health*.

Sincerely,

Rikhard Ihamuotila on behalf of the LAKANA malaria study team

**Journal Requirements:**

1. Please ensure that your Ethics Statement is available in its entirety at the beginning of your Methods section, under a subheading 'Ethics Statement'.

Our response: Edited as suggested. The ethics paragraph along with the informed consent paragraphs were moved to the beginning of Methods under the suggested new subheading ‘Ethics statement’. Additionally, the date of the most recent IRB reapproval was updated to 13 October 2025 (lines 95-96)

2. We ask that a manuscript source file is provided at Revision. Please upload your manuscript file as a .doc, .docx, .rtf or .tex.

Our response: Noted and uploaded as docx.

3. We do not publish any copyright or trademark symbols that usually accompany proprietary names, eg (R), (C), or TM  (e.g. next to drug or reagent names). Please remove all instances of trademark/copyright symbols throughout the text, including "Orion QuickRead go®" on page 7.

Our response: Edited as suggested. All trademark/copyright symbols are now removed.

4. We have noticed that you have uploaded Supporting Information files, but you have not included a list of legends. Please add a full list of legends for your Supporting Information files after the references list.

Our response: Edited as suggested. A Supporting Information section has been added after the reference list with the legends (lines 600-612).

*“SUPPORTING INFORMATION*

*CONSORT checklist*

*LAKANA original protocol*

*LAKANA latest protocol”*.

********************************************************

**Response to Reviewer 1**

Reviewer #1: This study examines azithromycin treatment in a CRT substudy, hypothesizing that azithromycin reduces malaria prevalence, parasite load, and improves hemoglobin concentration. However, the analyses are not presented clearly enough to indicate the expected statistical power with the current sample size. Ultimately, no results reach statistical significance, and the manuscript appropriately acknowledges the insufficient power.
Additional details are needed regarding the regression analyses in the statistical methods. The tables and figures are well designed.

More specific comments below.

Abstract: The conclusion of the abstract should be revised. I do not agree with the statement that the results are consistent with the hypothesis.

Our response: This concern is understandable since the prospective per-protocol hypotheses tested had no statistically significant results. This is also the reason why the Abstract conclusion starts with “*The findings do not conclusively support… [the hypothesis]”.* However, given that all tested malaria outcomes were lower with azithromycin together with the lower than expected statistical power, we still consider the conclusion wording valid: *“…but are consistent with, a hypothesis that a single dose of azithromycin reduces malaria prevalence and parasite load…”.*

Strengthening our conclusion is the post-hoc finding that among those who were malaria PCR-positive at baseline, parasite load was 85% lower with azithromycin (p<0.001). Therefore, after careful consideration within the team, we opted with the original conclusion and would prefer not to change it at this point.

Additionally, this same concern is discussed in the second paragraph and the limitations paragraph of the Discussion, leading to the same conclusion as is presented in the Abstract.

We suggest to keep the Abstract conclusion as it is (lines 30-32): *“The findings do not conclusively support, but are consistent with, a hypothesis that a single dose of azithromycin reduces malaria prevalence and parasite load among infants in a malaria-endemic West African setting.”*

Methods

Lines 96-98: The statement that data were collected during a single MDA should be clarified as it was the fourth round of a set of MDA. This part requires clarification.

Our response: Edited as suggested. The sentences were changed to further clarify during which MDA the substudy was conducted in the context of the parent trial.

The text now reads (lines 120-122):

“*The villages were visited every three months in the LAKANA trial, and the data collection for this substudy happened on the fourth MDA round, nine months after village enrollment.”*

Effect size 0.2-0.3 SD. Since three different outcomes are reported, specify which effect size refers to each outcome.
This also requires clarification.

Our response: Edited as suggested.

1. We changed the wording (line 217) to *“…offered an* ***expected*** *statistical power of 80%...”* to clarify that it was a prospective expected sample size calculation as this was a prospectively designed trial. There is also a reference to the prospectively published statistical analysis plan (ref 31) from where this sample size calculation section is derived. The actual sample size after data collection is presented in the Results section and the participant flow (Figure 1). The smaller than expected sample size is also discussed in the second and limitations paragraphs of the Discussion.
2. To clarify that the detection cut-off (0.2-0.3 standard deviations) for the power calculation effect size was for all outcomes, we added the wording *“for each outcome*” (line 219) and replaced the SD abbreviation with “*standard deviation*” to prevent misunderstanding (line 218).

Did the pilot study provide estimates for baseline and mean parasite load?

Our response: There was no pilot study, but only a pilot village in which substudy activities were tested. The pilot village sentence (line 220-222) was edited to clarify why it was not used in the analyses. The sentence now reads: *“One substudy village served as a pilot village for testing substudy data and sample collection and was therefore excluded from analyses.”*

As this is a substudy, the Methods section should report the cluster size for each arm.
Also, it may be best to evaluate the expected power using the known cluster sizes, number of clusters, baseline prevalence, and other relevant measures. Data from the pilot study could be used for these estimates.

Our response: Unfortunately, there was no pilot study, only one pilot village which was used to test substudy data and sample collection. Therefore, we could not have known in advance the cluster size for each arm nor the baseline prevalence. The Methods sample size calculations are only prospectively expected before any data collection for the parent trial began, adhering to prospective design standards. The eventual sample size is reported in Results, and the smaller than expected sample size is then discussed in the second and limitations paragraphs (lines 314-326 and 364-373) of the Discussion section.

Statistical analysis: Logistic regression was used to model outcome prevalence, but the analytic approach for parasite load and hemoglobin concentration should also be specified clearly in the Methods section.
Our response: Edited as suggested. The mixed-effects model sentence (lines 227-228) has now an added specification for the continuous variables: *“… (logistic model for prevalence and linear models for continuous variables)”.*

Results

Graphics and tables are well prepared. The results indeed do not indicate significant effects.
Our response: Thank you, we appreciate the careful review of the graphics.

Discussion

Lines 277-279: This statement is speculative and should be removed from the discussion.

Our response: Edited as suggested. The statement was removed.

********************************************************

**Response to Reviewer 2**

Reviewer #2: This manuscript describes findings from a sub-study nested within a cluster randomized controlled trial in Mali, where the sub-study aimed to assess if azithromycin mass treatment for infants has any effect on malaria prevalence and mean parasitemia in infants. The study was intended to assess if changes in malaria burden are an intermediate factor which is attributable for changes in all cause mortality observed in other studies after azithromycin mass treatment. However, since no effect of azithromycin on ACCM was observed in the main trial, it is perhaps not surprising that no significant effect of azithromycin on malaria was observed. Nevertheless, this research merits publication, is generally well-designed and presented, and contributes to the evolving evidence base on this theme.

1. Line 50. I recommend modifying here and throughout the rest of the paper to clarify between “malaria” (generally used as an indication of clinically-evident or symptomatic disease) and Plasmodium infection (a more specific description of the outcomes actually presented here).

Our response: Edited as suggested by using the term “PCR-positive malaria”. Edited on original line 50 (now line 52) and throughout the manuscript where feasible or unclear. Added the PCR-positive malaria specification to line 55 in Authors summary, and beginning of Results (on line 251) to ensure that readers clearly understand malaria positivity as PCR-positivity thereafter. Abstract now states PCR-positive malaria on lines 20 and 24. Added *“PCR-positive malaria”* to Discussion first paragraph (line 312). The Table 1 and 2 “general prevalence of malaria” was changed to *“Prevalence of PCR-positive malaria”.*

Additionally, the Methods-Outcomes section specifies malaria as PCR-detected parasitemia (line 192). Lines 208-209 also explain that: *“Samples were considered positive if both had a cycle threshold (Ct) value of < 38*.”

Given all these specifications, we feel it is now more precise throughout the manuscript and clear that it is not clinical malaria (or clinical Plasmodium infection), but simply unspecified PCR-positive malaria.

2. Line 81. This sentence seemed to directly contradict the previous paragraph, where you have described multiple other trials of azithromycin with malaria-related outcomes.

Our response: The end of the referred sentence specifies the WHO-recommended infant subgroup, in which evidence is indeed scant (no known azithromycin MDA studies testing the age group specifically when systematically searching databases). Suggest keeping as it is (lines 85-86): *“Evidence is scant on the effect of azithromycin MDA on malaria prevalence and parasite load in the* ***WHO-recommended infant subgroup****.”*

3. Line 89. It may be informative for readers who are not familiar with the LAKANA trial to state here or in the next paragraph if this substudy was completed in all trial clusters, or only a subset. Line 128 is also slightly ambiguous and hints that this study was done in a subset of the most accessible clusters.

Our response: Edited as suggested for original line 89. It now reads on line 108: *“This cluster-randomized placebo-controlled trial was conducted as a prespecified substudy in a subset of villages within a large-scale trial in Western Mali testing the effect of azithromycin MDA on infant mortality”*

For original line 128 (now 161), we suggest keeping as it is, because the Reviewer has understood it correctly: these villages needed to be closer to Kita laboratory, as farther away villages can be extremely hard to reach in Mali and would risk compromising particularly temperature sensitive substudy biological samples.

4. Line 184. It is not clear which outcome was used for the sample size calculation. What was the anticipated prevalence of P. falciparum by PCR in this population?

Our response: Thank you! A similar point was raised by the previous Reviewer.

This section is now clarified to simultaneously address a sample size clarification request from the previous Reviewer:

1. We changed the wording (line 217) to *“…offered an* ***expected*** *statistical power of 80%...”* to clarify that it was a prospective expected sample size calculation. There is also a reference to the prospectively published statistical analysis plan (ref 31) from where this sample size calculation section was derived for all substudy outcomes (not only malaria or Hb).
2. The actual sample size after data collection is presented in the Results section and the participant flow (Figure 1), and the smaller than expected sample size is discussed in the second and limitations paragraphs of the Discussion (lines 314-326 and 364-373 respectively).
3. We added the wording *“for each outcome*” (line 219) to clarify that the detection cut-off (0.2-0.3 standard deviations) for the power calculation effect size was for all outcomes.
4. The Discussion section outlines that we anticipated a malaria prevalence of something around 13-22% and discusses the sample size limitations in the second and limitations paragraphs (lines 314-326 and 364-373 respectively) of the Discussion.

5. Lines 190-192. Presumably the mixed-effects model used individual level data, with the outcome being positive/negative status by PCR? I would argue that if this is the case, it may be clearer to describe the outcome as “P. falciparum infection by PCR” rather than prevalence which is an aggregate measure at the cluster level. Furthermore, I assume the model uses the PCR result at the 14-day follow-up as the outcome, and the PCR result at baseline as a covariate? This detail would be helpful to include.

Our response: Edited to clarify the model by adding more description of the regression model and by clarifying that prevalence means prevalence of PCR-positivity:

1. Now on lines 228-230: *“The independent variables were baseline outcome measures (PCR-positivity, parasite load, or Hb) and intervention group.”*
2. The Methods-Outcome section already specifies prevalence of PCR-positivity as the outcome and now reads on line 192: *“The outcomes were malaria prevalence (PCR-detected parasitemia)”.* Additionally, we have clarified throughout the manuscript that malaria positive means only malaria PCR-positive (assessed earlier in Reviewer comment 1).

6. Table 1. I believe that the notation for the Mean parasite load should be 10^-3 ng/ul, not 10^3. Please check this throughout the paper.

Our response: Thank you noticing this mistake. Edited as suggested throughout the manuscript.

7. Methods/results. Please include a statement somewhere in these sections describing the dates of data collection, and how these dates relate to the usual seasonal transmission period in Mali. The low SMC coverage suggests that data collection was done during the dry season?

Our response: Edited as suggested. We added the specification: *“dry season”* on line 119*.* The specific dates of data collection are already reported on line 119: (February 17 to May 30, 2022).

8. Line 236. Did you collect data on how many of the children with severe anemia that were referred to the health center actually completed this referral, and if this referral rate differed between arms? This is a potential bias in your interpretation of azithromycin effects on Hb.

Our response: Unfortunately, we do not have data on how many of the children with severe anemia or severe illness actually completed the referral, and as proposed, it poses a potential bias for Hb but also for malaria outcomes.

Nevertheless, the referral rate due to severe anemia was 8.1% in azithromycin and 8.8% in placebo group. Among the infants with severe anemia, the loss to follow-up rates were 18% and 17% respective. Therefore, we deem the risk of bias minor.

9. Table 2. I’m not sure what is meant by “general” prevalence of malaria. Suggest changing this to “baseline prevalence of P. falciparum by PCR”. Similarly, I recommend modifying “prevalence of malaria among baseline malaria positives” to something like “Day 14 prevalence of P. falciparum by PCR among those PCR-positive at baseline”.

Our response: Edited as suggested. States now PCR-positive malaria throughout the manuscript in text and tables (assessed thoroughly earlier in Reviewer 2 comment 1).

10. Lines 255-256. This model result is very surprising considering the descriptive results reported earlier in this paragraph, which state that the mean parasite density among PCR positives is essentially unchanged in the azithromycin group (0.0366 at baseline, 0.0363 at day 14) while the parasite densities reduced in the placebo arm among malaria positives (0.0642 at baseline, 0.0510 at day 14). I may have misunderstood the data which is being used in the model, but this indicates some further explanation is required to ensure to either explain how the results in the adjusted model are so different from the descriptive analysis, or explain if there is a different population assessed in the model.

Our response: This is because the manuscript sometimes originally reported geometric means and sometimes arithmetic means for the highly skewed parasite load variable. Log-transformation produces only geometric means ratios. We now clarified in the Statistical analyses (lines 238-240) that the outcome was expressed as ratio of geometric means: *“Because parasite load data distributions were highly skewed, we reported geometric means for parasite load and used log-transformed parasite load values in the regression model.”*

Results section text numbers for parasite load is now also changed to express geometric means (lines 253-254 and 275-276): *“Mean parasite load at baseline was 0.0056* *ng/μL (geometric mean 0.0000089 ng/μL) in the control group and 0.0036 ng/μL (geometric mean 0.0000101 ng/μL) in the azithromycin group,”.*

The geometric means ratio is now also clarified in Table 2.

By reporting both means (arithmetic and geometric) in the Results section, there should not be a misunderstanding anymore.

11. Line 266. It is difficult to interpret or agree with this statement since the assumed prevalence is not reported in the sample size section, suggesting that prevalence was not a contributing factor in the calculation.

Our response: Clarified the Discussion paragraph related to lower than expected statistical power (lines 314-326). The lower than expected number of cases (almost half lower) affects also the interpretation of parasite load, which the paragraph also discusses. The referred paragraph (lines 314 to 319) now reads: *“These null results are likely because the study was underpowered, due to a lower-than-expected malaria prevalence and subsequently a smaller than expected number of malaria cases. At the time of the design of this LAKANA substudy, the 2018 Demographic Health Survey of Mali reported a 13 % prevalence of malaria in Kayes region and 22 % in neighboring Koulikoro region among 6-59 months old children [40]. In our sample, the baseline prevalence was clearly lower, at around 9%.”*

The Reviewer is right in that the expected prevalence was not a factor in prospective power calculations. This is because the prospective power calculations were meant to cover all possible substudy outcomes (malaria prevalence, load, Hb, and more substudy outcomes beyond this study).

12.Lines 274. As with my earlier comment, the 85% reduction in parasite load among baseline positive infants does not seem to agree with description of the results, suggesting substantial confounding or imbalance that is being accounted for in the adjusted model.

Our response: Please see the response to Reviewer 2 comment number 10, where the clarification of reporting geometric means is outlined. The manuscript now more consistently reports both arithmetic and geometric means to prevent misunderstanding.

13. Lines 298-306. This may be a suitable place to discuss whether exclusion of children identified as severely ill from trial was a potential limitation. My expectation is that for studies with ACCM as the outcome, these most vulnerable children are the ones that would benefit most from this kind of intervention, whether the effect is mediated through malaria parasitemia reduction or some other mechanism. Did these trials all exclude severely ill children, similar to the LAKANA trial?

Our response: Yes, this substudy excluded severely ill children because it was nested within the main LAKANA trial and followed its eligibility criteria. This is outlined in the last sentence of the Methods-Eligibility section (lines 165-166): *“Severely ill infants were excluded and referred to the nearest health facility.”*

As for the point that possibly missing severely ill children could be a potential limitation, we unfortunately do not have data on how many were excluded in the village due to being severely ill or how many actually went to the nearest health center after referral. We have thought about this possible limitation, but in the LAKANA main trial (with 149 000 infants) it was very rare to be excluded due this reason, so we opted with not taking this up as a limitation.

14. Lines 318. The statement “ongoing seasonal malaria outbreak” isn’t clear. Does this mean a usual seasonal malaria transmission setting, but during the seasonal peak (implying this study was done in the low transmission season)? Or does this relate to an epidemic, where transmission accelerates beyond the usual expected level?

Our response: Clarified now to mean a rainy season setting (lines 371-373). Clarified also in Methods that the data was collected during dry season (line 119). The referred sentence now reads (lines 371-373): *“Our results cannot be generalized to settings with an ongoing rainy season malaria outbreak, extensive SMC coverage, or when older children are also treated.”*

15. Line 323. I don’t agree that you have definitively shown that the reason for the null result was the low prevalence of malaria. It seems that azithromycin did not have a substantial effect on clearing parasites among those who were infected, which aligns with the Cochrane review showing that azithromycin has only a weak antimalarial effect.

Our response: Noted and well-argued. We removed from this sentence the part *”due to low prevalence of malaria”* and added “*possibly due to a low number of malaria cases”* instead. The sentence now reads (lines 375-378): *“We observed a slightly lower malaria prevalence and parasite load with a single dose of azithromycin, but the results were not statistically significant, possibly due to a low number of malaria cases.”*

********************************************************

**Response to Reviewer 3**

Reviewer #3:

1. Question: was a sensitivity analysis done regarding the missing village?

Our response: The missing village had only 1 participant, who was lost to follow-up with the whole village subsequently lost to follow-up for malaria. Therefore, no sensitivity analysis was made.

2. What are the restrictions on data availability for the repository? I do not find anything unreasonable about the way the data are being provided.

Our response: We have added clarification to the Data Sharing Statement which now reads (lines 417-423):

*“Data is available in Zenodo in a public repository with restricted access (https://doi.org/10.5281/zenodo.19470319). Access to the dataset is restricted because open data sharing was not included in the study information sheet at the time of data collection. Data will be made available to users who submit a written request outlining the proposed use, the justification, and a data use plan. Requests should be sent to the LAKANA Trial Principal Investigators at lakana.psg@lists.tuni.fi.”*

********************************************************

Figure Resubmissions:

Our response: The figures have been converted to TIFF with the NAAS tool as suggested.
