# Supplementary material for: A single dose of azithromycin versus placebo for malaria in infants: A cluster-randomized trial
Source: PLOS Glob Public Health. 2026 Aug 20;6(8):e0006518. doi: 10.1371/journal.pgph.0006518 (PMC13492813; doi:10.1371/journal.pgph.0006518)
Supplement: Peer review history

# Author comment: Author response to Decision Letter 0

**DOI:** 10.1371/journal.pgph.0006518.r001

**Transfer Alert**

This paper was transferred from another journal. As a result, its full editorial history (including decision letters, peer reviews and author responses) may not be present.

5 May 2026

# Peer review: Decision Letter 0

**DOI:** 10.1371/journal.pgph.0006518.r002

**Authors:** Alassane Dicko

30 Jun 2026

PGPH-D-26-01585

A single dose of azithromycin versus placebo for malaria in infants: A cluster-randomized trial

PLOS Global Public Health

Dear Dr. Ihamuotila,

Thank you for submitting your manuscript to PLOS Global Public Health. After careful consideration, we feel that it has merit but does not fully meet PLOS Global Public Health’s publication criteria as it currently stands. Therefore, we invite you to submit a revised version of the manuscript that addresses the points raised during the review process.

Please submit your revised manuscript by Jul 30 2026 11:59PM. If you will need more time than this to complete your revisions, please reply to this message or contact the journal office at globalpubhealth@plos.org. When you're ready to submit your revision, log on to https://www.editorialmanager.com/pgph/ and select the 'Submissions Needing Revision' folder to locate your manuscript file.

Please include the following items when submitting your revised manuscript:

- A letter that responds to each point raised by the editor and reviewer(s). You should upload this letter as a separate file labeled 'Response to Reviewers'.
- A marked-up copy of your manuscript that highlights changes made to the original version. You should upload this as a separate file labeled 'Revised Manuscript with Track Changes'.
- An unmarked version of your revised paper without tracked changes. You should upload this as a separate file labeled 'Manuscript'.

Guidelines for resubmitting your figure files are available below the reviewer comments at the end of this letter.

As the corresponding author, your ORCID iD is verified in the submission system and will appear in the published article. PLOS supports the use of ORCID, and we encourage all coauthors to register for an ORCID iD and use it as well. Please encourage your coauthors to verify their ORCID iD within the submission system before final acceptance, as unverified ORCID iDs will not appear in the published article. *Only*  the individual author can complete the verification step; PLOS staff *cannot*  verify ORCID iDs on behalf of authors.

We look forward to receiving your revised manuscript.

Kind regards,

Alassane Dicko

Academic Editor

PLOS Global Public Health

Journal Requirements:

1. Please ensure that your Ethics Statement is available in its entirety at the beginning of your Methods section, under a subheading 'Ethics Statement'.

2. We ask that a manuscript source file is provided at Revision. Please upload your manuscript file as a .doc, .docx, .rtf or .tex.

3. We do not publish any copyright or trademark symbols that usually accompany proprietary names, eg (R), (C), or TM  (e.g. next to drug or reagent names). Please remove all instances of trademark/copyright symbols throughout the text, including "Orion QuickRead go®" on page 7.

4. We have noticed that you have uploaded Supporting Information files, but you have not included a list of legends. Please add a full list of legends for your Supporting Information files after the references list.

If the reviewer comments include a recommendation to cite specific previously published works, please review and evaluate these publications to determine whether they are relevant and should be cited. There is no requirement to cite these works unless the editor has indicated otherwise.

Please review your reference list to ensure that it is complete and correct. If you have cited papers that have been retracted, please include the rationale for doing so in the manuscript text, or remove these references and replace them with relevant current references. Any changes to the reference list should be mentioned in the rebuttal letter that accompanies your revised manuscript. If you need to cite a retracted article, indicate the article’s retracted status in the References list and also include a citation and full reference for the retraction notice.

Additional Editor Comments (if provided):

This manuscript reports the findings of a well-conducted cluster-randomized trial evaluating the effect of a single dose of azithromycin on malaria prevalence and parasite density among infants in Mali. The study addresses an important research question and adds to the growing body of evidence on the potential impact of azithromycin on malaria. Overall, this is a high-quality manuscript that is suitable for publication with minor revisions are required to further improve the manuscript. The authors are encouraged to carefully address the specific comments provided by the reviewers to strengthen the manuscript prior to final acceptance.

[Note: HTML markup is below. Please do not edit.]

Reviewers' comments:

Reviewer's Responses to Questions

**Comments to the Author**

1. Does this manuscript meet PLOS Global Public Health’s publication criteria?

Reviewer #1: Partly

Reviewer #2: Yes

Reviewer #3: Yes

**********

2. Has the statistical analysis been performed appropriately and rigorously?

Reviewer #1: No

Reviewer #2: I don't know

Reviewer #3: Yes

**********

3. Have the authors made all data underlying the findings in their manuscript fully available (please refer to the Data Availability Statement at the start of the manuscript PDF file)?

The PLOS Data policy

Reviewer #1: Yes

Reviewer #2: Yes

Reviewer #3: No

**********

4. Is the manuscript presented in an intelligible fashion and written in standard English?

Reviewer #1: Yes

Reviewer #2: Yes

Reviewer #3: Yes

**********

Reviewer #1: This study examines azithromycin treatment in a CRT substudy, hypothesizing that azithromycin reduces malaria prevalence, parasite load, and improves hemoglobin concentration. However, the analyses are not presented clearly enough to indicate the expected statistical power with the current sample size. Ultimately, no results reach statistical significance, and the manuscript appropriately acknowledges the insufficient power.

Additional details are needed regarding the regression analyses in the statistical methods. The tables and figures are well designed.

More specific comments below.

Abstract: The conclusion of the abstract should be revised. I do not agree with the statement that the results are consistent with the hypothesis.

Methods

Lines 96-98: The statement that data were collected during a single MDA should be clarified as it was the fourth round of a set of MDA. This part requires clarification.

Effect size 0.2-0.3 SD. Since three different outcomes are reported, specify which effect size refers to each outcome.

This also requires clarification.

Did the pilot study provide estimates for baseline and mean parasite load?

As this is a substudy, the Methods section should report the cluster size for each arm.

Also, it may be best to evaluate the expected power using the known cluster sizes, number of clusters, baseline prevalence, and other relevant measures. Data from the pilot study could be used for these estimates.

Statistical analysis: Logistic regression was used to model outcome prevalence, but the analytic approach for parasite load and hemoglobin concentration should also be specified clearly in the Methods section.

Results

Graphics and tables are well prepared. The results indeed do not indicate significant effects.

Discussion

Lines 277-279: This statement is speculative and should be removed from the discussion.

Reviewer #2: This manuscript describes findings from a sub-study nested within a cluster randomized controlled trial in Mali, where the sub-study aimed to assess if azithromycin mass treatment for infants has any effect on malaria prevalence and mean parasitemia in infants. The study was intended to assess if changes in malaria burden are an intermediate factor which is attributable for changes in all cause mortality observed in other studies after azithromycin mass treatment. However, since no effect of azithromycin on ACCM was observed in the main trial, it is perhaps not surprising that no significant effect of azithromycin on malaria was observed. Nevertheless, this research merits publication, is generally well-designed and presented, and contributes to the evolving evidence base on this theme.

1. Line 50. I recommend modifying here and throughout the rest of the paper to clarify between “malaria” (generally used as an indication of clinically-evident or symptomatic disease) and Plasmodium infection (a more specific description of the outcomes actually presented here).

2. Line 81. This sentence seemed to directly contradict the previous paragraph, where you have described multiple other trials of azithromycin with malaria-related outcomes.

3. Line 89. It may be informative for readers who are not familiar with the LAKANA trial to state here or in the next paragraph if this substudy was completed in all trial clusters, or only a subset. Line 128 is also slightly ambiguous and hints that this study was done in a subset of the most accessible clusters.

4. Line 184. It is not clear which outcome was used for the sample size calculation. What was the anticipated prevalence of P. falciparum by PCR in this population?

5. Lines 190-192. Presumably the mixed-effects model used individual level data, with the outcome being positive/negative status by PCR? I would argue that if this is the case, it may be clearer to describe the outcome as “P. falciparum infection by PCR” rather than prevalence which is an aggregate measure at the cluster level. Furthermore, I assume the model uses the PCR result at the 14-day follow-up as the outcome, and the PCR result at baseline as a covariate? This detail would be helpful to include.

6. Table 1. I believe that the notation for the Mean parasite load should be 10^-3 ng/ul, not 10^3. Please check this throughout the paper.

7. Methods/results. Please include a statement somewhere in these sections describing the dates of data collection, and how these dates relate to the usual seasonal transmission period in Mali. The low SMC coverage suggests that data collection was done during the dry season?

8. Line 236. Did you collect data on how many of the children with severe anemia that were referred to the health center actually completed this referral, and if this referral rate differed between arms? This is a potential bias in your interpretation of azithromycin effects on Hb.

9. Table 2. I’m not sure what is meant by “general” prevalence of malaria. Suggest changing this to “baseline prevalence of P. falciparum by PCR”. Similarly, I recommend modifying “prevalence of malaria among baseline malaria positives” to something like “Day 14 prevalence of P. falciparum by PCR among those PCR-positive at baseline”.

10. Lines 255-256. This model result is very surprising considering the descriptive results reported earlier in this paragraph, which state that the mean parasite density among PCR positives is essentially unchanged in the azithromycin group (0.0366 at baseline, 0.0363 at day 14) while the parasite densities reduced in the placebo arm among malaria positives (0.0642 at baseline, 0.0510 at day 14). I may have misunderstood the data which is being used in the model, but this indicates some further explanation is required to ensure to either explain how the results in the adjusted model are so different from the descriptive analysis, or explain if there is a different population assessed in the model.

11. Line 266. It is difficult to interpret or agree with this statement since the assumed prevalence is not reported in the sample size section, suggesting that prevalence was not a contributing factor in the calculation.

12.Lines 274. As with my earlier comment, the 85% reduction in parasite load among baseline positive infants does not seem to agree with description of the results, suggesting substantial confounding or imbalance that is being accounted for in the adjusted model.

13. Lines 298-306. This may be a suitable place to discuss whether exclusion of children identified as severely ill from trial was a potential limitation. My expectation is that for studies with ACCM as the outcome, these most vulnerable children are the ones that would benefit most from this kind of intervention, whether the effect is mediated through malaria parasitemia reduction or some other mechanism. Did these trials all exclude severely ill children, similar to the LAKANA trial?

14. Lines 318. The statement “ongoing seasonal malaria outbreak” isn’t clear. Does this mean a usual seasonal malaria transmission setting, but during the seasonal peak (implying this study was done in the low transmission season)? Or does this relate to an epidemic, where transmission accelerates beyond the usual expected level?

15. Line 323. I don’t agree that you have definitively shown that the reason for the null result was the low prevalence of malaria. It seems that azithromycin did not have a substantial effect on clearing parasites among those who were infected, which aligns with the Cochrane review showing that azithromycin has only a weak antimalarial effect.

Reviewer #3: 1. Question: was a sensitivity analysis done regarding the missing village?

2. What are the restrictions on data availability for the repository? I do not find anything unreasonable about the way the data are being provided.

**********

what does this mean?). If published, this will include your full peer review and any attached files.

**Do you want your identity to be public for this peer review?**  If you choose “no”, your identity will remain anonymous but your review may still be made public.

For information about this choice, including consent withdrawal, please see our Privacy Policy

Reviewer #1: No

Reviewer #2: No

Reviewer #3: No

**********

[NOTE: If reviewer comments were submitted as an attachment file, they will be attached to this email and accessible via the submission site. Please log into your account, locate the manuscript record, and check for the action link "View Attachments". If this link does not appear, there are no attachment files.]

# Author comment: Author response to Decision Letter 1

**DOI:** 10.1371/journal.pgph.0006518.r003

20 Jul 2026

Attachment Submitted filename: Response to Reviewers.docx

# Editor report: Decision Letter 1

**DOI:** 10.1371/journal.pgph.0006518.r004

**Authors:** Alassane Dicko

5 Aug 2026

A single dose of azithromycin versus placebo for malaria in infants: A cluster-randomized trial

PGPH-D-26-01585R1

Dear Dr. Ihamuotila,

We are pleased to inform you that your manuscript 'A single dose of azithromycin versus placebo for malaria in infants: A cluster-randomized trial' has been provisionally accepted for publication in PLOS Global Public Health.

Before your manuscript can be formally accepted you will need to complete some formatting changes, which you will receive in a follow up email. A member of our team will be in touch with a set of requests.

Please note that your manuscript will not be scheduled for publication until you have made the required changes, so a swift response is appreciated.

IMPORTANT: The editorial review process is now complete. PLOS will only permit corrections to spelling, formatting or significant scientific errors from this point onwards. Requests for major changes, or any which affect the scientific understanding of your work, will cause delays to the publication date of your manuscript.

If your institution or institutions have a press office, please notify them about your upcoming paper to help maximize its impact. If they'll be preparing press materials, please inform our press team as soon as possible -- no later than 48 hours after receiving the formal acceptance. Your manuscript will remain under strict press embargo until 2 pm Eastern Time on the date of publication. For more information, please contact globalpubhealth@plos.org.

Thank you again for supporting Open Access publishing; we are looking forward to publishing your work in PLOS Global Public Health.

Best regards,

Alassane Dicko

Academic Editor

PLOS Global Public Health

***********************************************************
